# Putative palm pathogens: Novel species and new host records of leaf disease-associated microfungi (Ascomycota) on *Nypa fruticans* in Thailand

**DOI:** 10.3897/mycokeys.130.175665

**Published:** 2026-04-06

**Authors:** Herbert Dustin R. Aumentado, Ruvishika S. Jayawardena, Carlo Chris Apurillo, Chayanard Phukhamsakda, E. B. Gareth Jones, Eric H. C. McKenzie, Kevin D. Hyde, Long-Feng Yu, Yong Wang

**Affiliations:** 1 College of Biotechnology and Engineering, West Yunnan University, Lincang, Yunnan 677000, China College of Biotechnology and Engineering, West Yunnan University Lincang China; 2 School of Science, Mae Fah Luang University, Chiang Rai 57100, Thailand Center of Excellence in Fungal Research, Mae Fah Luang University Chiang Rai Thailand; 3 Center of Excellence in Fungal Research, Mae Fah Luang University, Chiang Rai 57100, Thailand School of Science, Mae Fah Luang University Chiang Rai Thailand; 4 College of Agriculture, Key Laboratory of Agricultural Microbiology of Guizhou Province, Guizhou University, Guiyang, Guizhou 550025, China Philippine Science High School-Eastern Visayas Campus Palo Philippines; 5 Department of Science and Technology–Center for Research in Science and Technology (CReST), Philippine Science High School-Eastern Visayas Campus, Palo, Leyte, 6500 Philippines King Saud University Riyadh Saudi Arabia; 6 Department Microbial Drugs, Helmholtz Centre for Infection Research (HZI), Inhoffenstrasse 7, 38124, Braunschweig, Germany Nantgaredig Southsea United Kingdom; 7 Department of Botany and Microbiology, College of Science, King Saud University, P.O. Box 2455, Riyadh 11451, Saudi Arabia Manaaki Whenua-Landcare Research Auckland New Zealand; 8 Nantgaredig, Southsea, Hants., UK College of Agriculture, Key Laboratory of Agricultural Microbiology of Guizhou Province, Guizhou University Guiyang China; 9 Manaaki Whenua-Landcare Research, Auckland, New Zealand Helmholtz Centre for Infection Research (HZI) Braunschweig Germany

**Keywords:** 2 new host records, 2 novel species, Arecaceae, mangrove fungi, multi-locus phylogeny, Mycosphaerellaceae, Sporocadaceae, synonyms

## Abstract

*Nypa fruticans* is a true mangrove species that provides numerous economic and ecological contributions to the mangrove ecosystem. Various studies have dealt with fungi associated with mangrove palms; however, most of these are saprobes, and few studies have explored disease-associated microfungi. In this study, fungi were isolated from symptomatic leaves of *Nypa fruticans* collected in mangrove estuaries in Prachuap Khiri Khan, Thailand. From the 17 isolates obtained, four species were identified using combined morpho-cultural characteristics and molecular phylogenetic analyses; different subsets of loci (from ITS, LSU, *ACT*, *CAL*, *CHS*-1, *GAPDH*, *HIS3*, *RPB2*, *TEF1*-α, and *TUB2*) were used per species. Two new species, *Brunswickiella nypae* and *Pestalotiopsis nypae*, are described, and two new host records of *Colletotrichum siamense* and *Diaporthe arecae* are given. We also provide an updated worldwide list of reported microfungi on *N. fruticans*. This study addresses a gap in the leaf disease-associated microfungi on *N. fruticans* and provides a comprehensive overview of the diversity of fungal species on this brackish water palm. By documenting fungal diversity and potential pathogens, our findings support biodiversity conservation and sustainable ecosystem management, directly contributing to SDG 14 (Life Below Water) and SDG 15 (Life on Land) and indirectly to SDG 12 (Responsible Consumption and Production) and SDG 13 (Climate Action) through the protection of mangrove health and resilience.

## Introduction

Mangroves are trees and shrubs that thrive in estuarine and coastal regions of tropical and subtropical areas worldwide (Spalding et al. 2010; UNEP 2014; Akram et al. 2023). They are ecologically and economically significant to coastal ecosystems, providing shoreline stabilization, nutrient cycling, and blue carbon storage, as well as ecotourism and habitats for diverse flora and fauna (Lee et al. 2014; Osorio et al. 2016; Koh et al. 2018; Das et al. 2022; Akram et al. 2023). Among mangrove species, *Nypa fruticans* (Arecaceae), commonly known as the mangrove palm or nipa palm, is the sole palm species that is considered a true mangrove taxon (Joshi et al. 2006; Nugroho et al. 2020). Other palm species such as *Calamus erinaceus*, *Caryota urens*, *Cocos nucifera*, *Licuala spinosa*, *Livistona saribus*, *Livistona rotundifolia*, *Oncosperma tigillarium*, and *Phoenix paludosa* are often found near mangrove habitats and estuaries, but they are classified as mangrove associates (Giesen et al. 2007). *Nypa fruticans* is a distinctive mangrove species occurring throughout the tropical Indo–West Pacific region (Hossain and Islam 2015; Robertson et al. 2020). This palm also plays an important role in supporting coastal livelihoods. Its leaves are widely used for thatching and weaving, and the fruit endocarp for traditional medicine, while its sap is a source of sugar, alcohol, vinegar, and biofuel (Joshi et al. 2006; Hossain and Islam 2015; Cheablam and Chanklap 2020; Moonrungsee et al. 2022).

Fungi are vital to mangrove ecosystems, serving as decomposers, endophytes, and pathogens (Thatoi et al. 2013; Apurillo et al. 2019; Devadatha et al. 2021; Hyde et al. 1998a, 2023). Fungi have been reported from diverse substrates of *N. fruticans*, including leaves, petioles, and decaying tissues, where they contribute to nutrient recycling and plant health dynamics (Loilong et al. 2012; Sarma and Hyde 2018; Devadatha et al. 2021). Pathogenic fungi associated with palms can cause foliar diseases such as leaf spots (Pereira and Phillips 2023, 2025), which may result in the loss of photosynthetic capability and limit the utility of leaves for traditional and economic purposes (Udayanga et al. 2020). Despite the ecological and economic significance of *N. fruticans*, the diversity of fungi associated with its leaf diseases remains poorly characterized.

The fungi associated with leaf spot symptoms on *N. fruticans* are underexplored, and many species remain undescribed. Documenting and characterizing these fungi are crucial for advancing our understanding of their taxonomy, ecology, and potential impacts on mangrove health. This study aims to identify and describe microfungi associated with leaf diseases of *N. fruticans* in various estuaries in Thailand, using a polyphasic approach that integrates morpho-cultural characteristics and multi-locus phylogenetic analyses. Furthermore, the study aims to update the records of microfungi associated with *N. fruticans* to provide a more comprehensive understanding of their biology within mangrove ecosystems.

## Materials and methods

### Sample collection and fungal isolation

Symptomatic leaves were collected from *Nypa fruticans* at various mangrove estuaries in Prachuap Khiri Khan, Thailand, from February 2023 to May 2024. Isolation of taxa was performed following the tissue isolation protocol described by Senanayake et al. (2020). Pure axenic cultures were maintained on PDA (Hardy Diagnostics, Santa Maria, California) at 28 °C, 12 h light/12 h dark, for subsequent observation. *Diaporthe* isolates that did not sporulate on PDA medium were subcultured to nutrient-deficient/water agar (WA) medium with sterile alfalfa stems to induce sporulation. Colony diameter was measured after 7 days, while color, shape and form, and morphological characteristics were assessed after 1 and 4 weeks. Leaf symptoms and macromorphological characters (conidiomata) were examined using a LEICA-EZ4 stereomicroscope (Leica Microsystems (SEA), Singapore), and micromorphological features (conidioma, conidiophores, conidiogenous cells, and conidia (*n* = 30)) were examined using a Nikon DS-Ri2 compound microscope (Nikon Instruments, NY, USA) paired with a Canon EOS 600D digital camera (Canon, Tokyo, Japan). Morphological data were recorded using ImageJ v. 1.53t software (Schneider et al. 2012), and photographic plates were created with Adobe Photoshop 2020 (Adobe Systems, CA, USA). Dried cultures and leaf specimens were archived in the Mae Fah Luang University Herbarium (**MFLU**), with living cultures stored in slant cryogenic vials deposited in the Mae Fah Luang University Culture Collection (**MFLUCC**), Chiang Rai, Thailand. The new species names were submitted and registered in MycoBank (Crous et al. 2004a; Robert et al. 2013) and Faces of Fungi databases (Jayasiri et al. 2015).

### DNA extraction, PCR amplification, and sequencing

Fungal DNA was extracted from 1-month-old mycelia grown on PDA at room temperature (25 ± 2 °C) using the Omega Bio-tek EZNA DNA Extraction Kit (GA, USA), according to the manufacturer’s guidelines. Polymerase chain reactions (PCR) were carried out using the following primer pairs for each genus: ITS5/ITS4 (all strains); *Colletotrichum*: ACT512F/ACT738R (*ACTIN*), GDF/GDR (*GAPDH*), CHS-79F/CHS-354R (*CHS*), and T1/Btub4Rd (*TUB2*); for *Diaporthe*: CAL-228F/CAL-737R (*calmodulin*) and CYLH3F/H3-1b (*HIS3*); for *Diaporthe* and *Pestalotiopsis*: T1/Bt2b (*TUB2*) and EF1-728F/EF1-986R (*TEF1*-α); for *Brunswickiella*: LR0R/LR5 (LSU) and fRPB2-5F/fRPB2-7cR (*RPB2*) (Vilgalys and Hester 1990; White et al. 1990; Templeton et al. 1992; Glass and Donaldson 1995; O’Donnell and Cigelnik 1997; Carbone and Kohn 1999; Liu et al. 1999; Crous et al. 2004b). The PCR reaction was performed in a 25 µL volume, consisting of 12.5 µL of GoTaq Green MasterMix (Promega Corp., Madison, WI, USA), 1 µL of each primer (20 µM), 9.5 µL of deionized water, and 1 µL of template DNA. The thermal cycling program was executed in an Eppendorf Master Cycler X50s thermal cycler (Eppendorf, Hamburg, Germany) with specific conditions for each gene adapted from previous studies (Liu et al. 1999; Weir et al. 2012; Norphanphoun et al. 2019; Aumentado et al. 2026). The PCR products were then sequenced and sent to SolGent Co., Republic of Korea.

### Phylogenetic analyses

Chromatograms were quality-checked in Geneious Prime (Dotmatics, New Zealand), and the nucleotide sequences were assembled using SeqMan v. 7.1.0 software (DNASTAR, Madison, WI, USA). Sequence alignments were analyzed alongside sequences obtained from NCBI BLASTn (https://blast.ncbi.nlm.nih.gov/Blast.cgi) queries and through recently published articles (Suppl. material 1: table SS1–S4) (Crous et al. 2014; Pereira et al. 2023; Aumentado and Balendres 2024a, 2024b; Dissanayake et al. 2024; Razaghi et al. 2024; Aumentado et al. 2026; Gomdola et al. 2025; Miller et al. 2025; Wang et al. 2025). The One-click Fungal Phylogenetic Tool (OFPT) v. 1.8.2 (Zeng et al. 2023) was used to download sequences from NCBI GenBank, align and trim the taxa, and concatenate the five gene regions. First, the dataset of each gene region was independently aligned with the “auto” strategy (FFT-NS-1, FFT-NS-2, FFT-NS-I, or L-INS-I, based on data size) in MAFFT (Katoh and Standley 2013; Katoh et al. 2019) and trimmed with the “gappyout” method (based on gaps’ distribution) in TrimAl (Capella-Gutiérrez et al. 2009). BioEdit v. 7.0.9.0 (Hall 1999) was used for manual editing where needed. The best-fit nucleotide substitution models for each dataset (gene regions) were selected based on the Bayesian information criterion (BIC) from 22 common DNA substitution models with rate heterogeneity by ModelFinder (Kalyaanamoorthy et al. 2017). The datasets per genus were then concatenated with partition data for subsequent phylogenetic analyses.

Maximum likelihood (ML), maximum parsimony (MP), and Bayesian posterior probability analyses (PP) were performed using the CIPRES Science Gateway (https://www.phylo.org/portal2) (Miller et al. 2011). Maximum likelihood trees were constructed using RAxMLHPC2 on XSEDE with bootstrapping of 1000 replicates. The ML analyses utilized the GTR + GAMMA model. Maximum parsimony was performed using PAUP XSEDE (Swofford and Sullivan 2003). Bayesian posterior probability (PP) analyses employed a Markov chain Monte Carlo (MCMC) algorithm with MrBayes on XSEDE, involving four MCMC chains running for 1,000,000 generations and sampling at intervals of 100 generations. The first 25% of constructed trees were eliminated as burn-in, and the remaining trees were used to compute posterior probabilities (Ronquist et al. 2012). Phylograms were visualized with FigTree v. 1.4.4 (Rambaut and Drummond 2012) and formatted using Adobe Illustrator CC 22.0.0 (Adobe Systems, USA).

The concatenated alignment was used to infer the occurrence of recombination of the new *Pestalotiopsis* species and their closest relatives’ boundaries through the pairwise homoplasy index (PHI, Φw) test (Bruen et al. 2006), as demonstrated and detailed by Quaedvlieg et al. (2014), Jayawardena et al. (2021), and Maharachchikumbura et al. (2021) using SplitsTree4 (Huson 1998; Huson and Bryant 2006). If the PHI value is greater than 0.05 (Фw > 0.05), it suggests that no substantial recombination occurred in the dataset. A splits graph was used to illustrate the relationships between closely related species, with both the Log-Det transformation and splits decomposition options applied.

### Worldwide checklist of microfungi on *Nypa fruticans*

Previously reported microfungi on *Nypa fruticans* were compiled from relevant publications. Fungal classification was confirmed using fungal databases, including Index Fungorum, MycoBank (Crous et al. 2004a; Robert et al. 2013), and the Outline of Fungi (Hyde et al. 2024a, 2024b). Visualization was performed using a Sunburst chart in Python (v. 3.13) and the Plotly library (Plotly Technologies 2015).

## Results

**Figure 1.**
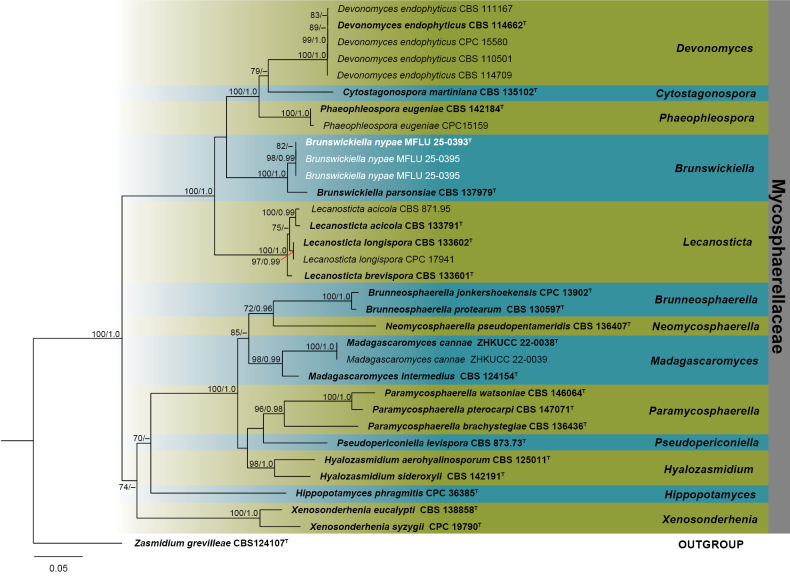
Phylogram generated from maximum likelihood analysis based on the concatenated LSU, ITS, and *RPB2* sequence data of selected genera of Mycosphaerellaceae. The tree topology of the ML analysis was identical to the PP analysis. Support values for maximum likelihood (ML) ≥ 70% and Bayesian posterior probabilities (PP) ≥ 0.90 are annotated at the nodes as ML/PP. The tree is rooted with *Zasmidium grevilleae* (CBS 124107). Type strains are in bold and are marked with a “^T^”, and newly identified isolates in this study are in white. Bar = 0.05 represents the estimated number of nucleotide substitutions per site per branch.

Seventeen fungal isolates were obtained from symptomatic *Nypa fruticans* leaves collected in mangrove estuaries of Prachuap Khiri Khan, Thailand. Phylogenetic and morphological analyses revealed four distinct genera: *Brunswickiella*, *Colletotrichum*, *Diaporthe*, and *Pestalotiopsis*. Six isolates belonged to the *Colletotrichum gloeosporioides* species complex, four to *Diaporthe* section Foeniculina, four to *Pestalotiopsis*, and three to *Brunswickiella*. The estimated nucleotide-substitution model parameters and tree statistics generated in the phylogenetic analyses are summarized in Suppl. material 1: table S5. The phylogenetic trees for each genus were constructed using a concatenated alignment of multilocus genotypes and strains. For *Brunswickiella*, the alignment comprised concatenated LSU–ITS–*RPB2* sequences of 33 taxa from genera of Mycosphaerellaceae, including an outgroup taxon of *Zasmidium grevilleae* (CBS 124107). This concatenated alignment was used for ML and PP analyses (Fig. 1). The best-scoring RAxML tree, following final optimization, revealed a likelihood value of −12537.354163. The alignment matrix comprised 2136 characters, of which 753 were unique patterns and 8.25% were undetermined characters. The three strains in this study (MFLU25-0393, MFLU25-0394, MFLU25-0395) clustered with *Brunswickiella parsonsiae* (CBS 137979) in the phylogenetic tree, forming a separate lineage with 100/1.0 ML and PP values, respectively (Fig. 1).

**Figure 2.**
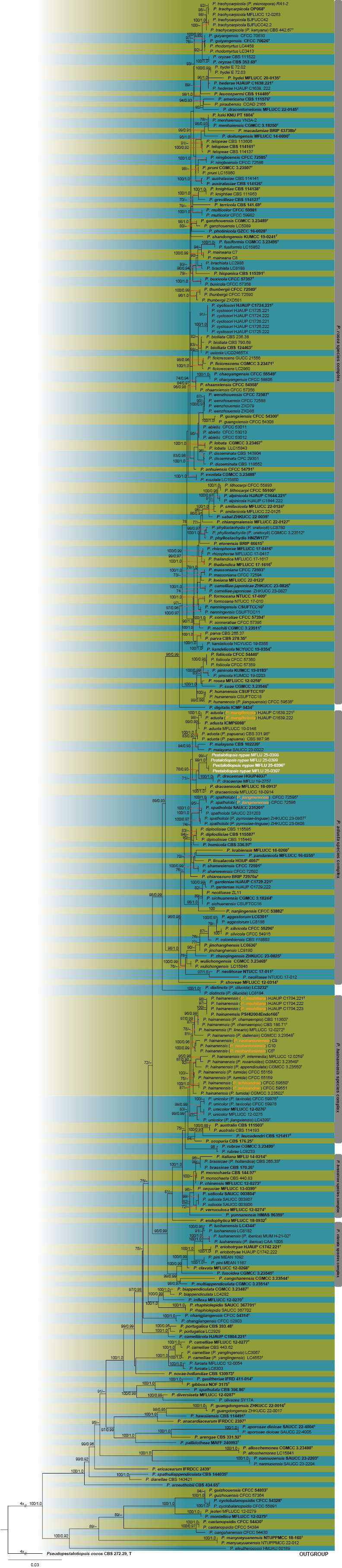
Phylogram generated from maximum likelihood analysis based on the concatenated ITS, *TEF1*α, and *TUB2* sequence data of *Pestalotiopsis*. The tree topology of the ML analysis was identical to the PP analysis. Support values for maximum likelihood (ML) ≥ 70% and Bayesian posterior probabilities (PP) ≥ 0.90 are annotated at the nodes as ML/PP. The tree is rooted with *Pseudopestalotiopsis cocos* (CBS 272.29). Type strains are in bold and are marked with a “^T,^” and newly identified isolates in this study are in white, and proposed synonyms are in orange. Bar = 0.03 represents the estimated number of nucleotide substitutions per site per branch.

In *Pestalotiopsis* strains, the alignment comprised concatenated ITS–*TEF1*α–*TUB2* sequences of 294 taxa, including an outgroup taxon of *Pseudopestalotiopsis cocos* (CBS 272.29). This concatenated alignment was used for ML and PP analyses (Fig. 2). The best-scoring RAxML tree, following final optimization, revealed a likelihood value of −21519.033644. The alignment matrix comprised 2108 characters, of which 1060 were unique patterns, 656 parsimony-informative, 207 singleton sites, and 1245 constant sites. Four isolates in this study (MFLU25-0396, MFLU25-0397, MFLU25-0398, and MFLU25-0399) grouped within the *P. adusta* species complex and clustered with *Pestalotiopsis dracaenae* (HGUP4037 and MFLU 19-2757) in the phylogenetic tree, forming a separate lineage with 78 ML and 0.93 PP values, respectively. They are also in a sister clade with *P. dracaenicola* (MFLUCC 18-0913, MFLUCC 18-0914) (Fig. 2).

**Figure 3.**
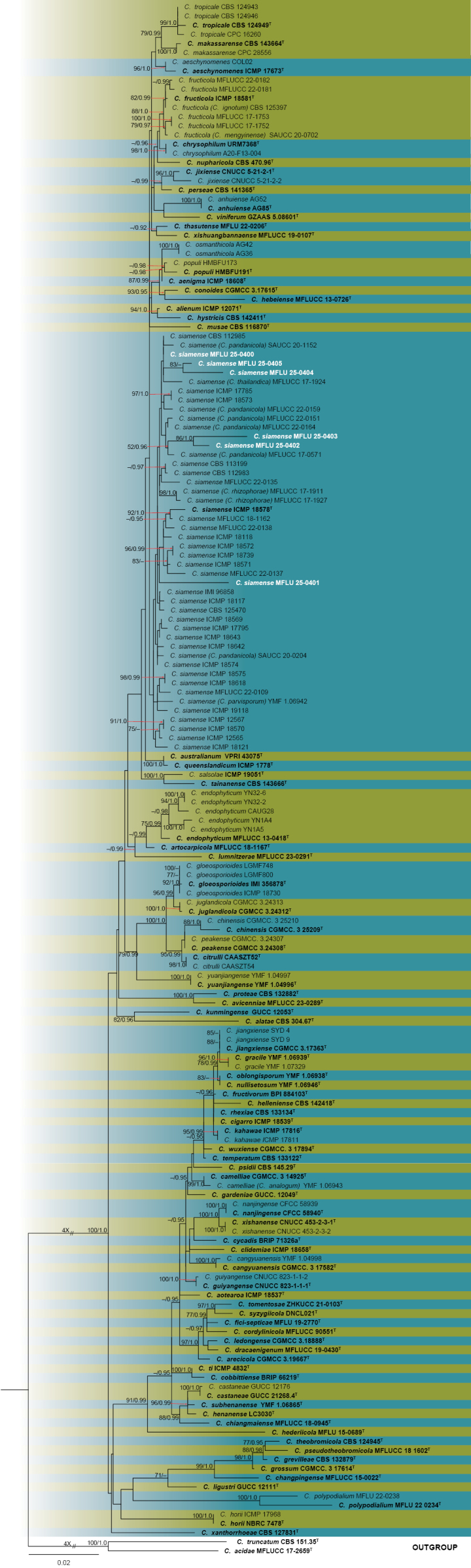
Phylogram generated from maximum likelihood analysis based on the concatenated ITS, *GAPDH*, *CHS*-1, *ACT*, and *TUB2* sequence data of the *Colletotrichum gloeosporioides* species complex. The tree topology of the ML analysis was identical to the PP analysis. Support values for maximum likelihood (ML) ≥ 70% and Bayesian posterior probabilities (PP) ≥ 0.90 are annotated at the nodes as ML/PP. The tree is rooted with *C. truncatum* (CBS 151.35) and *C. acidae* (MFLUCC 17-2659). Type strains are in bold and are marked with a “^T,^” and newly identified isolates in this study are in white. Bar = 0.02 represents the estimated number of nucleotide substitutions per site per branch.

In *Colletotrichum* strains, the alignment comprised concatenated ITS–*GAPDH*–*CHS*-1–*ACT*–*TUB2* sequences of 170 taxa of the *Colletotrichum gloeosporioides* species complex, including the outgroup taxa *C. acidae* (MFLUCC17-2659) and *C. truncatum* (CBS 151.35). This concatenated alignment was used for ML and PP analyses (Fig. 3). The maximum parsimony analysis comprised 1817 characters, of which 1034 characters were constant, 542 were parsimony-informative, and 241 were parsimony-uninformative. The resulting most parsimonious tree (tree length (TL) = 1594, consistency index (CI) = 0.586, retention index (RI) = 0.846, rescaled consistency index (RC) = 0.496, homoplasy index (HI) = 0.414) is shown in Fig. 3. The best-scoring RAxML tree, after final optimization, displayed a likelihood value of −13875.191638. The alignment matrix included 983 distinct patterns, with 8.80% gaps and undetermined characters. The six *Colletotrichum* strains all clustered with *C. siamense*, grouping with different strains of *C. siamense* with varied ML, MP, and PP support values. One strain, *C. siamense* (MFLU 25-0401), clustered closely with the *C. siamense* (ICMP 18578) type strain; MFLU 25-0402 and MFLU 25-0403 clustered with several *C. siamense* (= *C. pandanicola*) strains; and three strains of *C. siamense* (MFLU 25-0400, MFLU 25-0404, and MFLU 25-0405) grouped with *C. siamense* (*C. thailandica*) MFLUCC 17-1924, *C. siamense* (*C. pandanicola*) SAUCC 20-1152, and *C. siamense* CBS 112985 (Fig. 3).

**Figure 4.**
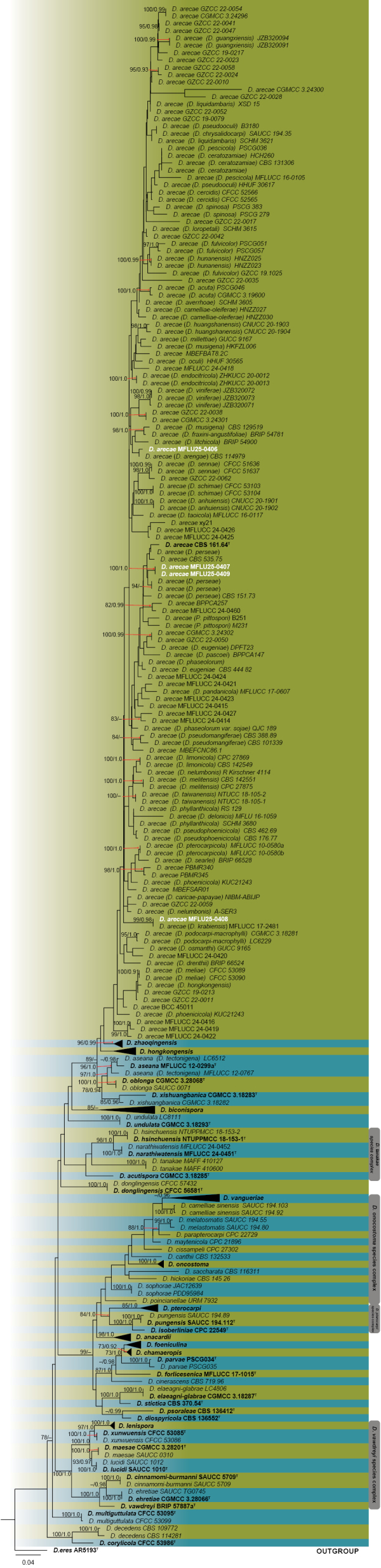
Phylogram generated from maximum likelihood analysis based on the concatenated ITS, *TEF1*α, *TUB2*, *CAL*, and *HIS3* sequence data of *Diaporthe* section Foeniculina. The tree topology of the ML analysis was identical to the PP analysis. Support values for maximum likelihood (ML) ≥ 70% and Bayesian posterior probabilities (PP) ≥ 0.90 are annotated at the nodes as ML/PP. The tree is rooted with *D. bohemiae* (CBS 143347) and *D. betulicola* (CFCC 51128). Type strains are in bold and are marked with a “^T,^” and newly identified isolates in this study are in white. Bar = 0.04 represents the estimated number of nucleotide substitutions per site per branch.

In *Diaporthe* strains, the alignment comprised concatenated ITS–*TEF1*α–*TUB2*–*CAL*–*HIS3* sequences of 298 taxa of section Foeniculina, including the outgroup taxon *D. eres* (AR5193). This concatenated alignment was used for ML and PP analyses (Fig. 4). The best-scoring RAxML tree, after final optimization, displayed a likelihood value of −37677.910951. The alignment matrix included 1462 distinct patterns, with 36.06% gaps and undetermined characters. Five *Diaporthe* strains clustered in *D. arecae* and its synonymous taxa with varied ML and PP support values. Two *D. arecae* strains (MFLU25-0407, MFLU25-0409) clustered closely with the *D. arecae* (CBS 161.64) type strain; one strain (MFLU25-0406) grouped with *D. arecae* (*D. arengae*) CBS 114979; and one strain (MFLU25-0408) grouped with *D. arecae* (*D. krabiensis*) MFLUCC 17-2481 with 99 ML and 0.98 PP support values (Fig. 4).

### Pairwise homoplasy index test and phylogenetic network analyses

**Figure 5.**
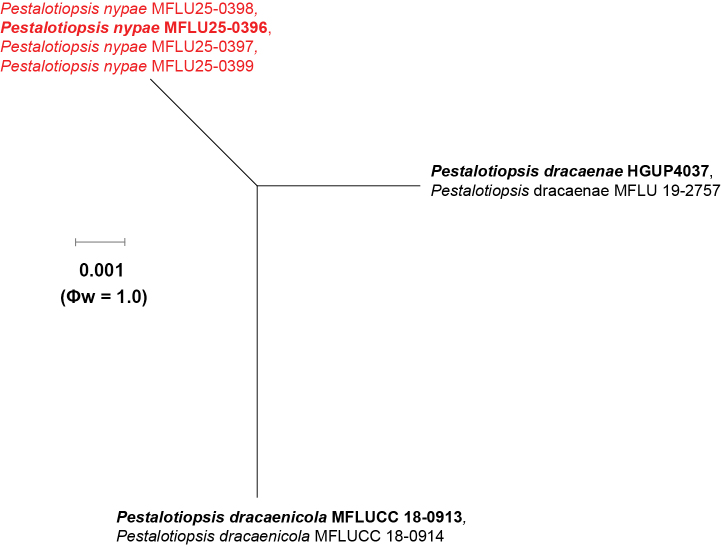
Split graph displaying the results of the pairwise homoplasy index (PHI) test of *Pestalotiopsis nypae* sp. nov. and closely related taxa using Log-Det transformation and splits decomposition options. Φw ≥ 0.05 from the PHI test signifies no substantial recombination in the dataset. The type species are indicated in bold, and the newly identified taxon and isolates are displayed in red.

The PHI test was conducted to assess the extent of recombination within the newly identified *Pestalotiopsis* species and their phylogenetically related species. As shown in Fig. 5, the PHI test indicated no statistically significant recombination (Фw = 1.0) between *Pestalotiopsis nypae* and the closely related taxa, *P. dracaenae* and *P. dracaenicola* (Fig. 5). The PHI test for *Brunswickiella* was not performed, as the genus consists of only two species, including the species introduced here. The nucleotide substitutions (1.15% in ITS and 6.64% in *RPB2*) and phylogenetic analyses (ML and PP) supported *B. nypae’s* distinctness from the type species, *B. parsonsiae*.

### Taxonomy

#### Brunswickiella nypae

Taxon classificationFungiMycosphaerellalesMycosphaerellaceae

Aumentado, Jayaward., E.B.G. Jones & K.D. Hyde sp. nov.

64671CE3-FAE7-5974-B85D-80F1E9DC0E94

Index Fungorum: IF904674

Figs 1, 6

##### Holotype.

MFLU25-0393.

##### Etymology.

The epithet refers to the host genus, *Nypa*, from which the species was isolated.

##### Description.

Associated with leaf spots of *Nypa fruticans*. ***Leaf spots*** initial rounded then expand to elongated and irregular in shape, brown with a darker brown margin. **Asexual morph**: On PDA, ***Conidiomata*** sporodochial, superficial, effuse, non-stromatic, center, confluent, pale pink becoming dark peach with hyaline hyphae; giving rise to conidiophores, conidiogenous cells, and conidia. Basal stroma poorly developed, hyaline. ***Conidiophores*** arising from the basal stroma or hyphae, hyaline, septate, consisting of a stipe and mostly branched conidiogenous cells. ***Conidiogenous cells*** 5.2–8 × 1.2–2 μm (6 × 1.8 μm), hyaline, smooth, subcylindrical, terminal and lateral, proliferating percurrently at apex. ***Conidia*** 6.6–9 × 1.8–2.5 μm (8 × 2.2 μm, *n = 30*, l/w: 3.64), solitary, hyaline, smooth, eguttulate, subcylindrical to narrowly fusoid-ellipsoidal, mostly straight, tapering to subobtuse apex and truncate hilum. **Sexual morph**: Not observed.

##### Culture characteristics.

Colonies on PDA reaching approximately 32 mm diam. after 4 weeks of incubation at room temperature; mycelium brown, turning black to olivaceous black with grayish margin; elevation flat to slightly concave with pale pink to peach conidial masses forming at the center, with an entire or undulate margin.

##### Specimen examined.

Thailand • Prachuap Khiri Khan, Pran Buri District, Fang Tha, Wang Pong, associated with leaf spots of *Nypa fruticans* (Arecaceae), 4 February 2023, H. D. Aumentado, dried culture permanently preserved in a metabolically inactive state NFS301B (MFLU25-0393, **holotype)**, **ex-type**MFLUCC 26-0121.

##### Additional specimen examined.

Thailand • Prachuap Khiri Khan, Pran Buri District, Fang Tha, Wang Pong, associated with leaf spots of *Nypa fruticans* (Arecaceae), 4 February 2023, H. D. Aumentado, dried culture permanently preserved in a metabolically inactive state NFS301AX (MFLU25-0394), NFS301CX (MFLU25-0395), living cultures MFLUCC 26-0122, MFLUCC 26-0123.

##### GenBank accession numbers.

ITS = PX612243, PX612244, PX612245; LSU = PX649205, PX649206, PX649207; RPB2 = PX692517, PX692518, PX692519.

##### Hosts and distributions.

Associated with leaf spot of *Nypa fruticans* in Thailand (this study).

##### Notes.

**Figure 6.**
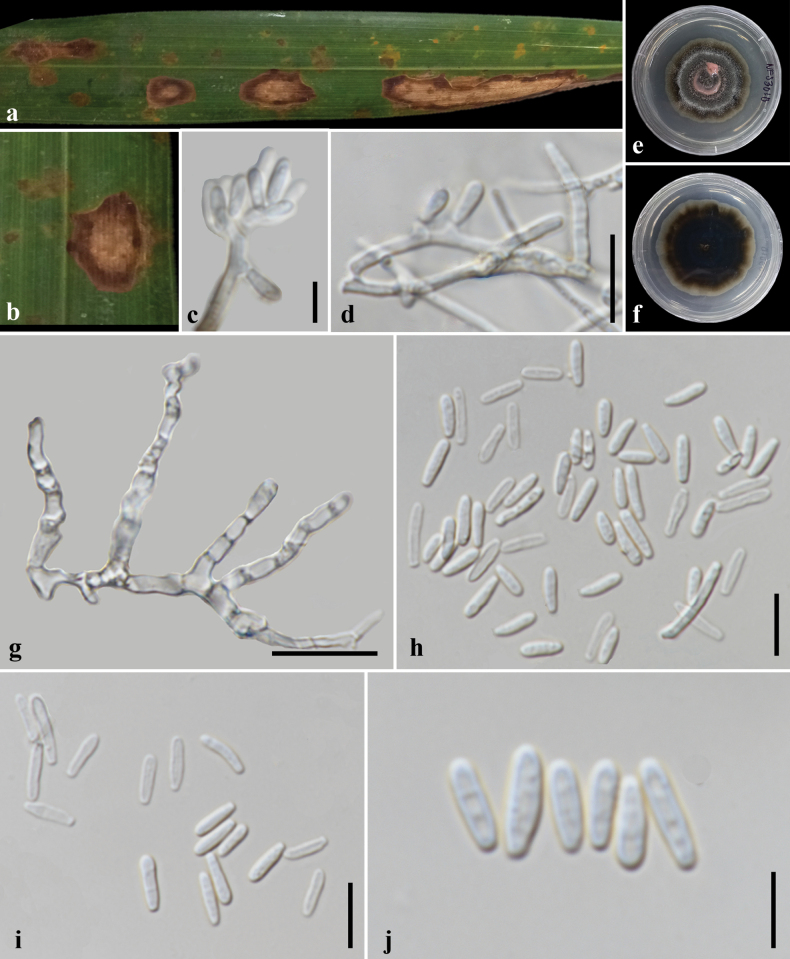
*Brunswickiella nypae* (MFLU25-0393, holotype). **a, b**. Leaf spot symptoms on *Nypa fruticans*; **c, d**. Conidia attachment on conidiophores and hyphae; **e, f**. Colony with sporodochia on PDA at 1 month post-incubation (obverse, reverse); **h–j** Conidia. Scale bars: 5 μm (**c, j**); 10 μm (**d, g–i**).

Based on the concatenated gene tree phylogeny, three strains of *Brunswickiella nypae* (MFLU25-0393, MFLU25-0394, and MFLU25-0395) formed a separate lineage with *B. parsonsiae* (CBS 137979) (Videira et al. 2017) with 100/1.0 ML and PP values (Fig. 1). Similar topologies were also observed in single-gene tree phylogenies with high support values (Suppl. material 2: figs S1–S3). *Brunswickiella nypae* (MFLU25-0393) is different based on ITS and *RPB2* loci from *B. parsonsiae* (CBS 137979), not including gaps, by nucleotide substitutions: 6/517 (1.15%) in ITS and 64/964 (6.64%) in *RPB2*. Morphological comparisons revealed that our species produced a different type of conidiomata (Fig. 6) compared to *B. parsonsiae* (CBS 137979) (Crous et al. 2014; Videira et al. 2017), as detailed in Table 1. *Brunswickiella nypae* produces larger conidia than *B. parsonsiae* (Crous et al. 2014). *Brunswickiella parsonsiae* was isolated from leaves of *Parsonsia straminea* (Apocynaceae) in Australia (Crous et al. 2014), while our species was isolated from leaf spots of *Nypa fruticans* in Thailand. Thus, based on morphological differences, host association, nucleotide differences, and molecular phylogenetic analyses, we introduce *Brunswickiella nypae* as a new species.

**Table 1.** Morphological comparison of *Brunswickiella nypae* and *Brunswickiella parsonsiae*.

Taxa	Colony on PDA	Conidiomata	Conidia		Reference
			Characters	Size	
***Brunswickiella nypae* MFLU25-0393**	Obverse: brown, turning black to olivaceous black with grayish margin; elevation flat to slightly concave with pale pink to peach conidial masses forming at the center, with an entire or undulate margin; Reverse: black	sporodochial superficial, effuse, non-stromatic, central, confluent, pale pink to dark peach with hyaline hyphae	solitary, hyaline, smooth, eguttulate, subcylindrical to narrowly fusoid-ellipsoidal, mostly straight, tapering to subobtuse apex and truncate hilum	8 × 2.2 μm, (6–9 × 1.8–2.5 μm), l/w ratio: 3.64	This study
*Brunswickiella parsonsiae* CBS 137979	Obverse: erumpent, folded, spreading, with even, lobed margins, surface pale olivaceous-gray; Reverse: smoke-gray	pycnidial immersed, black, with central ostiole and acervular convulated, stromatic, irregular, brown, verruculose hyphae	solitary, straight to slightly curved, hyaline, smooth, guttulate, subcylindrical to narrowly fusoid-ellipsoidal, widest in the middle	6–7 × 2 μm; l/w ratio: 3–3.5	Crous et al. 2014; Videira et al. 2017

#### Pestalotiopsis nypae

Taxon classificationFungiAmphisphaerialesPestalotiopsidaceae

Aumentado, Jayaward., E.B.G. Jones & K.D. Hyde sp. nov.

9E9B3F50-0BA9-542B-BA82-E5C70163F0E6

Index Fungorum: IF904675

Figs 2, 7

##### Holotype.

MFLU25-0396.

##### Etymology.

The epithet refers to the host genus, *Nypa*, from which the species was isolated.

##### Description.

Associated with leaf spots of *Nypa fruticans*. ***Leaf blight*** initially small, oval, grayish black necrotic spots that expand along the margin of the leaves. **Sexual morph**: Not observed. **Asexual morph**: On PDA, ***Conidiomata*** sporodochial, black, solitary, globose to subglobose, superficial. ***Conidiophores*** lageniform to fusoid, hyaline, septate, smooth- and thin-walled, collarette present. ***Conidiogenous cells*** indistinct. ***Conidia*** 17–25.7 µm × 4.7–6.2 µm (19.8 µm ± 2.8 × 5.4 µm ± 0.3), fusiform to clavate, straight to slightly curved, 3-4-septate; apical cell conical, 3.8–6.4 µm long (4.2 µm ± 0.7, *n = 30*), hyaline, with two to four appendages 8.2–19.3 µm long (14.8 µm ± 3.36, *n = 30*); three median cells concolorous brown, 11.8–16.4 µm long (9.6 µm ± 1.8, *n = 30*), septa and periclinal walls darker than rest of the cell, versicolored, wall rugose; basal cell obconic, with a truncate base, 2.2–5.6 µm long (3.3 µm ± 0.9, *n = 30*), hyaline or sometimes pale brown, thin- and smooth-walled; with one appendage 3.3–6.5 µm long (4.8 µm ± 0.8, *n = 30*).

##### Culture characteristics.

Colonies on PDA produced white, cottony, round, and filamentous mycelium with filiform margin. The reverse is initially white and becomes off-white to yellowish with time. It measures 30 mm diam. on PDA at 2 days after incubation (dai) at room temperatures (28 ± 2 °C), reaching 55 mm at 7 days after incubation.

##### Specimen examined.

Thailand • Prachuap Khiri Khan, Pran Buri District, Fang Tha, Wang Pong, associated with leaf spots of *Nypa fruticans* (Arecaceae), 4 February 2023, H. D. Aumentado, dried culture permanently preserved in a metabolically inactive state NFS306A (MFLU25-0396, **holotype)**, **ex-type**, MFLUCC 26-0124.

##### Additional specimen examined.

Thailand • Prachuap Khiri Khan, Pran Buri District, Fang Tha, Wang Pong, associated with leaf spots of *Nypa fruticans* (Arecaceae), 4 February 2023, H. D. Aumentado, dried culture permanently preserved in a metabolically inactive state MFLU25-0397, MFLU25-0398, MFLU25-0399, living cultures MFLUCC 26-0125, MFLUCC 26-0126, MFLUCC 26-0127.

##### GenBank accession numbers.

ITS = PX612246, PX612248, PX612247, PX612249; *TEF1*α = PX939448, PX939449, PX939450, PX939451; *TUB2* = PX939452, PX939453, PX939454, PX939455.

##### Hosts and distributions.

Associated with leaf spot of *Nypa fruticans* in Thailand (this study).

##### Notes.

**Figure 7.**
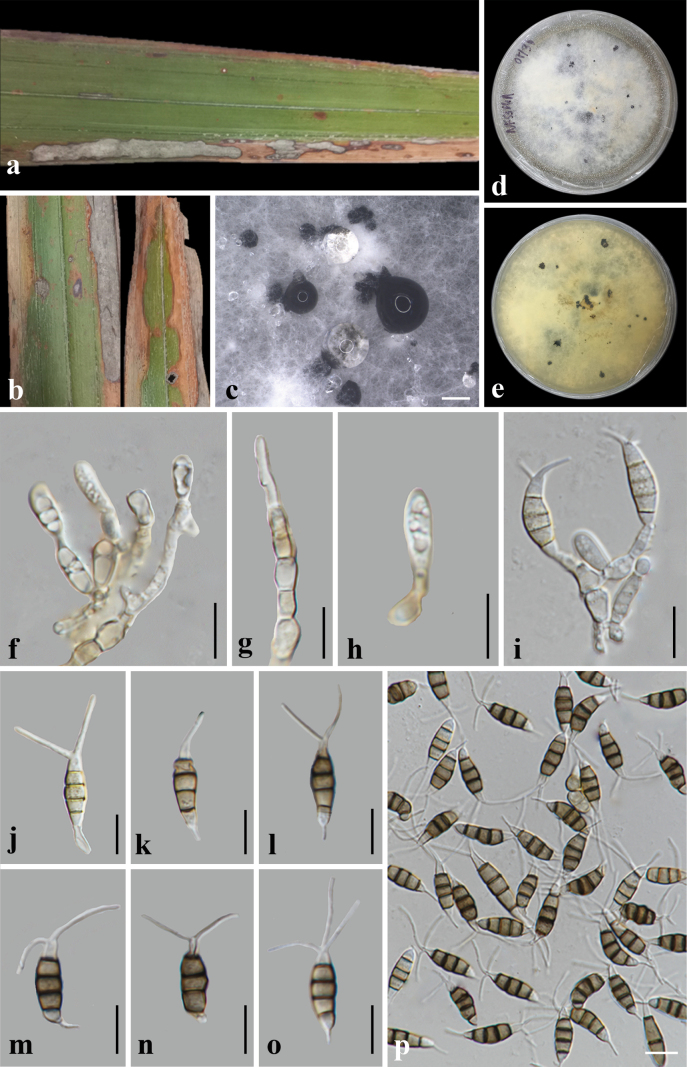
*Pestalotiopsis nypae* (MFLU25-0396, holotype). **a, b**. Leaf spot symptoms on *Nypa fruticans*; **c**. Sporodochia on PDA; **d, e**. Colony on PDA at 1 month post-incubation (obverse, reverse); **f–i**. Conidiophores, conidiogenous cells, and conidial attachment; **j–p**. Conidia. Scale bars: 500 mm (**c**); 10 μm (**f–p**).

Based on the concatenated gene tree phylogeny, four strains of *Pestalotiopsis nypae* (MFLU25-0396, MFLU25-0397, MFLU25-0398, and MFLU25-0399) grouped within the *P. adusta* species complex and formed a separate lineage with *P. dracaenae* (HGUP4037 and MFLU 19-2757) with 78 ML and 0.93 PP support values. In addition, *P. dracaenicola* (MFLUCC 18-0913, MFLUCC 18-0914) was grouped within the same clade with our species and *P. dracaenae* (Fig. 2). A similar topology was also observed in the *TUB2*-gene tree phylogeny (Suppl. material 2: figs S4–S6). *Pestalotiopsis nypae* (MFLU25-0396) is different based on ITS, *TEF1*α, and *TUB2* loci from *P. dracaenae* (HGUP4037), not including gaps, by nucleotide substitutions: 4/523 (0.78%) in ITS, 5/407 (1.23%) in *TEF1*α, and 10/391 (2.56%) in *TUB2*. It is different from *P. dracaenicola* (MFLUCC 18-0913), 4/523 (0.76%) in ITS, 6/405 (1.48%) in *TEF1*α, and 8/409 (1.96%) in *TUB2*. The PHI test indicated no significant recombination between *P. nypae* and its closely related species: *P. dracaenae* and *P. dracaenicola* (Fig. 5). Morphological comparisons revealed differences in conidial and appendage dimensions, as detailed in Table 2. *Pestalotiopsis nypae* produces smaller conidia and a larger l/w ratio than *P. dracaenae* (Ariyawansa et al. 2015) and *P. dracaenicola* (Chaiwan et al. 2020). *Pestalotiopsis nypae* produces longer apical appendages than *P. dracaenae* and *P. dracaenicola*, whereas the basal appendage of *P. nypae* is slightly longer (Fig. 7) than *P. dracaenae* and *P. dracaenicola* (Ariyawansa et al. 2015; Chaiwan et al. 2020). *Pestalotiopsis dracaenae* was isolated as an endophyte from leaves of *Dracaena fragrans* (Asparagaceae) in China (Ariyawansa et al. 2015); *P. dracaenicola* is saprobic on dead leaves of *Dracaena* sp. (Asparagaceae) in Thailand (Chaiwan et al. 2020), while our species was isolated in association with leaf spots of *Nypa fruticans* (Arecaceae) in Thailand. Thus, based on morphological differences, phylogenetic analyses, nucleotide differences, the PHI test, and host association, we introduce *Pestalotiopsis nypae* as a new species.

**Table 2.** Morphological comparison of *Pestalotiopsis nypae* with its closely related *Pestalotiopsis* species.

Taxa	Conidia	Appendages		Reference
	Size	Apical (no; size)	Basal	
***Pestalotiopsis nypae* MFLU25-0396**	19.8 × 5.4 µm, (17–25.7 µm × 4.7–6.2 µm), l/w ratio: 3.67	1-4; 14.8 µm ± 3.36, (8.2–19.3 µm)	4.8 µm ± 0.8, (3.3–6.5 µm)	This study
*P. dracaenae* HGUP4037	20.5 × 7.5 μm; l/w ratio: 2.73	2-4; 11 μm, (6.5–15.5 μm)	4.2 μm, (3–7 μm)	Ariyawansa et al. 2015
*P. dracaenicola*MFLUCC 18-0913	24 × 5 μm; l/w ratio: 4.8	1-3; 8.5 μm, (6–11 μm)	4 μm, (3–5 μm)	Chaiwan et al. 2020

### Synonymies in *Pestalotiopsis*

#### Pestalotiopsis adusta

Taxon classificationFungiAmphisphaerialesPestalotiopsidaceae

(Ellis & Everh.) Steyaert, Transactions of the British Mycological Society 36 (2): 82, 236 (1953)

6D1B39BD-A250-580B-836E-CC991EBC5A4E

= Pestalotiopsis mangifericola X.X. Luo & Jian Ma, in Luo, Liao, Zhang, Castañeda-Ruíz, Ma and Xu, MycoKeys 109: 231 (2024).
See Maharachchikumbura et al. (2012) for illustrations and descriptions of asexual morph. Sexual morph not reported. See Zhang et al. (2026) for other synonymies.

##### Notes.

Based on the multilocus phylogenetic analyses (ML and PP), *P. mangifericola* strains grouped within the *P. adusta* species complex and claded with the *P. adusta* type strain with high statistical support (99 ML and 0.99 PP) (Fig. 2). *Pestalotiopsis mangifericola* was introduced by Luo et al. (2024) from diseased leaves of *Mangifera indica* in China. Zhang et al. (2026) synonymized *P. papuana* with *P. adusta* using polyphasic methods, but *P. mangifericola* was not included in the analysis, as this species was recently introduced. Morphologically, *P. mangifericola*, *P. papuana*, and *P. adusta* have nearly overlapping phenotypic characteristics. Thus, based on our phylogenetic analyses and the synonymies proposed by Zhang et al. (2026), *P. mangifericola* is proposed as a synonym of *P. adusta*.

#### Pestalotiopsis spatholobi

Taxon classificationFungiAmphisphaerialesPestalotiopsidaceae

Z.X. Zhang, J.W. Xia & X.G. Zhang, Microorganisms 11 (7, no. 1627): 9 (2023)

A5BE837F-6D05-578C-95D7-3D3ABE83F8F9

= Pestalotiopsis jiangmenensis Q.C. Wang & X.D. Zhou, in Wang, Zhan, Sattar, Wang, Zhou, Eckhardt, Li, Liu, Xu and Zhou, IMA Fungus 16(e151614): 25 (2025).
See Zhang et al. (2023) for illustrations and descriptions of the asexual morph. Sexual morph not reported. See Zhang et al. (2026) for other synonymies.

##### Notes.

Based on the multilocus phylogenetic analyses, *P. jiangmenensis* strains grouped within the *P. adusta* species complex and claded with the *P. spatholobi* type strain and *P. spatholobi* (= *P. pyrrosiae-linguae*) with high statistical support (100 ML and 1.0 PP) (Fig. 2). *Pestalotiopsis jiangmenensis* was introduced by Wang et al. (2025) from diseased needles of *Pinus massoniana* in China. Zhang et al. (2026) synonymized *P. pyrrosiae-linguae* with *P. spatholobi* using polyphasic methods, but *P. jiangmenensis* was not included in the analysis as this species was recently introduced. Morphologically, *P. jiangmenensis*, *P. pyrrosiae*-*linguae*, and *P. spatholobi* have nearly overlapping phenotypic characteristics. Thus, based on our phylogenetic analyses and the synonymies proposed by Zhang et al. (2026), *P. jiangmenensis* is proposed as a synonym of *P. spatholobi*.

#### Pestalotiopsis hainanensis

Taxon classificationFungiAmphisphaerialesPestalotiopsidaceae

A.R. Liu, T. Xu & L.D. Guo, Fungal Diversity 24: 29 (2007)

CD326054-1F47-54A6-82C1-D03E0600C03D

= Pestalotiopsis machiliana X.X. Luo & Jian Ma, in Luo, Liao, Zhang, Castañeda-Ruíz, Ma and Xu, MycoKeys 109: 229 (2024).
= Pestalotiopsis neohantoniensis S.T. Mill. & C. Salgado, in Miller, Salgado-Salazar, Munk and Castlebury, Fungal Syst. Evol. 16: 276 (2025).
= Pestalotiopsis schisandrae R. Yuan & C.M. Tian, in Yuan, Peng, Li and Tian, Mycosystema 43(5, no. 230306): 13 (2024).
See Liu et al. (2007) for illustrations and descriptions of asexual morph. Sexual morph not reported. See Zhang et al. (2026) for other synonymies.

##### Notes.

Based on the multilocus phylogenetic analyses, *P. machiliana*, *P. neohantoniensis*, and *P. schisandrae* grouped within the same clade with the *P. hainanensis* type strain and recently synonymized taxa of *P. hainanensis* with high statistical support (Fig. 2). *Pestalotiopsis machiliana* was introduced by Luo et al. (2024) from diseased leaves of *Machilus pauhoi*, *P. schisandrae* was introduced by Yuan et al. (2024) from *Schisandra sphenanthera* in China, and *P. neohantoniensis* was introduced by Miller et al. (2025) from branches of *Tsuga canadensis* in the USA. Zhang et al. (2026) synonymized *P. chamaeropis*, *P. linearis*, *P. daliensis*, *P. intermedia*, *P. rosarioides*, *P. appendiculata*, and *P. tumida* with *P. hainanensis* using polyphasic methods, but *P. machiliana*, *P. neohantoniensis*, and *P. schisandrae* were not included in the analysis as they were recently introduced. Morphologically, *P. machiliana*, *P. neohantoniensis*, and *P. schisandrae*, and *P. hainanensis* have nearly overlapping phenotypic characteristics. Thus, based on our phylogenetic analyses and the synonymies proposed by Zhang et al. (2026), *P. machiliana*, *P. neohantoniensis*, and *P. schisandrae* are proposed as synonyms of *P. hainanensis*.

#### Colletotrichum siamense

Taxon classificationFungiGlomerellalesGlomerellaceae

Prihast., L. Cai & K.D. Hyde, (2009)

21D475D3-B16F-5322-98D0-ABDABABA78F1

Index Fungorum: IF515410

Facesoffungi Number: FoF03599

Figs 3, 8

##### Description.

Associated with leaf spots of *Nypa fruticans*. ***Leaf spots*** initially small, round, necrotic, then expand and elongate and turn irregularly round, brown with a darker brown margin. **Asexual morph**: On PDA, ***conidiomata*** pycnidial, solitary, black, sub-globose to globose, semi-submerged, bearing orange conidial masses. ***Conidia*** 9.1–13.1 × 3–4.4 μm (11.5 ± 1.1 × 3.9 ± 0.4 μm, *n* = 30), aseptate, hyaline, smooth, uni- to biguttulate, round to cylindrical, apex subrounded to rounded. ***Conidiophores*** hyaline, septate, unbranched, cylindrical to inflated, straight or slightly curved, tapering towards the apex. ***Conidiogenous cells*** are cylindrical to round, hyaline, smooth, and thin-walled. ***Setae*** light to dark brown, concoloured, smooth-walled to finely verruculose, 2–4-septate, 58.4–79.2, (69.8 μm ± 6.4, *n* = 10) long, some much longer than others. Base constricted, slightly swollen above the constriction, or cylindrical, tip slightly acute. **Sexual morph**: Not observed.

##### Culture characteristics.

Colonies on PDA media, reaching 44.5 mm, when incubated at room temperatures (28 ± 2 °C) for 7 days, produce different morphotypes: a) gray center and white at margin, raised colonies with abundant aerial mycelium and spore masses around the colony. The reverse is grayish and white. b) all white slightly raised colonies, dense, cottony with floccose aerial mycelium and yellowish reverse; c) grayish-white to dull olivaceous, flat colony with sparse aerial mycelium and scattered dark patches and dark gray and white reverse.

##### Specimen examined.

Thailand • Prachuap Khiri Khan, Pran Buri District, Fang Tha, Wang Pong, associated with leaf spots of *Nypa fruticans* (Arecaceae), 4 February 2023–January 2025, H. D. Aumentado, dried culture permanently preserved in a metabolically inactive state MFLU25-0400, MFLU25-0401, MFLU25-0402, MFLU25-0403, MFLU25-0404, MFLU25-0405; living cultures MFLUCC 26-0128, MFLUCC 26-0129, MFLUCC 26-0130, MFLUCC 26-0131, MFLUCC 26-0132, MFLUCC 26-0133.

##### GenBank accession numbers.

ITS = PX612250, PX612251, PX612252, PX612253, PX612254, PX612255; *GAPDH* = PX692532, PX692533, PX692534, PX692535, PX692536, PX692537; *CHS-1* = PX692520, PX692521, PX692522, PX692523, PX692524, PX692525; *ACT* = PX692526, PX692527, PX692528, PX692529, PX692530, PX692531; *TUB2* = PX939442, PX939443, PX939444, PX939445, PX939446, PX939447.

##### Hosts and distribution.

Cosmopolitan. Mangrove species: *Barringtonia asiatica*, *Rhizophora apiculata*. Arecaceae: *Areca catechu*, *Cocos nucifera*, *Chamaedorea elegans*, *Dypsis lutescens* (Talhinhas and Baroncelli 2023; Aumentado et al. 2026; Luangharn et al. 2026; Farr and Rossman 2026).

##### Notes.

**Figure 8.**
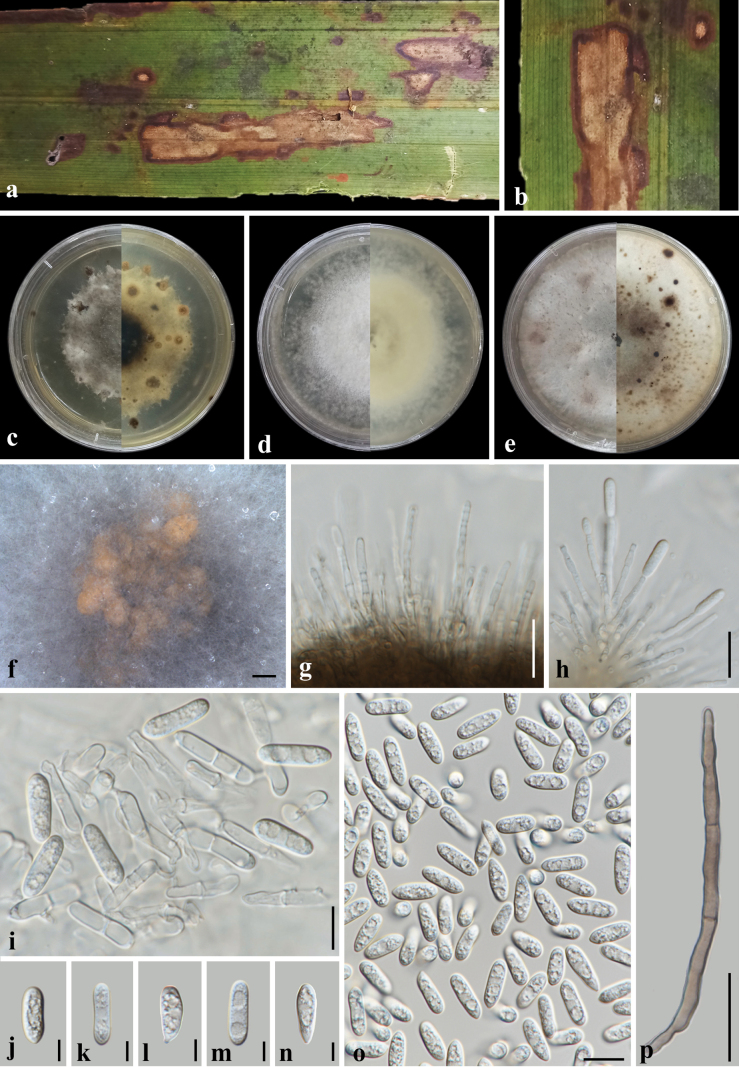
*Colletotrichum siamense* (MFLUCC 26-0128). **a, b**. Leaf spot symptoms on *Nypa fruticans*; **c–e**. Colony morphotypes on PDA at 1 month post-incubation (obverse, reverse); **f**. Conidial masses on PDA; **g–h**. Conidial attachment and conidiophores; **i**. Conidial anastomosis; **j–o**. Conidia; **p**. Setae. Scale bars: 500 mm (**f**); 20 μm (**g–h, p**); 10 μm (**i, o**); 5 μm (**j–n**).

Based on the concatenated gene tree phylogeny, all six strains of *Colletotrichum* grouped with *C. siamense* (including its synonymous taxa) with varied ML and PP support values (Fig. 3). Three *C. siamense* strains (MFLU25-0400, MFLU25-0404, and MFLU25-0405) grouped closely with *C. siamense* (*C. thailandica*) MFLUCC 17-1924, *C. siamense* (*C. pandanicola*) SAUCC 20-1152, and *C. siamense* CBS 112985; two *C. siamense* strains (MFLU25-0402 and MFLU25-0403) grouped with *C. siamense* (*C. pandanicola*) (MFLUCC 17-0571) with 52 ML and 0.96 PP support values; and one *C. siamense* strain (MFLU25-0401) claded basally with the type *C. siamense* (ICMP 18578). Morphological comparisons revealed that our strains produce no significant differences compared with the morphology of the ex-type strain and the strains identified in this study (Fig. 8). Thus, this is the first record of *C. siamense* associated with leaf spot of *N. fruticans*.

#### Diaporthe arecae

Taxon classificationFungiDiaporthalesDiaporthaceae

(H.C. Srivast., Zakia & Govindar.) R.R. Gomes, Glienke and Crous, Persoonia 31, 16 (2013)

52BE11CA-F7D0-5E01-A19C-65469E520764

MycoBank No: 802924

Index Fungorum: IF802924

Facesoffungi Number: FoF08405

Figs 4, 9

##### Description.

Associated with leaf spots of *Nypa fruticans*. ***Leaf spots*** necrotic, initially round, then expand and elongate to irregularly oval, brown with a darker brown margin. **Asexual morph**: On alfalfa stem on WA: ***conidiomata*** pycnidia 220–460 μm diam., solitary or aggregate, semi-immersed to submerged, black, sub-globose to globose, ostiolate with abundant pale white conidial cirri. ***Conidiophores*** 12.5–24.5 × 1.5–3.2 μm (20.2 ± 2.3 × 2.1 ± 0.5 μm, *n = 30*, l/w: 9.6), hyaline, cylindrical, septate, unbranched, straight, or slightly curved, tapering towards the apex. ***Conidiogenous cells***, 5.5–9 × 1.4–2.2 μm (7.8 ± 0.8 × 1.9 ± 0.2 μm, *n = 30*, l/w: 4.1), aseptate, hyaline, ellipsoidal to fusoid, and eguttulate. ***Alpha conidia*** 6.5–10.5 × 2–3.2 μm (8.2 × 2.5 μm, *n = 50*, l/w: 3.3), aseptate, hyaline, ellipsoidal to fusoid, multi-guttulate, base acute to acuminate. ***Beta conidia*** and ***gamma conidia*** not observed. **Sexual morph**: Not observed.

##### Culture characteristics.

Colonies on PDA media, reaching 52.5 mm, when incubated at room temperatures (28 ± 2 °C) for 7 days, produce three morphotypes: a) white, flat, filamentous, sparse aerial mycelium with circular and irregular margin, and reverse is orange yellow with visible submerged conidiomata; b) white, flat with entire margin, and reverse is yellow; c) gray, flat, floccose to woolly with entire margin and reverse is pale gray with concentric darker gray zones radiating from the center.

##### Specimen examined.

Thailand • Prachuap Khiri Khan, Pran Buri District, Fang Tha, Wang Pong, associated with leaf spots of *Nypa fruticans* (Arecaceae), 4 May 2024–January 2025, H. D. Aumentado, dried culture permanently preserved in a metabolically inactive state MFLU25-0406, MFLU25-0407, MFLU25-0408, MFLU25-0409; living cultures MFLUCC 26-0134, MFLUCC 26-0135, MFLUCC 26-0136, MFLUCC 26-0137.

##### GenBank accession numbers.

ITS = PX612258, PX612256, PX612259, PX612257; *TUB2* = PX933452, PX933454, PX933453, PX933455; *TEF1α* = PX939438, PX939440, PX939439, PX939441; *CAL* = PX933456, PX933458, PX933457, PX933459; *HIS3* = PX933460, PX933462, PX933461, PX933463.

##### Hosts and distribution.

Cosmopolitan. Mangrove species: *Annona glabra*, *Bruguiera* spp., *Kandelia candel*, *Lumnitzera* spp., *Millettia pinnata*, *Rhizophora* spp., *Sonneratia* spp., *Xylocarpus* spp.; Arecaceae: *Areca catechu*, *Arenga engleri*, *Chamaerops humilis*, *Chrysalidocarpus lutescens*, *Dypsis lutescens*, *Livistona chinensis*, *Phoenix canariensis*, *Phoenix paludosa*, *P. dactylifera*, *Trachycarpus fortunei* (Pereira et al. 2023; Aumentado et al. 2024b; Farr and Rossman 2026).

##### Notes.

**Figure 9.**
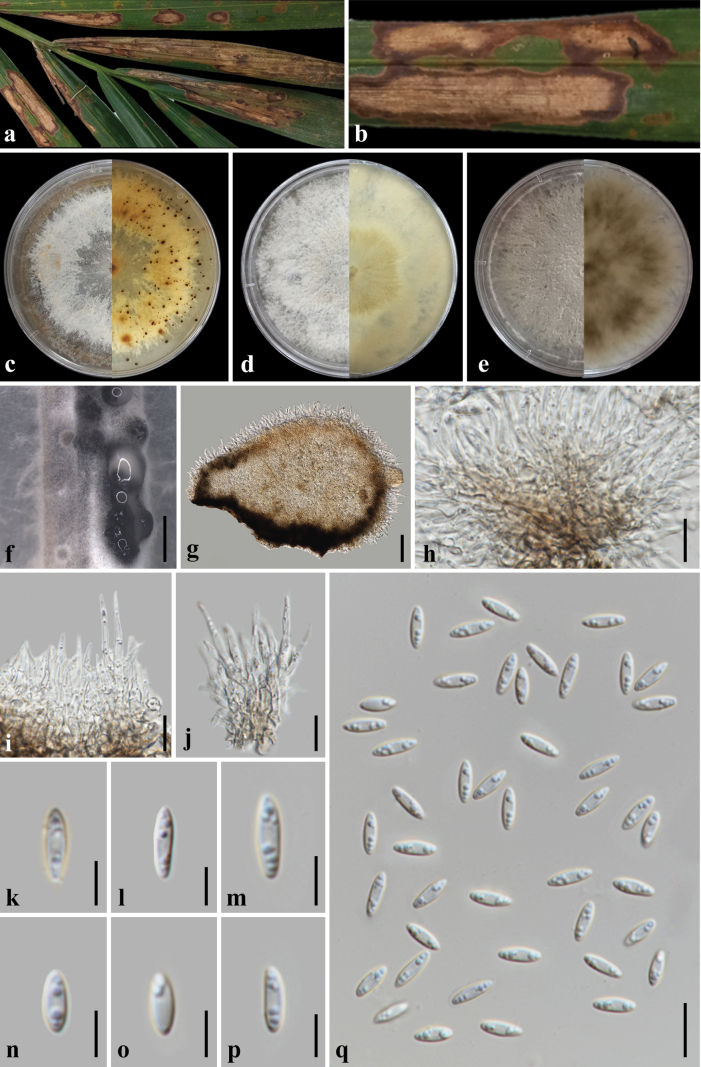
*Diaporthe arecae* (MFLUCC 26-0134). **a, b**. Leaf spot symptoms on *Nypa fruticans*; **c–e**. Colony morphotypes on PDA at 1 month post-incubation (obverse, reverse); **f**. Conidiomata on alfalfa stem in WA; **g**. Vertical section of conidioma; **h–j**. Conidial attachment and conidiophores; **k–q**. Conidia. Scale bars: 1 mm (**f**); 50 μm (**g**); 20 μm (**h**); 10 μm (**i–j, q**); 5 μm (**k–p**).

Based on the concatenated gene tree phylogeny, all four strains of *Diaporthe* grouped with *D. arecae* (including its synonymous taxa) with varied ML and PP support values (Fig. 4). Two *D. arecae* strains (MFLU25-0407, MFLU25-0409) grouped closely with the type strain *D. arecae* (CBS 161.64); one *D. arecae* strain (MFLU25-0406) grouped with *D. arecae* (*D. arengae*) (CBS 114979); and one *D. arecae* strain (MFLU25-0408) grouped with *D. arecae* (*D. krabiensis*) MFLUCC 17-2481 with 99 ML and 0.98 PP support values. Morphological comparisons revealed no significant differences between the ex-type strains and the isolates identified in this study (Fig. 9). Thus, this is the first record of *D. arecae* associated with leaf spot of *N. fruticans*.

### Worldwide fungal species checklist on *Nypa fruticans*

**Figure 10.**
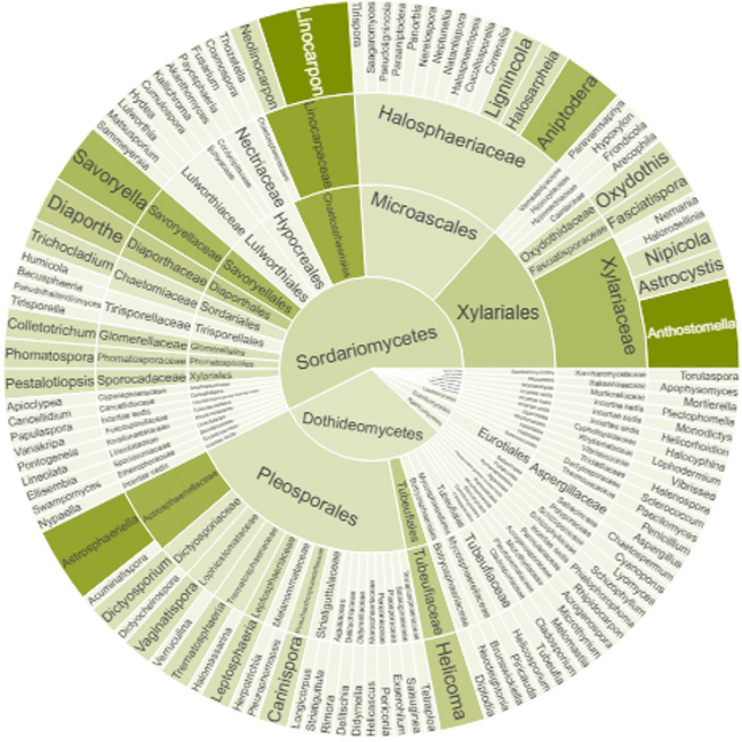
Sunburst chart of microfungi reported in association with *Nypa fruticans* worldwide, showing the dominance of Sordariomycetes. Concentric rings represent taxonomic rank from inner to outer: Class, Order, Family, and Genus. Segments are grouped hierarchically by taxon, and the color intensity indicates the number of reported taxa in each segment (darker = more taxa).

In this study, we compiled all the taxonomically valid data of fungi associated with *Nypa fruticans* worldwide using the USDA database and previously published articles (as detailed in Suppl. material 2: table S6). There are 144 species previously reported for *N. fruticans*, which are mostly saprobic mangrove fungi and are commonly found in Southeast Asian countries, such as Brunei Darussalam, Indonesia, Malaysia, the Philippines, and Thailand, where most mangrove forests are situated (Devadatha et al. 2021; Akram et al. 2023). With the addition of the four fungal species described in this study, there are currently a total of 148 fungal species associated with *N. fruticans*. Of these species, there are 110 genera, 66 families, 41 orders, 10 classes, and four phyla, including *incertae sedis*. Most of them belong to the family Halosphaeriaceae (14%), followed by Xylariaceae (8%) and Linocarpaceae (5%); order Pleosporales (20%), Xylariales (15%), and Microascales (14%); class Sordariomycetes (58%) and Dothideomycetes (30%); and phylum Ascomycota (93%) and Basidiomycota (3%) (Fig. 10).

## Discussion

This study identified and described microfungi associated with leaf spots of *Nypa fruticans* in estuaries in Thailand using a polyphasic approach. We documented and characterized two novel species and reported two new host records across four genera: *Brunswickiella*, *Colletotrichum*, *Diaporthe*, and *Pestalotiopsis*, adding to the growing list of disease-associated microfungi on *Nypa fruticans*. Phylogenetic analyses strongly support the distinctness of the two novel species from their closely related species, corroborated by nucleotide substitutions, a PHI test, and morphological characteristics. In the same manner, the identification of the two new host records is supported by combined morphological and molecular analyses.

Mangrove species have been associated with *Pestalotiopsis*, including *P. kandelicola*, *P. rhizophorae*, and *P. thailandica* from leaf spots of *Kandelia candel* and *Rhizophora apiculata* in China and Thailand (Hyde et al. 2020a, 2020b; Norphanphoun et al. 2019), and several *Pestalotiopsis* strains from leaf spots and dieback of *Sonneratia caseolaris* and *S. apetala* in Vietnam (Nguyen et al. 2021). Whereas there has been no reported *Brunswickiella* on mangroves. However, the two novel species in this study, *Brunswickiella nypae* and *Pestalotiopsis nypae*, are the first Mycosphaerellaceae and pestalotioid fungi associated with *N. fruticans*, respectively. Additionally, we proposed synonymization of five *Pestalotiopsis* taxa with three accepted *Pestalotiopsis* species (*i.e*., *P. adusta*, *P. spatholobi*, and *P. hainanensis*), which were not included in the analyses of Zhang et al. (2026), as they were only recently introduced. This study further supports the species delimitation within *Pestalotiopsis* species.

We report *C. siamense* on *N. fruticans* as a new host record. Our *C. siamense* strains in this study grouped with taxa synonymized with *C. siamense* strains, which was in congruence with the study of Liu et al. (2016) on *C. siamense*, being a single species rather than a species complex based on a polyphasic approach, including GCPSR, GMYC, PTP, PP, cross-fertility, morphological characters (Liu et al. 2016), and the PHI test (Aumentado et al. 2024a). Other mangrove species have also been reported with *C. siamense*, causing leaf spots and blight, such as *Barringtonia asiatica* and *Rhizophora apiculata* (Norphanphoun and Hyde 2023; Aumentado et al. 2024b). In addition, we report *D. arecae* on *N. fruticans* as a new host record. Various mangrove species have been reported with *D. arecae*, including *Annona glabra*, *Bruguiera* spp., *Kandelia candel*, *Lumnitzera racemosa*, *Millettia pinnata*, *Phoenix paludosa*, *Rhizophora* spp., *Sonneratia* spp., and *Xylocarpus* spp. (Cheng et al. 2008; Hu et al. 2018; Rajamani et al. 2018; Apurillo et al. 2019; Niaz et al. 2021; Zhang et al. 2021). Although *C. siamense* and *D. arecae* are relatively well-known and commonly reported pathogens (Talhinhas and Baroncelli 2023; Pereira et al. 2023; Pereira and Phillipps 2025) and have been recorded on other related hosts—including several Arecaceae species—this is the first report of their occurrence on *N. fruticans*. This may suggest that microfungi associated with *N. fruticans* diseases are still understudied and poorly reported. The occurrence of *C. siamense* and *D. arecae* on *N. fruticans* likely reflects either previously overlooked host associations due to limited sampling of mangrove phyllosphere or recent host shifts (Aumentado et al. 2026); however, additional sampling and population-level data are required to evaluate these hypotheses.

We provided an updated worldwide record of microfungi associated with *N. fruticans* (Fig. 10, Suppl. material 1: table S6). The prevalence of Sordariomycetes fungi on *N. fruticans* is likely due to most of the taxa in this class being lignicolous saprobes with a high capability for degrading lignocellulose and enduring fluctuating salinity, which are adept at colonizing and decomposing *N. fruticans* tissues (Hyde 1992a, 1992b, 1992c, 1992d; Pang et al. 2012; Chen et al. 2023). Nevertheless, many of these reports were published in the 1990s, and most lack adequate molecular data and phylogenetic support. This taxonomic pattern reflects other mangrove surveys (Calabon et al. 2021; Devadatha et al. 2021) and highlights both the strength of traditional mycology in the region and its limitations, which challenge modern mycology and phylogenetic reassessment. A single-gene region alone is insufficient for discriminating closely related species in these fungal groups (Tekpinar and Kalmer 2019; Chethana et al. 2021). Multi-locus gene phylogeny and deposition of linked specimens and ex-type cultures are essential for robust species delimitation and future taxonomic stability (Turland et al. 2018; Aime et al. 2021; Tedersoo et al. 2022).

Fungal taxa associated with *N. fruticans* fall into two groups: (1) saprobic mangrove fungi that are said to be host-specific (specialists) (Loilong et al. 2012; Sarma and Hyde 2018), including *Anthostomella nypae*, *Astrosphaeriella striatispora*, *Helicorhoidion nypicola*, *Linocarpon appendiculatum*, and *Nypaella frondicola* (Suppl. material 1: table S6); and (2) widely occurring fungi (generalists), *e.g*., *Akanthomyces muscarius* (entomopathogenic fungi), *Exserohilum rostratum* (human and plant pathogenic fungi), and *Schizophyllum commune* (wood-decay fungus) (Takemoto et al. 2010; Sharma et al. 2014; Wang et al. 2024), and *C. siamense* and *D. arecae* (cosmopolitan plant fungi) (Talhinhas and Baroncelli 2023; Pereira et al. 2023), which comprise 59% of the species isolated in this study. This might be due to differences in ecological specialization, physiological tolerance, dispersal ability, and sampling method. Many taxa reported as “*Nypa*-associated fungi” are saprobic intertidal fungi that appear host-restricted because they are adapted to the microhabitat provided by decaying *N. fruticans*, characterized by periodic inundation, high salt exposure, and a distinct substrate chemistry (Gadd 2007; Andlar et al. 2018). These specialists have morphological and ecological traits consistent with adaptation to marine or brackish conditions (*e.g*., salt-tolerant spore germination, specialized fruiting bodies on submerged tissue) and were commonly discovered and identified by targeted studies (Hyde 1992a–c; Hyde et al. 1999; Zhou and Hyde 2001; Loilong et al. 2012; Plemenitaš et al. 2014; Suryanarayanan and Ravishankar 2023). On the other hand, these generalists likely reflect high dispersal ability, ecological plasticity, and an inclination for opportunistic infection or colonization (Gilbert et al. 2007; Brown et al. 2012; Nicolaisen et al. 2017; Bebber and Chaloner 2022). Many *Colletotrichum*, *Diaporthe*, and *Exserohilum* species are known to infect diverse, taxonomically distant hosts and to thrive in both agricultural and specialized environments (Sharma et al. 2014; Newman et al. 2020; Dentika et al. 2023; Talhinhas and Baroncelli 2023; Pereira et al. 2023; Balendres et al. 2025; Jayawardena et al. 2025). Fungal surveys on *N. fruticans* often focused on decaying plant parts, resulting in several morphologically described host-associated saprobes, whereas many foliar pathogens and generalists are documented through disease reports, and broad host surveys and pathogenicity assays. However, there are fungal species that have only been reported once and are understudied, *e.g*., *Bacusphaeria nypae* and *Torulaspora nypae*. Thus, further research on these fungi is warranted to support this relationship.

This study presents one of the first comprehensive explorations of microfungi associated with leaf disease on *N. fruticans* using polyphasic identification methods. While the direct effect on *N. fruticans* is still unquantified, foliar diseases hasten leaf senescence (Sinclair and Hudler 1988; Zhao et al. 2022) and have the potential to reduce material quality for *N. fruticans* utilization. Although pathogenicity testing on *N. fruticans* leaves would be challenging due to host material acquisition constraints, it remains essential. Thus, tests on these identified and characterized fungal species are recommended in future studies to confirm their pathogenicity on *N. fruticans*. Cross-infection tests are also encouraged to determine their host range and virulence on other mangrove species and adjacent hosts. Moreover, pathogenicity tests on the reported saprobic and endophytic fungi in *N. fruticans* are recommended to determine whether any of these taxa can adopt pathogenic lifestyles.

Overall, the study revealed previously unidentified and characterized fungal species associated with *N. fruticans* leaf disease in Thailand and emphasizes the mangrove phyllosphere as an underexplored reservoir of taxonomic novelty and host records. Further collection and study of diseases on *N. fruticans* in mangrove hotspots are recommended to present a holistic overview of the disease-associated microfungi of *N. fruticans*. This serves as a taxonomic reference that will facilitate ecological, phytopathological, and conservation-focused studies on *N. fruticans*-associated fungi.

## Supplementary Material

XML Treatment for Brunswickiella nypae

XML Treatment for Pestalotiopsis nypae

XML Treatment for Pestalotiopsis adusta

XML Treatment for Pestalotiopsis spatholobi

XML Treatment for Pestalotiopsis hainanensis

XML Treatment for Colletotrichum siamense

XML Treatment for Diaporthe arecae

## Additional information

### Use of AI

No use of AI was reported.

### Author ORCIDs

Herbert Dustin R. Aumentado https://orcid.org/0000-0003-2888-2669

Ruvishika S. Jayawardena https://orcid.org/0000-0001-7702-4885

Carlo Chris Apurillo https://orcid.org/0000-0003-4348-0887

Chayanard Phukhamsakda https://orcid.org/0000-0002-1033-937X

E. B. Gareth Jones https://orcid.org/0000-0002-7286-5471

Eric H. C. McKenzie https://orcid.org/0000-0001-9400-7112

Kevin D. Hyde https://orcid.org/0000-0002-2191-0762

Yong Wang https://orcid.org/0000-0003-3831-2117

### Data availability

Publicly available datasets were analyzed in this study. These data can be found here: GenBank: https://www.ncbi.nlm.nih.gov. Newly generated sequences and previously published taxa used in this study are presented in Suppl. material 1: tables S1–S4. The estimated nucleotide-substitution model parameters and tree statistics generated in the phylogenetic analyses in this study are presented in Suppl. material 1: table S5. The worldwide checklist of microfungi reported on Nypa fruticans is presented in Suppl. material 2: table S6. Single-gene phylogenetic trees of the novel species are available in Suppl. material 3: figs S1–S6.

## Supplementary materials

Supplementary material 1 GenBank accession numbers used in the phylogenetic analyses in this study, and the estimated nucleotide-substitution model parameters and tree statistics generated in the phylogenetic analyses in this study Data type docx Explanation note **table SS1**. Genera within Mycosphaerellaceae with their respective GenBank accession numbers used in the phylogenetic analyses in this study. **table SS2**. *Pestalotiopsis* with their respective GenBank accession numbers used in the phylogenetic analyses in this study. **table S3**. *Colletotrichum gloeosporioides* species complex with their respective GenBank accession numbers used in the phylogenetic analyses in this study. **table S4**. *Diaporthe* section *Foeniculina* with their respective GenBank accession numbers used in the phylogenetic analyses in this study. **table S5**. Estimated nucleotide-substitution model parameters and tree statistics generated in the phylogenetic analyses in this study. https://binary.pensoft.net/file/1582329This dataset is made available under the Open Database License (http://opendatacommons.org/licenses/odbl/1.0/). The Open Database License (ODbL) is a license agreement intended to allow users to freely share, modify, and use this Dataset while maintaining this same freedom for others, provided that the original source and author(s) are credited. Herbert Dustin R. Aumentado, Ruvishika S. Jayawardena, Carlo Chris Apurillo, Chayanard Phukhamsakda, E. B. Gareth Jones, Eric H. C. McKenzie, Kevin D. Hyde, Long-Feng Yu, Yong Wang

Supplementary material 2 Worldwide checklist of microfungi reported on *Nypa fruticans*, their distribution and lifestyle/s Data type docx https://binary.pensoft.net/file/1582330This dataset is made available under the Open Database License (http://opendatacommons.org/licenses/odbl/1.0/). The Open Database License (ODbL) is a license agreement intended to allow users to freely share, modify, and use this Dataset while maintaining this same freedom for others, provided that the original source and author(s) are credited. Herbert Dustin R. Aumentado, Ruvishika S. Jayawardena, Carlo Chris Apurillo, Chayanard Phukhamsakda, E. B. Gareth Jones, Eric H. C. McKenzie, Kevin D. Hyde, Long-Feng Yu, Yong Wang

Supplementary material 3 Single-gene phylogenetic trees of the novel species Data type pdf Explanation note **fig. S1**. *Brunswickiella*: ITS. **fig. S2**. *Brunswickiella*: LSU. **fig. S3**. *Brunswickiella*: RPB2. **fig. S4**. *Pestalotiopsis*: ITS. **fig. S5**. *Pestalotiopsis*: TEF1. **fig. S6**. *Pestalotiopsis*: TUB2. https://binary.pensoft.net/file/1582331This dataset is made available under the Open Database License (http://opendatacommons.org/licenses/odbl/1.0/). The Open Database License (ODbL) is a license agreement intended to allow users to freely share, modify, and use this Dataset while maintaining this same freedom for others, provided that the original source and author(s) are credited. Herbert Dustin R. Aumentado, Ruvishika S. Jayawardena, Carlo Chris Apurillo, Chayanard Phukhamsakda, E. B. Gareth Jones, Eric H. C. McKenzie, Kevin D. Hyde, Long-Feng Yu, Yong Wang
