# Supplementary material for: Putative palm pathogens: Novel species and new host records of leaf disease-associated microfungi (Ascomycota) on Nypa fruticans in Thailand
Source: MycoKeys. 2026 Apr 6;130:315–53. doi: 10.3897/mycokeys.130.175665 (PMC13077315; doi:10.3897/mycokeys.130.175665)
Supplement: Supplementary material 1 — GenBank accession numbers used in the phylogenetic analyses in this study, and the estimated nucleotide-substitution model parameters and tree statistics generated in the phylogenetic analyses in this study [file mycokeys-130-315-s001.docx]

**Table S1.** Genera within *Mycosphaerellaceae* with their respective GenBank accession numbers used in the phylogenetic analyses in this study.

| **Taxa** | **Strain** | **LSU** | **ITS** | ***RPB2*** |
| --- | --- | --- | --- | --- |
| ***Brunneosphaerella jonkershoekensis*** | **CPC 13902 ^T^** | **NG_058654** | **NR_156244** | **MF951441** |
| *Brunneosphaerella protearum* | CBS 130597 | MH877280 | MH865854 | MF951443 |
| ***Brunswickiella parsonsiae*** | **CBS 137979 ^T^** | **KJ869188** | **KJ869131** | **MF951593** |
| ***Brunswickiella nypae*** | **MFLU25-0393 ^T^** | **PX649205** | **PX612243** | **PX692517** |
| *Brunswickiella nypae* | MFLU25-0394 | **PX649206** | **PX612244** | **PX692518** |
| *Brunswickiella nypae* | MFLU25-0395 | **PX649207** | **PX612245** | **PX692519** |
| ***Cytostagonospora martiniana*** | **CBS 135102 ^ET^** | **KF251657** | **KF251153** | **MF951484** |
| *Devonomyces endophyticus* | CBS 110501 | EU167580 | EU167580 | MF951589 |
| *Devonomyces endophyticus* | CBS 111167 | KF902058 | KF901711 | MF951588 |
| ***Devonomyces endophyticus*** | **CBS 114662 ^T^** | **KF902060** | **KF901713** | **MF951590** |
| *Devonomyces endophyticus* | CBS 114709 | EU167585 | EU167585 | MF951591 |
| *Devonomyces endophyticus* | CPC 15580 | MF951212 | MF951357 | MF951592 |
| ***Hippopotamyces phragmitis*** | **CPC 36385 ^T^** | **NG_068325** | **NR_166324** | **MN556803** |
| ***Hyalozasmidium aerohyalinosporum*** | **CBS 125011 ^T^** | **NG_059440** | **NR_156220** | **MF951504** |
| ***Hyalozasmidium sideroxyli*** | **CBS 142191 ^T^** | **NG_058489** | **NR_156372** | **MF951505** |
| *Lecanosticta acicola* | CBS 871.95 | GU214663 | GU214663 | MF951506 |
| ***Lecanosticta acicola*** | **CBS133791 ^ET^** | **KC013017** | **KC012999** | **MF951507** |
| ***Lecanosticta brevispora*** | **CBS133601 ^T^** | **KF902021** | **JX901763** | **MF951508** |
| ***Lecanosticta longispora*** | **CBS133602 ^ET^** | **JX901858** | **JX901766** | **MF951510** |
| *Lecanosticta longispora* | CPC 17941 | KF902022 | JX901766 | MF951509 |
| ***Madagascaromyces cannae*** | **ZHKUCC 22-0038 ^T^** | **NG_228945** | **NR_185710** | **ON204605** |
| *Madagascaromyces cannae* | ZHKUCC 22-0039 | ON188674 | ON188676 | ON204606 |
| *Madagascaromyces intermedius* | CBS 124154 | KF902014 | KF901672 | KF902268 |
| ***Neomycosphaerella pseudopentameridis*** | **CBS 136407 ^T^** | **NG_058044** | **NR_137832** | **MF951545** |
| ***Paramycosphaerella brachystegiae*** | **CBS 136436 ^T^** | **NG_058048** | **NR_137835** | **MF951567** |
| *Paramycosphaerella pterocarpi* | CBC 147071=CPC 39035 **^T^** | MZ064506 | NR_173059 | MZ078208 |
| ***Paramycosphaerella watsoniae*** | **CBS 146064 = CPC 37392 ^T^** | **NG_068339** | **NR_166341** | **MN556814** |
| *Phaeophleospora eugeniae* | CBS142184=CPC15143 | FJ493206 | FJ493188 | MF951594 |
| *Phaeophleospora eugeniae* | CPC 15159 | FJ493207 | FJ493189 | MF951595 |
| ***Pseudopericoniella levispora*** | **CBS 873.73 ^T^** | **NG_057778** | **NR_137687** | **MF951633** |
| ***Xenosonderhenia eucalypti*** | **CBS 138858 ^T^** | **NG_058120** | **NR_137937** | **MF951688** |
| ***Xenosonderhenia syzygii*** | **CPC 19790 ^T^** | **NG_042685** | **NR_111763** |  |
| ***Zasmidium grevilleae*** | **CBS 124107** | **FJ839670** | **FJ839634** | **MF951705** |

**Table S2.** *Pestalotiopsis* with their respective GenBank accession numbers used in the phylogenetic analyses in this study.

| **Species** | **Strain** | **ITS** | ***tub2*** | ***tef-1a*** |
| --- | --- | --- | --- | --- |
| ***Pestalotiopsis abietis*** | **CFCC 53011 ^T^** | **MK397013** | **MK622280** | **MK622277** |
| *P. abietis* | CFCC 53012 | MK397014 | *MK622281* | *MK622278* |
| *P. abietis* | CFCC 53013 | MK397015 | *MK622282* | *MK622279* |
| ***P. adusta*** | **ICMP 6088 ^T^** | **JX399006** | **JX399070** | **JX399037** |
| *P. adusta* | MFLUCC10-0146 | JX399007 | JX399038 | JX399071 |
| *P. adusta* (*P. mangifericola*) | HJAUP C1639.221 ^T^ | PP962272 | PP952217 | PP952251 |
| *P. adusta* (*P. mangifericola*) | HJAUP C1639.222 | PP962273 | PP952218 | PP952250 |
| *P. adusta* (*P. papuana*) | CBS 887.96 | KM199318 | KM199415 | KM199492 |
| *P. adusta* (*P. papuana*) | CBS 331.96 ^T^ | KM199321 | KM199413 | KM199491 |
| ***P. aggestorum*** | **LC6301 ^T^** | **KX895015** | **KX895348** | **KX895234** |
| *P. aggestorum* | LC8186 | KY464140 | KY464160 | KY464150 |
| *P. alloschemones* | CGMCC 3.23480 | OR247981 | OR381056 | OR361456 |
| *P. alloschemones* | LC15841 | OR247982 | OR381057 | OR361457 |
| ***P. alpinicola*** | **HJAUP C1644.221 ^T^** | **PP962274** | **PP952219** | **PP952248** |
| *P. alpinicola* | HJAUP C1644.222 | PP962275 | PP952220 | PP952249 |
| *P. americana* | CBS 111576 | MH553961 | MH554620 | MH554379 |
| ***P. anacardiacearum*** | **IFRDCC 2397 ^T^** | **KC247154** | **KC247155** | **KC247156** |
| *P. anhuiensis* | CFCC 54791 | ON007028 | ON005056 | ON005045 |
| ***P. aporosae-dioicae*** | **SAUCC 224004 ^T^** | **OR733506** | **OR912985** | **OR912988** |
| *P. aporosae-dioicae* | SAUCC 224005 | OR733505 | OR912986 | OR912989 |
| ***P. arceuthobii*** | **CBS 434.65 ^T^** | **KM199341** | **KM199427** | **KM199516** |
| ***P. arengae*** | **CBS 331.92 ^T^** | **KM199340** | **KM199426** | **KM199515** |
| *P. australasiae* | CBS 114141 | KM199298 | KM199410 | KM199501 |
| ***P. australasiae*** | **CBS 114126 ^T^** | **KM199297** | **KM199409** | **KM199499** |
| *P. australis* | CBS 111503 | KM199331 | KM199382 | KM199557 |
| ***P. australis*** | **CBS 114193 ^T^** | **KM199332** | **KM199383** | **KM199475** |
| *P. biappendiculata* | CGMCC 3.23487 | OR247984 | OR381059 | OR361459 |
| *P. biappendiculata* | LC4282 | OR247990 | OR381065 | OR361465 |
| ***P. biciliata*** | **CBS 124463 ^T^** | **KM199308** | **KM199399** | **KM199505** |
| *P. biciliata* | CBS 236.38 | KM199309 | KM199401 | KM199506 |
| *P. biciliata* | CBS 790.68 | KM199305 | KM199400 | KM199507 |
| ***P. brachiata*** | **LC2988 ^T^** | **KX894933** | **KX895265** | **KX895150** |
| *P. brachiata* | LC8188 | KY464142 | KY464162 | KY464152 |
| ***P. brassicae*** | **CBS 170.26 ^T^** | **KM199379** |  | **KM199558** |
| *P. brassicae* (*P. hollandica*) | CBS 265.33 ^T^ | KM199328 | KM199388 | KM199481 |
| ***P. buxicola*** | **CFCC 57357 ^T^** | **PP965518** | **PP957901** | **PP957899** |
| P. buxicola | CFCC 57358 | PP965519 | PP957902 | PP957900 |
| ***P. camelliae*** | **MFLUCC 12-0277 ^T^** | **JX399010** | **JX399041** | **JX399074** |
| *P. camelliae* | CBS 443.62 | KM199336 | KM199424 | KM199512 |
| *P. camelliae* (*P. yanglingensis*) | LC3067 | KX894949 | KX895281 | KX895166 |
| *P. camelliae* (*P. yanglingensis*) | LC4553 ^T^ | KX895012 | KX895345 | KX895231 |
| ***P. camelliae-japonicae*** | **ZHKUCC 23-0826 ^T^** | **OR258040** | **OR251483** | **OR251480** |
| *P. camelliae-japonicae* | ZHKUCC 23-0827 | OR258041 | OR251484 | OR251481 |
| ***P. camelliicola*** | **HJAUP C1804.221 ^T^** | **PP962357** | **PP952229** | **PP952236** |
| *P. cangshanensis* | CGMCC 3.23544 | OP082426 | OP185517 | OP185510 |
| *P. cangshanensis* | CFCC 54430 | OK339732 | OK358508 | OK358493 |
| *P. castanopsidis* | CFCC 54430 | OK339732 | OK358508 | OK358493 |
| *P. castanopsidis* | CFCC 54384 | OK339734 | OK358510 | OK358495 |
| *P. changjiangensis* | CFCC 54314 | OK339739 | OK358515 | OK358500 |
| *P. changjiangensis* | CFCC 52803 | OK339741 | OK358517 | OK358502 |
| *P. chaoyangensis* | CFCC 55549 | OQ344763 | OQ410584 | OQ410582 |
| *P. chaoyangensis* | CFCC 58805 | OQ344764 | OQ410585 | OQ410583 |
| *P. chiangmaiensis* | MFLUCC 22-0127 | OP497990 | OP752137 | OP753374 |
| *P. chiaroscuro* | BRIP 72970a | OK422510 | OK423752 | OK423753 |
| *P. chinensis* | MFLUCC 12-0273 | JX398995 |  |  |
| ***P. clavata*** | **MFLUCC 12-0268 ^T^** | **JX398990** | **JX399025** | **JX399056** |
| ***P. colombiensis*** | **CBS 118553 ^T^** | **KM199307** | **KM199421** | **KM199488** |
| ***P. cyclobalanopsidis*** | **CFCC 54328 ^T^** | **OK339735** | **OK358511** | **OK358496** |
| *P. cyclobalanopsidis* | CFCC 55891 | OK339736 | OK358512 | OK358497 |
| ***P. cyclosori*** | **HJAUP C1724.221 ^T^** | **PP962279** | **PP952221** | **PP952247** |
| *P. cyclosori* | HJAUP C1724.222 | PP962280 | PP952222 | PP952246 |
| *P. cyclosori* | HJAUP C1725.221 | PP962281 | PP952223 | PP952245 |
| *P. cyclosori* | HJAUP C1725.222 | PP962282 | PP952224 | PP952244 |
| *P. cyclosori* | HJAUP C1726.221 | PP962283 | PP952232 | PP952243 |
| *P. cyclosori* | HJAUP C1726.222 | PP962284 | PP952233 | PP952242 |
| *P. dianellae* | CBS 143421 | MG386051 | MG386164 |  |
| ***P. digitalis*** | **ICMP 5434 ^T^** | **KP781879** | **KP781883** |  |
| *P. diploclisiae* | CBS 115449 | KM199314 | KM199416 | KM199485 |
| *P. diploclisiae* | CBS 115585 | KM199315 | KM199417 | KM199483 |
| ***P. diploclisiae*** | **CBS 115587 ^T^** | **KM199320** | **KM199419** | **KM199486** |
| *P. disseminata* | CBS 118552 | MH553986 | MH554652 | MH554410 |
| *P. disseminata* | CBS 143904 | MH554152 | MH554825 | MH554587 |
| *P. disseminata* | CPC 29351 | MH554166 | MH554839 | MH554601 |
| ***P. distincta*** (*P. dilucida*) | **LC3232 ^T^** | **KX894961** | **KX895293** | **KX895178** |
| *P. distincta* (*P. dilucida*) | LC8184 | KY464138 | KY464158 | KY464148 |
| ***P. diversiseta*** | **MFLUCC 12-0287 ^T^** | **NR_120187** | **JX399040** | **JX399073** |
| ***P. doitungensis*** | **MFLUCC 14-0090 ^T^** | **MK993573** | **MK975836** | **MK975831** |
| ***P. dracaenae*** | **HGUP4037 ^T^** | **MT596515** | **MT598645** | **MT598644** |
| ***P. dracaenicola*** | **MFLUCC 18-0913 ^T^** | **MN962731** | **MN962733** | **MN962732** |
| *P. dracaenicola* | MFLUCC 18-0914 | MN962734 | MN962736 | MN962735 |
| *P. dracontomelonis* | MFLUCC 22-0145 | OP802395 | OP801772 | OP830894 |
| *P. eleutherococci* | HMJAU 60189 | OL996126 |  |  |
| ***P. endophytica*** | **MFLUCC 18-0932 ^T^** | **PQ129358** | **PQ148943** | **PQ140993** |
| ***P. eriobotryae*** | **HJAUP C1742.221 ^T^** | **PP962289** | **PP952227** | **PP952238** |
| *P. eriobotryae* | HJAUP C1742.222 | PP962291 | PP952228 | PP952237 |
| ***P. ericacearum*** | **IFRDCC 2439 ^T^** | **KC537807** | **KC537821** | **KC537814** |
| ***P. etonensis*** | **BRIP 66615^T^** | **MK966339** | **MK977634** | **MK977635** |
| ***P. exudata*** | **CGMCC 3.23488 ^T^** | **OR247985** | **OR381060** | **OR361460** |
| *P. exudata* | LC15850 | OR247986 | OR381061 | OR361461 |
| *P. ficicrescens* | GUCC 21556 | MZ477311 | MZ868301 | MZ868328 |
| ***P. ficicrescens*** | **CGMCC 3.23471 ^T^** | **OR247980** | **OR381055** | **OR361455** |
| *P. ficicrescens* | LC2960 | OR247983 | OR381058 | OR361458 |
| ***P. foliicola*** | **CFCC 54440 ^T^** | **ON007029** | **ON005057** | **ON005046** |
| *P. foliicola* | CFCC 57359 | ON007030 | ON005058 | ON005047 |
| *P. foliicola* | CFCC 57360 | ON007031 | ON005059 | ON005048 |
| ***P. formosana*** | **NTUCC 17-009 ^T^** | **MH809381** | **MH809385** | **MH809389** |
| *P. formosana* | NTUCC 17-010 | MH809382 | MH809386 | MH809390 |
| ***P. furcata*** | **MFLUCC 12-0054 ^T^** | **JQ683724** | **JQ683708** | **JQ683740** |
| *P. furcata* | LC6303 | KX895016 | KX895349 | KX895235 |
| ***P. fusiformis*** | **CGMCC 3.23495 ^T^** | **OR247995** | **OR381070** | **OR361470** |
| *P. fusiformis* | LC15852 | OR247996 | OR381071 | OR361471 |
| ***P. fusoidea*** | **CGMCC 3.23545 ^T^** | **OP082427** | **OP185519** | **OP185512** |
| ***P. ganzhouensis*** | **CGMCC 3.23489 ^T^** | **OR247987** | **OR381062** | **OR361462** |
| *P. ganzhouensis* | LC5089 | OR247998 | OR381073 | OR361473 |
| ***P. gardeniae*** | **HJAUP C1729.221 ^T^** | **PP962285** | **PP952225** | **PP952241** |
| *P. gardeniae* | HJAUP C1729.222 | PP962286 | PP952226 | PP952240 |
| ***P. gaultheriae*** | **IFRD 411-014 ^T^** | **KC537805** | **KC537819** | **KC537812** |
| ***P. gibbosa*** | **NOF 3175 ^T^** | **LC311589** | **LC311590** | **LC311591** |
| ***P. guangdongensis*** | **ZHKUCC 22–0016 ^T^** | **ON180762** | **ON221548** | **ON221520** |
| *P. guangdongensis* | ZHKUCC 22–0017 | ON180763 | ON221549 | ON221521 |
| ***P. guangxiensis*** | **CFCC 54300 ^T^** | **OK339738** | **OK358514** | **OK358499** |
| *P. guangxiensis* | CFCC 54308 | OK339737 | OK358513 | OK358498 |
| ***P. guiyangensis*** | **CFCC 70626 ^T^** | **PP784740** | **PP842617** | **PP842629** |
| *P. guiyangensis* | CFCC 70630 | PP784741 | PP842618 | PP842630 |
| ***P. guizhouensis*** | **CFCC 54803 ^T^** | **ON007035** | **ON005063** | **ON005052** |
| *P. guizhouensis* | CFCC 57364 | ON007036 | ON005064 | ON005053 |
| ***P. grevilleae*** | **CBS 114127 ^T^** | **KM199300** | **KM199407** | **KM199504** |
| ***P. hainanensis*** | **PSHI2004Endo166 ^T^** | **DQ334863** | **DQ137861** |  |
| *P. hainanensis* (*P. appendiculata*) | CGMCC 3.23550 | OP082431 | OP185516 | OP185516 |
| *P. hainanensis* (*P. chamaeropis*) | CBS 113607 | KM199325 | KM199390 | KM199472 |
| *P. hainanensis* (*P. chamaeropis*) | CBS 186.71 ^T^ | KM199326 | KM199391 | KM199473 |
| *P. hainanensis* (*P. daliensis*) | CGMCC 3.23548 | OP082429 | OP185518 | OP185511 |
| *P. hainanensis* (*P. intermedia*) | MFLUCC 12-0259 ^T^ | JX398993 | JX399028 | JX399059 |
| *P. hainanensis* (*P. linearis*) | MFLUCC12-0272 | JX398994 |  | JX399060 |
| *P. hainanensis* (*P. machiliana*) | HJAUP C1704.221 ^T^ | PP962276 | PP952211 | PP952255 |
| *P. hainanensis* (*P. machiliana*) | HJAUP C1704.222 | PP962277 | PP952212 | PP952256 |
| *P. hainanensis* (*P. machiliana*) | HJAUP C1704.223 | PP962278 | PP952213 | PP952257 |
| *P. hainanensis* (*P. neohantoniensis*) | C5 | PQ568303 | PQ573016 | PQ573021 |
| *P. hainanensis* (*P. neohantoniensis*) | C9 | PQ568306 | PQ573019 | PQ573024 |
| *P. hainanensis* (*P. neohantoniensis*) | C10 | PQ568307 | PQ573020 | PQ573025 |
| *P. hainanensis* (*P. rosarioides*) | CGMCC3.23549 ^T^ | OP082430 | OP185520 | OP185513 |
| *P. hainanensis* (*P. schisandrae*) | CFCC 59550 ^T^ | OR775411 | OR766014 | OR766002 |
| *P. hainanensis* (*P. schisandrae*) | CFCC 59551 | OR775412 | OR766015 | OR766003 |
| *P. hainanensis* (*P. tumida*) | CGMCC 3.23502 ^T^ | OR247999 | OR381074 | OR361474 |
| *P. hainanensis* (*P. tumida*) | CFCC 55158 | OK560610 | OM158174 | OL814524 |
| *P. hainanensis* (*P. tumida*) | CFCC 55159 | OK560613 | OM158177 | OL814527 |
| ***P. hawaiiensis*** | **CBS 114491 ^T^** | **KM199514** | **KM199428** | **KM199514** |
| ***P. hederae*** | **HJAUP C1638.221 ^T^** | **PP962270** | **PP952234** | **PP952252** |
| *P. hederae* | HJAUP C1638.222 | PP962271 | PP952216 |  |
| ***P. hispanica*** | **CBS 115391 ^T^** | **MH553981** | **MH554640** | **MH554399** |
| ***P. humicola*** | **CBS 336.97 ^T^** | **KM199317** | **KM199420** | **KM199484** |
| ***P. hunanensis*** | **CSUFTCC15 ^T^** | OK493599 | OK562374 | OK507969 |
| *P. hunanensis* | CSUFTCC18 | OK493600 | OK562375 | OK507970 |
| *P. hunanensis* (*P. jiangsuensis*) | CFCC 59538 ^T^ | OR533577 | OR539191 | OR539186 |
| ***P. hydei*** | **MFLUCC 20-0135 ^T^** | **NR_172003** | **MW251112** | **MW251113** |
| *P. hydei* | E-72-02 | KU926708 | KU926716 | KU926712 |
| *P. hydei* | E-72-03 | KU926709 | KU926717 | KU926713 |
| ***P. inflexa*** | **MFLUCC 12-0270 ^T^** | **JX399008** | **JX399039** | **JX399072** |
| ***P. italiana*** | **MFLU 14-0214 ^T^** | **KP781878** | **KP781882** | **KP781881** |
| *P. jesteri* | MFLUCC 12-0279 | JX399012 | JX399043 | JX399076 |
| ***P. jinchanghensis*** | **LC6636 ^T^** | **KX895028** | **KX895361** | **KX895247** |
| *P. jinchanghensis* | LC8190 | KY464144 | KY464164 | KY464154 |
| ***P. kaki*** | **KNU-PT-1804 ^T^** | **LC552953** | **LC552954** | **LC553555** |
| *P. kandelicola* | NCYUCC 19-0355 | MT560722 | MT563099 | MT563101 |
| ***P. kandelicola*** | **NCYUCC 19-0354 ^T^** | **MT560723** | **MT563100** | **MT563102** |
| ***P. knightiae*** | **CBS 114138 ^T^** | **KM199310** | **KM199408** | **KM199497** |
| *P. knightiae* | CBS 111963 | KM199311 | KM199406 | KM199495 |
| ***P. krabiensis*** | **MFLUCC 16-0260 ^T^** | **MH388360** | **MH412722** | **MH388395** |
| ***P. leucadendri*** | **CBS 121417 ^T^** | **MH553987** | **MH554654** | **MH554412** |
| *P. leucospermi* | CBS 114489 | MH553978 | MH554396 | MH554637 |
| ***P. licualacola*** | **HGUP 4057 ^T^** | **KC492509** | **KC481683** | **KC481684** |
| ***P. lithocarpi*** | **CFCC 55893 ^T^** | **OK339743** | **OK358519** | **OK358504** |
| *P. lithocarpi* | CFCC 55100 | OK339742 | OK358518 | OK358503 |
| *P. lobata* | CGMCC 3.23467 | OR247976 | OR381051 | OR361451 |
| *P. lobata* | LLC15843 | OR247977 | OR381052 | OR361452 |
| ***P. loeiana*** | **MFLUCC 22-0123 ^T^** | **OP497988** | **OP713769** | **OP737881** |
| ***P. lushanensis*** | **LC4344 ^T^** | **KX895005** | **KX895337** | **KX895223** |
| *P. lushanensis* | LC8182 | KY464136 | KY464156 | KY464146 |
| *P. lushanensis* (*P. iberica*) | MUM-H 21.02 ^T^ | NR_177558 | MW759035 | MW759038 |
| *P. lushanensis* (*P. iberica*) | CAA 1005 | MW732250 | MW759034 | MW759037 |
| ***P. macadamiae*** | **BRIP 63738b ^T^** | **KX186588** | **KX186680** | **KX186621** |
| ***P. machili*** | **CGMCC 3.23511 ^T^** | **OR248003** | **OR381078** | **OR361478** |
| *P. maineana* | C7 | PQ568304 | PQ573017 | PQ573022 |
| *P. maineana* | C8 | PQ568305 | PQ573018 | PQ573023 |
| ***P. malayana*** | **CBS 102220 ^T^** | **KM199306** | **KM199411** | **KM199482** |
| *P. manyueyuanensis* | NTUPPMCC 18-165 | OR125060 | OR126306 | OR126313 |
| *P. manyueyuanensis* | NTUPPMCC 22-012 | OR125061 | OR126307 | OR126314 |
| ***P. massoniana*** | **ZXD955 = CFCC 72593 ^T^** | **PV259820** | **PV275215** | **PV275141** |
| *P. massoniana* | ZXD956 = CFCC 72594 | PV259821 | PV275216 | PV275142 |
| ***P. menhaiensis*** | **YN3A 1 = CGMCC 3.18250 ^T^** | **KU252272** | **KU252488** | **KU252401** |
| *P. menhaiensis* | YN3A 2 | KX146685 | KX146803 | KX146744 |
| ***P. monochaeta*** | **CBS 144.97 ^T^** | **KM199327** | **KM199386** | **KM199479** |
| *P. monochaeta* | CBS 440.83 | KM199329 | KM199387 | KM199480 |
| ***P. montellica*** | **MFLUCC 12-0279 ^T^** | **JX399012** | **JX399043** | **JX399076** |
| ***P. multiappendiculata*** | **CGMCC 3.23514^T^** | **OR248008** | **OR381083** | **OR361483** |
| *P. multicolor* | CFCC59981 | OQ626676 | OQ714336 | OQ714341 |
| *P. multicolor* | CFCC59982 | OQ771896 | OQ779488 | OQ779483 |
| ***P. nanjingensis*** | **CFCC 53882 ^T^** | **OM746295** | **OM839968** | **OM840067** |
| ***P. nanningensis*** | **CSUFTCC10 ^T^** | **OK493596** | **OK562371** | **OK507966** |
| *P. nanningensis* | CSUFTCC11 | OK493597 | OK562372 | OK507967 |
| ***P. nannuoensis*** | **SAUCC 232203 ^T^** | **OR733504** | **OR863909** | **OR912991** |
| *P. nannuoensis* | SAUCC 232204 | OR733503 | OR863910 | OR912992 |
| *P. neglecta* | NEFU016 |  | MN176187 | MK775464 |
| ***P. neolitseae*** | **NTUCC 17-011 ^T^** | **MH809383** | **MH809387** | **MH809391** |
| *P. neolitseae* | NTUCC 17-012 | MH809384 | MH809388 | MH809392 |
| *P. neolitseae* | ZL11 | OL546622 | OL598347 | OL598346 |
| ***P. ningboensis*** | **ZXD39 = CFCC 72585 ^T^** | **PV259822** | **PV275217** | **PV275143** |
| *P. ningboensis* | ZXD40 = CFCC 72586 | PV259823 | PV275218 | PV275144 |
| ***P. novae-hollandiae*** | **CBS 130973 ^T^** | **KM199337** | **KM199425** | **KM199511** |
| ***Pestalotiopsis nypae*** | **MFLU25-0396 ^T^** | **PX612246** | **PX939452** | **PX939448** |
| *P. nypae* | MFLU25-0397 | PX612247 | PX939453 | PX939449 |
| *P. nypae* | MFLU25-0398 | PX612248 | PX939454 | PX939450 |
| *P. nypae* | MFLU25-0399 | PX612249 | PX939455 | PX939451 |
| *P. olivacea* | SY17A | EF055215 | EF055251 |  |
| *P. oryzae* | CBS 111522 | KM199294 | KM199394 | KM199493 |
| ***P. oryzae*** | **CBS 353.69 ^T^** | **KM199299** | **KM199398** | **KM199496** |
| ***P. pallidotheae*** | **MAFF 240993 ^T^** | **NR_111022** | **LC311584** | **LC311585** |
| ***P. pandanicola*** | **MFLUCC 16-0255 ^T^** | **MH388361** | **MH412723** | **MH388396** |
| ***P. parva*** | **CBS 265.37 ^T^** | **KM199312** | **KM199404** | **KM199508** |
| *P. parva* | CBS 278.35 | KM199313 | KM199405 | KM199509 |
| *P. photiniae* | PSHI2002Endo403 | AY682942 | DQ333583 | DQ657877 |
| ***P. photinicola*** | **GZCC 16-0028 ^T^** | **KY092404** | **KY047663** | **KY047662** |
| *P. phyllostachydis* | HNZW177 | OR343210 | OR367676 | OR367675 |
| *P. phyllostachydis* (*P. cratoxyli*) | CGMCC 3.23512 ^T^ | OR248005 | OR381080 | OR361480 |
| *P. phyllostachydis* (*P. cratoxyli*) | LC8780 | OR248006 | OR381081 | OR361481 |
| *P. pini* | MEAN 1092 | MT374680 | MT374705 | MT374693 |
| *P. pini* | MEAN 1167 | MT374689 | MT374714 | MT374701 |
| ***P. pinicola*** | **KUMCC 19-0183 ^T^** | **MN412636** | **MN417507** | **MN417509** |
| *P. pinicola* | KUMCC 19-0203 | MN412637 | MN417508 | MN417510 |
| *P. piraubensis* | COAD 2165 | MH627381 | MH643773 | MH643774 |
| ***P. portugalica*** | **CBS 393.48 ^T^** | **KM199335** | **KM199422** | **KM199510** |
| *P. portugalica* | LC2929 | KX894921 | KX895253 | KX895138 |
| ***P. pruni*** | **CGMCC 3.23507 ^T^** | **OR248001** | **OR381076** | **OR361476** |
| *P. pruni* | LC15860 | OR248002 | OR381077 | OR361477 |
| ***P. rhaphiolepidis*** | **SAUCC 367701 ^T^** | **OR733502** | **OR863906** | **OR912994** |
| *P. rhaphiolepidis* | SAUCC 367702 | OR733501 | OR863907 | OR912995 |
| ***P. rhizophorae*** | **MFLUCC 17-0416 ^T^** | **MK764283** | **MK764349** | **MK764327** |
| *P. rhizophorae* | MFLUCC 17-0417 | MK764284 | MK764350 | MK764328 |
| *P. rhodomyrtus* | LC3413 | KX894981 | KX895313 | KX895198 |
| *P. rhodomyrtus* | LC4458 | KX895010 | KX895342 | KX895228 |
| ***P. rosea*** | **MFLUCC12-0258 ^T^** | **JX399005** | **JX399036** | **JX399069** |
| ***P. rubrae*** | **CGMCC 3.23499^T^** | **OR247997** | **OR381072** | **OR361472** |
| *P. rubrae* | LC8233 | OR248000 | OR381075 | OR361475 |
| ***P. sabal*** | **ZHKUCC 22–0035 ^T^** | **ON180775** | **ON221561** | **ON221533** |
| ***P. scoparia*** | **CBS 176.25 ^T^** | **KM199330** | **KM199393** | **KM199478** |
| ***P. sequoiae*** | **MFLUCC 13-0399 ^T^** | **KX572339** |  |  |
| ***P. shaanxiensis*** | **CFCC 54958 ^T^** | **ON007026** | **ON005054** | **ON005043** |
| *P. shaanxiensis* | CFCC 57356 | ON007027 | ON005055 | ON005044 |
| *P. shandongensis* | KUMCC 19-0241 | MN625275 | MN626729 | MN626740 |
| ***P. shanweiensis*** | **ZXD950 = CFCC 72591 ^T^** | **PV259824** | **PV275219** | **PV275145** |
| *P. shanweiensis* | ZXD951 = CFCC 72592 | PV259825 | PV275220 | PV275146 |
| ***P. shoreae*** | **MFLUCC 12-0314 ^T^** | **KJ503811** | **KJ503814** | **KJ503817** |
| ***P. sichuanensis*** | **CGMCC 3.18244 ^T^** | **KX146689** | **KX146807** | **KX146748** |
| *P. sichuanensis* | CFCC 53882 | OM746295 | OM839968 | OM840067 |
| *P. sichuanensis* | CSUFTCC16 | OK493602 | OK562377 | OK507972 |
| ***P. silvicola*** | **CFCC 55296 ^T^** | **ON007032** | **ON005060** | **ON005049** |
| *P. silvicola* | CFCC 54915 | ON007033 | ON005061 | ON005050 |
| ***P. smilacicola*** | **MFLUCC 22-0124 ^T^** | **OP497989** | **OP762674** | **OP737879** |
| *P. smilacicola* | MFLUCC 22-0125 | OP497991 | OP762673 | OP753376 |
| ***P. solicola*** | **SAUCC 003804 ^T^** | **OQ692020** | **OQ718795** | **OQ718737** |
| *P. solicola* | SAUCC003806 | OQ692021 | OQ718796 | OQ718738 |
| *P. solicola* | SAUCC003807 | OQ692022 | OQ718797 | OQ718739 |
| ***P. sonneratiae*** | **CFCC 57394 ^T^** | **ON114184** | **ON086816** | **ON086812** |
| *P. sonneratiae* | CFCC 57395 | ON114185 | ON086817 | ON086813 |
| ***P. spatholobi*** | **SAUCC231201 ^T^** | **OQ692023** | **OQ718798** | **OQ718740** |
| *P. spatholobi* | SAUCC231203 | OQ692024 | OQ718799 | OQ718741 |
| *P. spatholobi* (*P. jiangmenensis*) | ZXD964 = CFCC 72595 ^T^ | PV259810 | PV275205 | PV275131 |
| *P. spatholobi* (*P. jiangmenensis*) | ZXD965 = CFCC 72596 | PV259811 | PV275206 | PV275132 |
| *P. spatholobi* (*P. pyrrosiae-linguae*) | ZHKUCC 23-0807 ^T^ | OR199902 | OR259258 | OR259260 |
| *P. spatholobi* (*P. pyrrosiae-linguae*) | ZHKUCC 23-0808 | OR199903 | OR259259 | OR259261 |
| ***P. spathulata*** | **CBS 356.86 ^T^** | **KM199338** | **KM199423** | **KM199513** |
| ***P. spathuliappendiculata*** | **CBS 144035 ^T^** | **MH554172** | **MH554845** | **MH554607** |
| ***P. suae*** | **CGMCC 3.23546 ^T^** | **OP082428** | **OP185521** | **OP185514** |
| *P. telopeae* | CBS 113606 | KM199295 | KM199402 | KM199498 |
| ***P. telopeae*** | **CBS 114161 ^T^** | **KM199296** | **KM199403** | **KM199500** |
| *P. telopeae* | CBS 114137 | KM199301 | KM199469 | KM199559 |
| ***P. terricola*** | **CBS 141.69 ^T^** | **MH554004** | **MH554680** | **MH554438** |
| *P. thailandica* | MFLUCC 17-1617 | MK764285 | MK764351 | MK764329 |
| ***P. thailandica*** | **MFLUCC 17-1616 ^T^** | **MK764285** | **MK764351** | **MK764329** |
| ***P. thunbergii*** | **CFCC 72589 ^T^** | **PV259838** | **PV275233** | **PV275159** |
| ***P. thunbergii*** | CFCC 72590 | PV259839 | PV275234 | PV275160 |
| ***P. thunbergii*** | ZXD581 | PV259837 | PV275232 | PV275158 |
| ***P. trachicarpicola*** | **OP068 ^T^** | **JQ845947** | **JQ845945** | **JQ845946** |
| *P. trachicarpicola* | MFLUCC 12-0263 | JX399000 | JX399031 | JX399064 |
| *P. trachycarpicola* | BJFUCC42 | OQ626674 | OQ714334 | OQ714339 |
| *P. trachycarpicola* | BJFUCC42-2 | OQ771894 | OQ779486 | OQ779481 |
| *P. trachicarpicola* (*P. kenyana*) | CBS 442.67 ^T^ | KM199302 | KM199395 | KM199502 |
| *P. trachicarpicola* (*P. microspora*) | RA1-2 | JN314418 | JN314419 |  |
| ***P. tumida*** | **CGMCC 3.23502 ^T^** | **OR247999** | **OR381074** | **OR361474** |
| *P. tumida* | CFCC 55158 | OK560610 | OM158174 | OL814524 |
| *P. tumida* | CFCC 55159 | OK560613 | OM158177 | OL814527 |
| ***P. unicolor*** | **MFLUCC 12-0276 ^T^** | **JX398999** | **JX399030** |  |
| *P. unicolor* | MFLUCC 12-0275 | JX398998 | JX399029 | JX399063 |
| *P. unicolor* (*P. jiangxiensis*) | LC4399 ^T^ | KX895009 | KX895341 | KX895227 |
| *P. unicolor* (*P. taxicola*) | CFCC59976 | OQ626673 | OQ714333 | OQ714338 |
| *P. unicolor* (*P. taxicola*) | CFCC59978 | OQ771893 | OQ779485 | OQ779480 |
| *P. uvicola* | UCD2465TX | FJ790874 | GU294730 | GU294716 |
| ***P. verruculosa*** | **MFLUCC 12-0274 ^T^** | **JX398996** |  | **JX399061** |
| ***P. wenzhouensis*** | **ZXD64 = CFCC 72587 ^T^** | **PV259840** | **PV275235** | **PV275161** |
| *P. wenzhouensis* | ZXD79 | PV259841 | PV275236 | PV275162 |
| *P. wenzhouensis* | ZXD86 | PV259842 | PV275237 | PV275163 |
| *P. wenzhouensis* | ZXD966 = CFCC 72588 | PV259845 | PV275240 | PV275166 |
| ***P. wulichongensis*** | **CGMCC 3.23469 ^T^** | **OR247978** | **OR381053** | **OR361453** |
| *P. wulichongensis* | LC15846 | OR247979 | OR381054 | OR361454 |
| *P. yunnanensis* | HMAS 96359 | OR230043 |  |  |
| ***P. zhaoqingensis*** | **ZHKUCC 23-0825 ^T^** | **OR233336** | **OR239062** | **OR239061** |
| ***Pseudopestalotiopsis cocos*** | **CBS 272.29^T^** | **KM199378** | **KM199467** | **KM199553** |

**Table S3.** *Colletotrichum* *gloeosporioides* species complex with their respective GenBank accession numbers used in the phylogenetic analyses in this study.

| **Species** | **Strain** | **ITS** | ***GAPDH*** | ***CHS1*** | ***ACT*** | ***TUB2*** |
| --- | --- | --- | --- | --- | --- | --- |
| ***C. aenigma*** | **ICMP 18608 ^T^** | **JX010244** | **JX010044** | **JX009774** | **JX009443** | **JX010389** |
| ***C. aeschynomenes*** | **ICMP 17673 ^T^** | **JX010176** | **JX009930** | **JX009799** | **JX009483** | **JX010392** |
| *C. aeschynomenes* | COL02 |  | MK792457 |  |  | MK792460 |
| ***C. alatae*** | **CBS 304.67 ^T^** | **JX010190** | **JX009990** | **JX009837** | **JX009471** | **JX010383** |
| ***C. alienum*** | **ICMP 12071 ^T^** | **JX010251** | **JX010028** | **JX009882** | **JX009572** | **JX010411** |
| ***C. anhuiense*** | **AG85 ^T^** | **OL772736** | **OL854171** | **OL854157** | **OM100917** | **OL854185** |
| *C. anhuiense* | AG52 | OL772737 | OL854172 | OL854158 | OM100918 | OL854186 |
| ***C. aotearoa*** | **ICMP 18537 ^T^** | **JX010205** | **JX010005** | **JX009853** | **JX009564** | **JX010420** |
| ***C. arecicola*** | **CGMCC 3.19667 ^T^** | **MK914635** | **MK935455** | **MK935541** | **MK935374** | **MK935498** |
| ***C. artocarpicola*** | **MFLUCC 18-1167 ^T^** | **MN415991** | **MN435568** | **MN435569** | **MN435570** | **MN435567** |
| ***C. australianum*** | **VPRI 43075 ^T^** | **MG572138** | **MG572127** | **MW091987** | **MN442109** | **MG572149** |
| ***C. avicenniae*** | **MFLUCC 23-0289 ^T^** | **OR856121** | **OR886390** | **OR886402** | **OR886393** | **OR886396** |
| ***C. camelliae*** | **CGMCC 3.14925 ^T^** | **KJ955081** | **KJ954782** | **MZ799255** | **KJ954363** | **KJ955230** |
| *C. camelliae* (≡*C. analogum*) | YMF 1.06943 | OK030860 | OK513663 | OK513559 | OK513599 | OK513629 |
| ***C. cangyuanensis*** | **CGMCC 3.17582 ^T^** | **OK030864** | **OK513667** | **OK513563** | **OK513603** | **OK513633** |
| *C. cangyuanensis* | YMF1.04998 | OK030865 | OK513668 | OK513564 | OK513604 | OK513634 |
| ***C. castaneae*** | **GUCC 21268.4 ^T^** | **OP722991** | **OP737973** | **OP715778** | **OP715812** | **OP720868** |
| *C. castaneae* | GUCC 12176 | OP722990 | OP737972 | OP715777 | OP715811 | OP720867 |
| ***C. changpingense*** | **MFLUCC 15-0022 ^T^** | **KP683152** | **KP852469** | **KP852449** | **KP683093** | **KP852490** |
| ***C. chiangmaiense*** | **MFLUCC 18-0945 ^T^** | **MW346499** | **MW548592** | **MW623653** | **MW655578** |  |
| ***C. chinensis*** | **CGMCC 3.25209 ^T^** | **OR287154** | **OR295535** | **OR295491** | **OR295513** | **OR295557** |
| *C. chinensis* | CGMCC 3.25210 | OR287156 | OR295536 | OR295492 | OR295514 | OR295558 |
| ***C. chrysophilum*** | **URM7368 ^T^** | **KX094252** | **KX094183** | **KX094083** | **KX093982** | **KX094285** |
| *C. chrysophilum* | A20_F13_004 | OR030319 | OR037286 |  | OR037283 | OR046884 |
| ***C. cigarro*** | **ICMP 18539 ^T^** | **JX010230** | **JX009966** | **JX009800** | **JX009523** | **JX010434** |
| ***C. citrulli*** | **CAASZT52 ^T^** | **MZ475133** | **OL456685** | **OL901153** | **OL449283** | **OL456644** |
| *C. citrulli* | CAASZT54 | MZ475134 | OL456686 | OL901154 | OL449284 | OL456645 |
| ***C. clidemiae*** | **ICMP 18658 ^T^** | **JX010265** | **JX009989** | **JX009877** | **JX009537** | **JX010438** |
| ***C. cobbittiense*** | **BRIP 66219 ^T^** | **MH087016** | **MH094133** | **MH094135** | **MH094134** | **MH094137** |
| ***C. conoides*** | **CGMCC 3.17615 ^T^** | **KP890168** | **KP890162** | **KP890156** | **KP890144** | **KP890174** |
| ***C. cordylinicola*** | **MFLUCC 90551 ^T^** | **JX010226** | **JX009975** | **JX009864** | **JX009586** | **JX010440** |
| *C. cycadis* | BRIP 71326a | MT439915 | MT439919 | MT439917 |  | MT439921 |
| ***C. dracaenigenum*** | **MFLUCC 19-0430T ^T^** | **MN921250** | **MT215577** | **MT215575** | **MT313686** |  |
| ***C. endophyticum*** | **MFLUCC 13-0418 ^T^** | **KC633854** | **KC832854** | **MZ799261** | **KF306258** | **MZ673954** |
| *C. endophyticum* | YN32-6 | MH636511 | MH681390 | MH622453 | MH622589 | MH622721 |
| *C. endophyticum* | YN32-2 | MH636509 | MH681388 | MH622451 | MH622587 | MH622719 |
| *C. endophyticum* | YN1A4 | KU251561 | KU252015 | KU251909 | KU251642 | KU252169 |
| *C. endophyticum* | YN1A5 | KU251560 | KU252014 | KU251908 | KU251641 | KU252168 |
| *C. endophyticum* | CAUG28 | KP145441 | KP145413 | KP145385 | KP145329 | KP145469 |
| ***C. fici-septicae*** | **MFLU 19-2770 ^T^** | **MW114367** | **MW183774** | **MW177701** | **MW151585** |  |
| ***C. fructicola*** | **ICMP 18581 ^T^** | **JX010165** | **JX010033** | **JX009866** | **FJ907426** | **JX010405** |
| *C. fructicola* (≡*C. ignotum*) | CBS 125397 | JX010173 | JX010032 |  | JX009581 |  |
| *C. fructicola* | MFLUCC 17-1752 | OR828931 | OR840868 | OR840856 | OR840845 | OR840862 |
| *C. fructicola* | MFLUCC 17-1753 | OR828932 | OR840869 | OR840857 | OR840846 | OR840863 |
| *C. fructicola* | MFLUCC 22-0181 | OQ048649 | OQ067350 | OQ067349 | OQ067348 | OQ067351 |
| *C. fructicola* | MFLUCC 22-0182 | OQ048650 | OQ067354 | OQ067353 | OQ067352 | OQ067355 |
| *C. fructicola* (≡*C. mengyinense*) | SAUCC 200702 | MW786742 | MW846240 | MW883686 | MW883695 | MW888970 |
| ***C. fructivorum*** | **BPI 884103 ^T^** | **JX145145** | **MZ664047** | **MZ799259** | **MZ664126** | **JX145196** |
| ***C. gardeniae*** | **GUCC 12049 ^T^** | **OP722995** | **OP737963** | **OP715766** | **OP715801** | **OP720858** |
| ***C. gloeosporioides*** | **IMI 356878 ^T^** | **JQ005152** | **JQ005239** | **JQ005326** | **JQ005500** | **JQ005587** |
| *C. gloeosporioides* | ICMP 18730 | JX010157 | JX009981 | JX009861 | JX009548 |  |
| *C. gloeosporioides* | LGMF800 | KM278577 | KM257052 | KJ579936 | KJ569194 | KJ579891 |
| *C. gloeosporioides* | LGMF748 | KM257023 | KM257047 | KJ579931 | KJ569189 | KJ579839 |
| ***C. gracile*** | **YMF 1.06939 ^T^** | **OK030868** | **OK513671** | **OK513567** | **OK513607** | **OK513637** |
| *C. gracile* | YMF 1.07329 |  | OK513672 |  | OK513608 | OK513638 |
| ***C. grevilleae*** | **CBS 132879 ^T^** | **KC297078** | **KC297010** | **KC296987** | **KC296941** | **KC297102** |
| ***C. grossum*** | **CGMCC 3.17614 ^T^** | **KP890165** | **KP890159** | **KP890153** | **KP890141** | **KP890171** |
| ***C. guiyangense*** | **CNUCC 823-1-1-1 ^T^** | **PP812207** | **PP819551** | **PP819547** | **PP830762** | **PP830770** |
| *C. guiyangense* | CNUCC 823-1-1-2 | PP812208 | PP819552 | PP819548 | PP830763 | PP830771 |
| ***C. hebeiense*** | **MFLUCC 13-0726 ^T^** | **KF156863** | **KF377495** | **KF289008** | **KF377532** | **KF288975** |
| ***C. hederiicola*** | **MFLU 15-0689 ^T^** | **MN631384** |  | **MN635794** | **MN635795** |  |
| ***C. helleniense*** | **CBS 142418 ^T^** | **KY856446** | **KY856270** | **KY856186** | **KY856019** | **KY856528** |
| ***C. henanense*** | **LC3030 ^T^** | **KJ955109** | **KJ954810** | **MZ799256** | **KM023257** | **KJ955257** |
| ***C. horii*** | **NBRC 7478 ^T^** | **GQ329690** | **GQ329681** | **JX009752** | **JX009438** | **JX010450** |
| ***C. horii*** | **ICMP 17968** | **JX010212** | **GQ329682** | **JX009811** | **JX009547** | **JX010378** |
| ***C. hystricis*** | **CBS 142411 ^T^** | **KY856450** | **KY856274** | **KY856190** | **KY856023** | **KY856532** |
| ***C. jiangxiense*** | **CGMCC 3.17363 ^T^** | **KJ955201** | **KJ954902** | **MZ799257** | **KJ954471** | **KJ955348** |
| *C. jiangxiense* | SYD-9 | OR467495 | OR472539 | OR472537 | OR472535 | OR472541 |
| *C. jiangxiense* | SYD-4 | OR467494 | OR472538 | OR472536 | OR472534 | OR472540 |
| ***C. jixiense*** | **CNUCC 5-21-2-1 ^T^** | **PP812209** | **PP819553** | **PP819549** | **PP830764** | **PP830772** |
| *C. jixiense* | CNUCC 5-21-2-2 | PP812210 | PP819554 | PP819550 | PP830765 | PP830773 |
| ***C. juglandicola*** | **CGMCC3.24312 ^T^** | **OQ263015** | **OQ282973** | **OR004793** | **OQ282966** | **OQ282980** |
| *C. juglandicola* | CGMCC3.24313 | OQ263018 | OQ282977 | OR004797 | OQ282970 | OQ282984 |
| ***C. kahawae*** | **ICMP:17816 ^T^** | **JX010231** | **JX010012** | **JX009813** | **JX009452** | **JX010444** |
| *C. kahawae* | ICMP:17811 | JX010233 | JX009970 | JX009817 | JX009555 | JX010430 |
| ***C. kunmingense*** | **GUCC 12053 ^T^** | **OP722975** | **OP737965** | **OP715769** | **OP715804** | **OP720861** |
| ***C. ledongense*** | **CGMCC 3.18888 ^T^** | **MG242008** | **MG242016** | **MG242018** | **MG242014** | **MG242010** |
| *C. ligustri* | GUCC 12111 T | OP722988 | OP737968 | OP715773 | OP740216 | OP720864 |
| ***C. lumnitzerae*** | **MFLUCC 23-0291 ^T^** | **OR856122** | **OR886391** | **OR886403** | **OR886394** | **OR886397** |
| ***C. makassarense*** | **CBS 143664 ^T^** | **MH728812** | **MH728820** | **MH805850** | **MH781480** | **MH846563** |
| *C. makassarense* | CPC 28556 | MH728815 | MH728821 | MH805848 | MH781478 | MH846561 |
| ***C. musae*** | **CBS 116870 ^T^** | **JX010146** | **JX010050** | **JX009896** | **JX009433** | **JX010413** |
| ***C. nanjingense*** | **CFCC 58940 ^T^** | **OQ456155** | **OQ507144** | **OQ507141** | **OQ507149** | **OQ507147** |
| *C. nanjingense* | CFCC 58939 | OQ456154 | OQ507143 | OQ507140 | OQ507150 | OQ507146 |
| ***C. nullisetosum*** | **YMF 1.06946 ^T^** | **OK030872** | **OK513675** | **OK513571** | **OK513611** | **OK513641** |
| ***C. nupharicola*** | **CBS 470.96 ^T^** | **JX010187** | **JX009972** | **JX009835** | **JX009437** | **JX010398** |
| ***C. oblongisporum*** | **YMF 1.06938 ^T^** | **OK030874** | **OK513677** | **OK513573** |  | **OK513643** |
| *C. osmanthicola* | AG36 | OL772733 | OL854168 | OL854154 | OM100914 | OL854182 |
| *C. osmanthicola* | AG42 | OL772734 | OL854169 | OL854155 | OM100915 | OL854183 |
| ***C. peakense*** | **CGMCC 3.24308 ^T^** | **OQ263017** | **OQ282975** | **OR004795** | **OQ282968** | **OQ282982** |
| *C. peakense* | CGMCC 3.24307 | OQ263016 | OQ282974 | OR004794 | OQ282967 | OQ282981 |
| ***C. perseae*** | **CBS 141365 ^T^** | **KX620308** | **KX620242** | **MZ799260** | **KX620145** | **KX620341** |
| ***C. polypodialium*** | **MFLU 22-0234 ^T^** | **OP802361** | **OP801720** | **OP801702** | **OP801685** | **OP801739** |
| *C. polypodialium* | MFLU 22-0238 | OP802377 | OP801730 | OP801712 | OP801695 | OP801750 |
| ***C. populi*** | **HMBFU191 ^T^** | **AB632347** | **JN211081** |  | **JN184704** | **JN862898** |
| *C. populi* | HMBFU173 | AB632349 | JN211084 |  | JN184707 | JN862900 |
| ***C. proteae*** | **CBS 132882 ^T^** | **KC297079** | **KC297009** | **KC296986** | **KC296940** | **KC297101** |
| ***C. pseudotheobromicola*** | **MFLUCC 18-1602 ^T^** | **MH817395** | **MH853675** | **MH853678** | **MH853681** | **MH853684** |
| ***C. psidii*** | **CBS 145.29 ^T^** | **JX010219** | **JX009967** | **JX009901** | **JX009515** | **JX010443** |
| ***C. queenslandicum*** | **ICMP 1778 ^T^** | **JX010276** | **JX009934** | **JX009899** | **JX009447** | **JX010414** |
| ***C. rhexiae*** | **CBS 133134 ^T^** | **JX145128** | **MZ664046** | **MZ799258** | **MZ664127** | **JX145179** |
| ***C. salsolae*** | **ICMP 19051 ^T^** | **JX010242** | **JX009916** | **JX009863** | **JX009562** | **JX010403** |
| ***C. siamense*** | **ICMP 18578 ^T^** | **FJ972613** | **FJ972575** | **JX009865** | **FJ907423** | **FJ907438** |
| *C. siamense* | MFLUCC 18-1162 | MN788676 | MN995328 | MN995335 | MN995334 | MN995329 |
| *C. siamense* | ICMP 18572 | JX010160 | JX010061 | JX009783 | JX009487 |  |
| *C. siamense* | ICMP 18739 | JX010161 | JX009921 | JX009794 | JX009484 |  |
| *C. siamense* | ICMP 18571 | JX010159 | JX009922 | JX009782 | JX009482 |  |
| *C. siamense* | MFLUCC 22-0138 | OP802366 | OP801723 | OP801705 | OP801688 | OP801742 |
| *C. siamense* | MFLU25-0400 | PX612250 | PX692532 | PX692520 | PX692526 | PX939442 |
| *C. siamense* | MFLU25-0401 | PX612251 | PX692533 | PX692521 | PX692527 | PX939443 |
| *C. siamense* | MFLU25-0402 | PX612252 | PX692534 | PX692522 | PX692528 | PX939444 |
| *C. siamense* | MFLU25-0403 | PX612253 | PX692535 | PX692523 | PX692529 | PX939445 |
| *C. siamense* | MFLU25-0404 | PX612254 | PX692536 | PX692524 | PX692530 | PX939446 |
| *C. siamense* | MFLU25-0405 | PX612255 | PX692537 | PX692525 | PX692531 | PX939447 |
| ***C. subhenanense*** | **YMF1.06865 ^T^** | **OK030883** | **OK513684** | **OK513581** | **OK513618** | **OK513647** |
| ***C. syzygiicola*** | **DNCL021 ^T^** | **KF242094** | **KF242156** |  | **KF157801** | **KF254880** |
| ***C. tainanense*** | **CBS 143666 ^T^** | **MH728818** | **MH728823** | **MH805845** | **MH781475** | **MH846558** |
| ***C. temperatum*** | **CBS 133122 ^T^** | **JX145159** | **MZ664045** | **MZ799254** | **MZ664125** | **JX145211** |
| ***C. thasutense*** | **MFLU 22-0206 ^T^** | **OP821902** | **OP831282** | **OP831281** | **OP831280** | **OP831283** |
| ***C. theobromicola*** | **CBS 124945 ^T^** | **JX010294** | **JX010006** | **JX009869** | **JX009444** | **JX010447** |
| ***C. ti*** | **ICMP 4832 ^T^** | **JX010269** | **JX009952** | **JX009898** | **JX009520** | **JX010442** |
| ***C. tomentosae*** | **ZHKUCC 21-0103 ^T^** | **OL708422** | **OL855850** | **OL855860** | **OL855870** | **OL855887** |
| ***C. tropicale*** | **CBS 124949 ^T^** | **JX010264** | **JX010007** | **JX009870** | **JX009489** | **JX010407** |
| *C. tropicale* | CBS 124946 | KC566806 | KC566660 | KC566373 | KC566952 | KC566228 |
| *C. tropicale* | CBS 124943 | JX010277 | JX010014 | JX009868 | JX009570 |  |
| *C. tropicale* | CPC 16260 | KC566807 | KC566661 | KC566374 | KC566953 | KC566229 |
| ***C. viniferum*** | **GZAAS 5.08601 ^T^** | **JN412804** | **JN412798** |  | **JN412795** | **JN412813** |
| ***C. wuxiense*** | **CGMCC 3.17894 ^T^** | **KU251591** | **KU252045** | **KU251939** | **KU251672** | **KU252200** |
| ***C. xanthorrhoeae*** | **CBS 127831 ^T^** | **JX010261** | **JX009927** | **JX009823** | **JX009478** | **JX010448** |
| ***C. xishuangbannaense*** | **MFLUCC 19-0107 ^T^** | **MW346469** | **MW537586** | **MW660832** | **MW652294** |  |
| ***C. yuanjiangense*** | **YMF 1.04996 ^T^** | **OK030885** | **OK513686** | **OK513583** | **OK513620** | **OK513649** |
| *C. yuanjiangense* | YMF 1.04997 | OK030886 | OK513687 | OK513584 | OK513621 | OK513650 |
| ***C. xishanense*** | **CNUCC 453-2-3-1 ^T^** | **PP481730** | **PP498763** | **PP498761** | **PP498767** | **PP498765** |
| *C. xishanense* | CNUCC 453-2-3-2 | PP481731 | PP498764 | PP498762 | PP498768 | PP498766 |
| ***C. acidae*** | **MFLUCC17-2659 ^T^** | **MG996505** | **MH003691** | **MH003694** | **MH003697** | **MH003700** |
| ***C. truncatum*** | **CBS151.35 ^T^** | **GU227862** | **GU228254** | **GU228352** | **GU227960** | **GU228156** |

**Table S4.** *Diaporthe* section Foeniculina with their respective GenBank accession numbers used in the phylogenetic analyses in this study.

| **Taxa** | **Strain** | **ITS** | ***TEF1-α*** | ***TUB2*** | ***CAL*** | ***HIS*** |
| --- | --- | --- | --- | --- | --- | --- |
| ***Diaporthe acutispora*** | **CGMCC3.18285 ^T^** | **KX986764** | **KX999155** | **KX999195** | **KX999274** | **KX999235** |
| ***D. anacardii*** | **CBS 720.97** | **KC343024** | **KC343750** | **KC343992** | **KC343266** | **KC343508** |
| *D. anacardii* (*D. macadamiae*) | BRIP 66526 |  | MN696527 | MN696538 | – | – |
| *D. anacardii* (*D. nebulae*) | Phom240 | KY511315 | MH708543 | KY511346 | – | – |
| *D. anacardii* (*D. nebulae*) | PMM1681 | KY511337 | MH708552 | KY511369 | – | – |
| *D. anacardii* (*D. phillipsii*) | CAA817 | MK792305 | MK828076 | MN000351 | MK883831 | MK871445 |
| *D. anacardii* (*D. phillipsii*) | CAA818 | MK792307 | MK828078 | MN000352 | MK883833 | MK871447 |
| *D. anacardii* (*D. portugallica*) | CPC 34247 | MH063905 | MH063911 | MH063917 | MH063893 | MH063899 |
| *D. anacardii* (*D. portugallica*) | CPC 34248 | MH063906 | MH063912 | MH063918 | MH063894 | MH063900 |
| *D. anacardii* (*D. velutina*) | CGMCC3.18286 | KX986790 | KX999182 | KX999223 | – | KX999261 |
| *D. anacardii* (*D. velutina*) | LC4419 | KX986789 | KX999181 | KX999222 | KX999286 | KX999260 |
| ***D. arecae*** | **CBS 161.64 ^T^** | **KC343032** | **KC343758** | **KC344000** | **KC343274** | **KC343516** |
| *D. arecae* | CBS 535.75 | KC343033 | KC343759 | KC344001 | KC343275 | KC343517 |
| *D. arecae* | BPPCA257 | MK111098 | MK117256 | MK122791 | – | – |
| *D. arecae* | PBMR340 | MK111086 | MK117271 | MK122805 | – | – |
| *D. arecae* | PBMR345 | MK111088 | MK117275 | MK122810 | – | – |
| *D. arecae* | CGMCC3.24301 (GZCC 22-0015) | OP056723 | OP150562 | OP150639 | OP150716 | OP150792 |
| *D. arecae* | GZCC 22-0038 | OP056723 | OP150562 | OP150639 | OP150716 | OP150792 |
| *D. arecae* | CGMCC3.24296 (GZCC 19-0124) | OP056688 | OP150527 | OP150605 | OP150684 | OP150759 |
| *D. arecae* | GZCC 19-0217 | OP056689 | OP150528 | OP150606 | OP150685 | OP150760 |
| *D. arecae* | GZCC 22-0023 | OP056690 | OP150529 | OP150607 | OP150686 | OP150761 |
| *D. arecae* | GZCC 22-0041 | OP056691 | OP150530 | OP150608 | OP150687 | OP150762 |
| *D. arecae* | GZCC 22-0047 | OP056692 | OP150531 | OP150609 | OP150688 | OP150763 |
| *D. arecae* | GZCC 22-0054 | OP056693 | OP150532 | OP150610 | OP150689 | OP150764 |
| *D. arecae* | GZCC 22-0052 | OP056710 | OP150549 | OP150627 | OP150703 | OP150780 |
| *D. arecae* | GZCC 22-0035 | OP056708 | OP150547 | OP150625 | OP150701 | OP150778 |
| *D. arecae* | GZCC 19-0079 | OP056705 | OP150544 | OP150622 | OP150698 | OP150775 |
| *D. arecae* | GZCC 19-0213 | OP056650 | OP150490 | OP150568 | OP150647 | OP150722 |
| *D. arecae* | GZCC 22-0011 | OP056651 | OP150491 | OP150569 | OP150648 | OP150723 |
| *D. arecae* | GZCC 22-0038 | OP056649 | OP150489 | OP150567 | OP150646 | OP150721 |
| *D. arecae* | GZCC 22-0017 | OP056721 | OP150560 | OP150637 | OP150714 | OP150790 |
| *D. arecae* | CGMCC3.24300 (GZCC 22-0036) | OP056709 | OP150548 | OP150626 | OP150702 | OP150779 |
| *D. arecae* | GZCC 22-0028 | OP056707 | OP150546 | OP150624 | OP150700 | OP150777 |
| *D. arecae* | GZCC 22-0062 | OP056711 | OP150550 | – | OP150704 | OP150781 |
| *D. arecae* | GZCC 22-0010 | OP056713 | OP150552 | OP150629 | OP150706 | OP150783 |
| *D. arecae* | GZCC 22-0058 | OP056716 | OP150553 | OP150630 | OP150707 | OP150784 |
| *D. arecae* | GZCC 22-0024 | OP056714 | OP150554 | OP150631 | OP150708 | OP150785 |
| *D. arecae* | GZCC 22-0042 | OP056715 | OP150555 | OP150632 | OP150709 | – |
| *D. arecae* | GZCC 22-0059 | OP056722 | OP150561 | OP150638 | OP150715 | OP150791 |
| *D. arecae* | CGMCC3.24302 (GZCC 22-0033) | OP056719 | OP150558 | OP150635 | OP150712 | OP150788 |
| *D. arecae* | GZCC 22-0050 | OP056720 | OP150559 | OP150636 | OP150713 | OP150789 |
| *D. arecae* (*D. acuta*) | CGMCC3.19600 | MK626957 | MK654802 | MK691225 | MK691125 | MK726161 |
| *D. arecae* (*D. acuta*) | PSCG046 | MK626958 | MK654803 | MK691224 | MK691124 | MK726162 |
| *D. arecae* (*D. anhuiensis*) | CNUCC 201902 | MN219727 | MN224669 | MN227009 | MN224550 | MN224557 |
| *D. arecae* (*D. anhuiensis*) | CNUCC 201901 | MN219718 | MN224668 | MN227008 | MN224549 | MN224556 |
| *D. arecae* (*D. arengae*) | CBS 114979 | KC343034 | KC343760 | KC344002 | KC343276 | KC343518 |
| *D. arecae* (*D. averrhoae*) | SCHM 3605 | AY618930 | – | – | – | – |
| *D. arecae* (*D. camelliaeoleiferae*) | HNZZ027 | MZ509555 | MZ504702 | MZ504718 | MZ504685 | MZ504696 |
| *D. arecae* (*D. camelliaeoleiferae*) | HNZZ030 | MZ509556 | MZ504708 | MZ504719 | MZ504686 | MZ504697 |
| *D. arecae* (*D. caricae-papayae*) | NIBM-ABIJP | MN335224 | – | – | – | – |
| *D. arecae* (*D. ceratozamiae*) | CBS 131306 | JQ044420 | – | – | – | – |
| *D. arecae* (*D. ceratozamiae*) | HCH260 | KU360597 | – | – | – | – |
| *D. arecae* (*D. cercidis*) | CFCC 52565 | MH121500 | MH121542 | MH121582 | MH121424 | MH121460 |
| *D. arecae* (*D. cercidis*) | CFCC 52566 | MH121501 | MH121543 | MH121583 | MH121425 | MH121461 |
| *D. arecae* (*D. chrysalidocarpi*) | SAUCC194.35 | MT822563 | MT855876 | MT855760 | MT855646 | MT855532 |
| *D. arecae* (*D. delonicis*) | MFLU 16-1059 | MT215490 | – | MT212209 | – | – |
| *D. arecae* (*D. drenthii* | BRIP 66524 | MN708229 | MN696526 | MN696537 | – | – |
| *D. arecae* (*D. endocitricola* | ZHKUCC20-0012 | MT355682 | MT409336 | MT409290 | MT409312 | – |
| *D. arecae* (*D. endocitricola* | ZHKUCC20-0013 | MT355683 | MT409337 | MT409291 | MT409313 | – |
| *D. arecae* (*D. eugeniae*) | DPFT23 | MK110366 | MK117267 | MK122799 |  | – |
| *D. arecae* (*D. eugeniae*) | CBS 444.82 | KC343098 | KC343824 | KC344066 | KC343340 | KC343582 |
| *D. arecae* (*D. fraxini-angustifoliae*) | BRIP 54781 | JX862528 | JX862534 | KF170920 | – | – |
| *D. arecae* (*D. fulvicolor*) | PSCG051 | MK626859 | MK654806 | MK691236 | MK691132 | MK726163 |
| *D. arecae* (*D. fulvicolor*) | PSCG057 | MK626858 | MK654810 | MK691233 | MK691131 | MK726164 |
| *D. arecae* (*D. fulvicolor*) | GZCC 19-1025 | OP056706 | OP150545 | OP150623 | OP150699 | OP150776 |
| *D. arecae* (*D. guangxiensis*) | JZB320094 | MK335772 | MK523566 | MK500168 | MK736727 | – |
| *D. arecae* (*D. guangxiensis*) | JZB320091 | MK335769 | MK523564 | MK500165 | MK736724 | – |
| *D. arecae* (*D. huangshanensis*) | CNUCC201903 | MN219729 | MN224670 | MN227010 | – | MN224558 |
| *D. arecae* (*D. huangshanensis*) | CNUCC201904 | MN219730 | MN224671 | MN227011 | – | MN224559 |
| *D. arecae* (*D. hunanensis*) | HNZZ023 | MZ509550 | MZ504702 | MZ504714 | MZ504680 | MZ504691 |
| *D. arecae* (*D. hunanensis*) | HNZZ025 | MZ509551 | MZ504703 | MZ504715 | MZ504681 | MZ504692 |
| *D. arecae* (*D. krabiensis*) | MFLUCC 17-2481 | MN047101 | MN433215 | MN431495 | – | – |
| *D. arecae* (*D. limonicola*) | CBS 142549 | MF418422 | MF418501 | MF418582 | MF418256 | MF418342 |
| *D. arecae* (*D. limonicola*) | CPC 27869 | MF418419 | MF418498 | MF418579 | MF418253 | MF418339 |
| *D. arecae* (*D. liquidambaris*) | SCHM 3621 | AY601919 | – | – | – | – |
| *D. arecae* (*D. liquidambaris*) | XSD-15 | EU273505 | – | – | – | – |
| *D. arecae* (*D. litchicola*) | BRIP 54900 | JX862533 | JX862539 | KF170925 | – | – |
| *D. arecae* (*D. loropetali*) | SCHM 3615 | AY601917 | – | – | – | – |
| *D. arecae* (*D. meliae*) | CFCC 53089 | MK432657 | ON081654 | MK578057 | – | ON081662 |
| *D. arecae* (*D. meliae*) | CFCC 53090 | MK432658 | ON081655 | MK578058 | – | ON081663 |
| *D. arecae* (*D. melitensis*) | CBS 142551 | MF418424 | MF418503 | MF418584 | MF418258 | MF418344 |
| *D. arecae* (*D. melitensis*) | CPC 27875 | MF418425 | MF418504 | MF418585 | MF418259 | MF418345 |
| *D. arecae* (*D. millettiae*) | GUCC9167 | MK398674 | MK480609 | MK502089 | MK502086 |  |
| *D. arecae* (*D. musigena*) | CBS 129519 | KC343143 | KC343869 | KC344111 | KC343385 | KC343627 |
| *D. arecae* (*D. musigena*) | HKFZL006 | MK050110 | MK054238 | MK079660 | – | – |
| *D. arecae* (*D. nelumbonis*) | R Kirschner 4114 | KT821501 | – | LC086652 | – | – |
| *D. arecae* (*D. nelumbonis*) | A-SER3 | MK907914 | – | – | – | – |
| *D. arecae* (*D. oculi*) | HHUF 30565 | LC373514 | LC373516 | LC373518 | – | – |
| *D. arecae* (*D. osmanthi*) | GUCC9165 | MK303388 | MK480610 | MK502091 | – | – |
| *D. arecae* (*D. pandanicola*) | MFLUCC 17-0607 | MG646974 |  | MG646930 | – | – |
| *D. arecae* (*D. pascoei*) | BPPCA147 | MK111091 | MK117255 | MK122790 | – | – |
| *D. arecae (D. perseae)* | CBS 151.73 | KC343173 | – | – | KC343415 | – |
| *D. arecae* (*D. pescicola*) | MFLUCC 16-0105 | KU557555 | KY400831 | KU557579 | KU557603 |  |
| *D. arecae* (*D. pescicola*) | PSCG036 | MK626855 | MK654796 | MK691226 | MK691116 | MK726159 |
| *D. arecae* (*D. phyllanthicola*) | SCHM 3680 | AY620819 | – | – | – | – |
| *D. arecae* (*D. phyllanthicola*) | RS 129 | MK398278 | – | – | – | – |
| *D. arecae* (*D. podocarpi-macrophylli*) | CGMCC3.18281 | KX986774 | KX999167 | KX999207 | KX999278 | KX999246 |
| *D. arecae* (*D. podocarpi-macrophylli*) | LC6229 | KX986771 | KX999164 | KX999204 | KX999277 | KX999243 |
| *D. arecae* (*D. pseudomangiferae*) | CBS 101339 | KC343181 | KC343907 | KC344149 | KC343423 | KC343665 |
| *D. arecae* (*D. pseudomangiferae*) | CBS 388.89 | KC343182 | KC343908 | KC344150 | KC343424 | KC343666 |
| *D. arecae* (*D. pseudooculi*) | HHUF 30617 | NR_161019 | LC373517 | LC373519 |  |  |
| *D. arecae* (*D. pseudooculi*) | B3180 | MT043790 |  |  |  |  |
| *D. arecae* (*D. pseudophoenicicola*) | CBS 462.69 | KC343184 | KC343910 | KC344152 | KC343426 | KC343668 |
| *D. arecae* (*D. pseudophoenicicola*) | CBS 176.77 | KC343183 | KC343909 | KC344151 | KC343425 | KC343667 |
| *D. arecae* (*D. pterocarpicola*) | MFLUCC 10-0580a | JQ619887 | JX275403 | JX275441 | JX197433 | – |
| *D. arecae* (*D. pterocarpicola*) | MFLUCC 10-0580b | JQ619887 | JX275403 | JX275441 | JX197433 | – |
| *D. arecae* (*D. schimae*) | CFCC 53103 | MK432640 | MK578116 | MK578043 | MK442962 | MK442987 |
| *D. arecae* (*D. schimae*) | CFCC 53104 | MK432641 | MK578117 | MK578044 | MK442963 | MK442988 |
| *D. arecae* (*D. searlei*) | BRIP 66528 | MN708231 | – | MN696540 | – | – |
| *D. arecae* (*D. sennae*) | CFCC 51636 | KY203724 | KY228885 | KY228891 | KY228875 |  |
| *D. arecae* (*D. sennae*) | CFCC 51637 | KY203725 | KY228886 | KY228892 | KY228876 |  |
| *D. arecae* (*D. spinosa*) | PSCG 383 | MK626849 | MK654811 | MK691234 | MK691129 | MK726156 |
| *D. arecae* (*D. spinosa*) | PSCG 279 | MK626925 | MK654801 | MK691235 | MK691126 | MK726155 |
| *D. arecae* (*D. taiwanensis*) | NTUCC 18-105-1 | MT241257 | MT251199 | MT251202 | MT251196 | – |
| *D. arecae* (*D. taiwanensis*) | NTUCC 18-105-2 | MT241258 | MT251200 | MT251203 | MT251197 | – |
| *D. arecae* (*D. taoicola*) | MFLUCC 16-0117 | KU557567 | KU557636 | KU557591 | – | – |
| *D. arecae* (*D. viniferae*) | JZB320071 | MK341550 | MK500107 | MK500112 | MK500119 | – |
| *D. arecae* (*D. viniferae*) | JZB320072 | MK341551 | MK500108 | MK500113 | MK500120 | – |
| *D. arecae* | KUC21217 | KT207733 | – | KT207633 | – | – |
| *D. arecae* | KUC21243 | KT207761 | – | KT207659 | – | – |
| *D. arecae* | MFLU25-0406 | PX612258 | PX939438 | PX933452 | PX933456 | PX933460 |
| *D. arecae* | MFLU25-0407 | PX612256 | PX939440 | PX933454 | PX933458 | PX933462 |
| *D. arecae* | MFLU25-0408 | PX612259 | PX939439 | PX933453 | PX933457 | PX933461 |
| *D. arecae* | MFLU25-0409 | PX612257 | PX939441 | PX933455 | PX933459 | PX933463 |
| ***D. aseana*** | **MFLUCC 12-0299a ^T^** | **KT459414** | **KT459448** | **KT459432** | **KT459464** | – |
| *D. aseana* (*D. tectonigena*) | MFLUCC 12-0767 | KX986782 | KX999174 | KX999214 | KX999284 | KX999254 |
| *D. aseana* (*D. tectonigena*) | LC6512 | KU712429 | KU749371 | KU743976 | KU749358 | – |
| ***D. biconispora*** | **ZJUD62 ^T^** | **KJ490597** | **KJ490476** | **KJ490418** | **MT898460** | **KJ490539** |
| *D. biconispora* | ZJUD61 | KJ490596 | KJ490475 | KJ490417 | – | KJ490538 |
| *D. biconispora* (*D. longiconidialis*) | ZHKUCC 22-0058 | ON322887 | ON315044 | ON315076 | – | ON315017 |
| *D. biconispora* (*D. longiconidialis*) | ZHKUCC 22-0059 | ON322888 | ON315045 | ON315077 | – | ON315018 |
| *D. biconispora* (*D. pometiae*) | SAUCC 194.72 | MT822600 | MT855912 | MT855797 | MT855679 | MT855568 |
| *D. biconispora* (*D. salsuginosa*) | NFCCI 4385 | MN061372 | – | MN431500 | – | – |
| *D. biconispora* (*D. tersa*) | BCKSKMP-8 | MG049670 | – | – | – | – |
| *D. biconispora* (*D. tersa*) | FS441 | MK592793 | – | – | – | – |
| ***D. camelliae-sinensis*** | **SAUCC194.92 ^T^** | **MT822620** | **MT855932** | **MT855817** | **MT855699** | **MT855588** |
| *D. camelliae-sinensis* | SAUCC 194.103 | MT822631 | MT855943 | MT855828 | MT855710 | MT855599 |
| ***D. canthii*** | **CBS 132533 ^T^** | **JX069864** | **KC843120** | **KC843230** | **KC843174** |  |
| ***D. chamaeropis*** | **CBS 454.81 ^T^** | **KC343048** | **KC343774** | **KC344016** | **KC343290** | **KC343532** |
| *D. chamaeropis* | CBS 753.70 | KC343049 | KC343775 | KC344017 | KC343291 | KC343533 |
| *D. chamaeropis* (*D. cytosporella*) | AR5149 | KC843309 | KC843118 | KC843223 | KC843143 | – |
| *D. chamaeropis* (*D. cytosporella*) | FAU 461 | KC843307 | KC843116 | KC843221 | KC843141 | – |
| *D. cinerascens* | CBS 719.96 | KC343050 | KC343776 | KC344018 | KC343292 | KC343534 |
| ***D. cissampeli*** | **CPC 27302 ^T^** | **KX228273** |  | **KX228384** | – | **KX228366** |
| ***D. corylicola*** | **CFCC 53986 ^T^** | **MW839880** | **MW815894** | **MW883977** | **MW836684** | **MW836717** |
| *D. decedens* | CBS 109772 | KC343059 | KC343785 | KC344027 | KC343301 | KC343543 |
| *D. decedens* | CBS 114281 | KC343060 | KC343786 | KC344028 | KC343302 | KC343544 |
| ***D. diospyricola*** | **CBS 136552 ^T^** | **KF777156** | – | – | – | – |
| ***D. donglingensis*** | **CFCC 56581 ^T^** | **OM956090** | **ON157986** | **ON158021** | – | **ON157951** |
| *D. donglingensis* | CFCC 57432 | OM956091 | ON157987 | ON158022 | – | ON157952 |
| ***D. elaeagni-glabrae*** | **CGMCC3.18287 ^T^** | **KX986779** | **KX999171** | **KX999212** | **KX999281** | **KX999251** |
| *D. elaeagni-glabrae* | LC4806 | KX986780 | KX999172 | KX999213 | KX999282 | KX999252 |
| ***D. foeniculina*** | **CBS 111553 ^T^** | **KC343101** | **KC343827** | **KC344069** | **KC343343** | **KC343585** |
| *D. foeniculina* | AR5151 | KC843303 | KC843112 | KC843217 | KC843137 | – |
| *D. foeniculina* | CBS 123208 | KC343104 | KC343830 | KC344072 | KC343346 | KC343588 |
| *D. foeniculina* | FAU 460 | KC843304 | KC843113 | KC843218 | KC843138 |  |
| *D. foeniculina* | ICMP 12285 | KC145853 | KC145937 | – | – | – |
| *D. foeniculina* (*D. baccae*) | CBS 136971 | KJ160564 | KJ160596 | – | – | – |
| *D. foeniculina* (*D. baccae*) | CBS 136972 | KJ160565 | KJ160597 | MF418509 | MG281695 | MF418264 |
| *D. foeniculina* (*D. nigra*) | JZBH320170 | MN653009 | MN892277 | MN887113 | – | – |
| *D. foeniculina* (*D. ravennica*) | MFLUCC 15-0480 | – | KX426703 | KX377688 | – | – |
| *D. foeniculina* (*D. ravennica*) | MFLUCC 17-1029 | KY964191 | KY964147 | KY964075 | – | – |
| *D. foeniculina* (*D. rhusicola*) | CPC 18191 | JF951146 | – | – | – | – |
| *D. foeniculina* (*D. rumicicola*) | JZB320006 | MK066126 | MK078545 | MK078546 | – | – |
| *D. foeniculina* (*D. rumicicola*) | MFLUCC 18-0739 | MH846233 | – | MK049555 | – | – |
| *D. foeniculina* (*D. zaobaisu*) | PSCG 031 | MK626922 | MK654855 | MK691245 | – | MK726207 |
| *D. foeniculina* (*D. zaobaisu*) | PSCG 032 | MK626923 | MK654856 | MK691246 | – | MK726208 |
| ***D. forlicesenica*** | **MFLUCC 17-1015 ^T^** | **KY964215** | **KY964171** | **KY964099** | – | – |
| ***D. hickoriae*** | **CBS 145.26 ^T^** | **KC343118** | **KC343844** | **KC344086** | **KC343360** | **KC343602** |
| ***D. hongkongensis*** | **CBS 115448 ^T^** | **KC343119** | **KC343845** | **KC344087** | **KC343361** | **KC343603** |
| *D. hongkongensis* | ZJUD74 | KJ490609 | KJ490488 | KJ490430 | – | KJ490551 |
| *D. hongkongensis* (*D. araliaechinensis*) | GUCC 412.7 | OP581218 | OP688523 | OP688548 | – | – |
| *D. hongkongensis* (*D. araliaechinensis*) | GUCC 412.71 | OP581219 | OP688524 | OP688549 | – | – |
| *D. hongkongensis* (*D. araliaechinensis*) | GUCC 412.72 | OP581220 | OP688525 | OP688550 | – | – |
| *D. hongkongensis* (*D. australiana*) | BRIP 66145 | MN708222 | MN696522 | MN696530 | – | – |
| *D. hongkongensis* (*D. eucalyptorum*) | CBS 132525 | JX069862 | – | – | – | – |
| *D. hongkongensis* (*D. lagerstroemiae*) | SCHM 3608 | AY622994 | – | – | – | – |
| *D. hongkongensis* (*D. lithocarpus*) | CGMCC3.15175 | KC153104 | KC153095 | KF576311 | KF576236 | – |
| *D. hongkongensis* (*D. lithocarpus*) | CGMCC3.15178 | KC153103 | KC153094 | – | – | – |
| *D. hongkongensis* (*D. rhodomyrti*) | CFCC 53101 | MK432643 | MK578119 | MK578046 | MK442965 | MK442990 |
| *D. hongkongensis* (*D. rhodomyrti*) | CFCC 53102 | MK432644 | MK578120 | MK578047 | MK442966 | MK442991 |
| *D. hongkongensis* (*D. salinicola*) | MFLUCC 18-0553 | MN047098 | MN077073 | – | – | – |
| *D. hongkongensis* (*D. salinicola*) | MFLU 17-2592 | MN047099 | MN077074 | – | – | – |
| ***D. hsinchuensis*** | **NTUPPMCC18-153-1 ^T^** | **MZ268409** | **MZ268472** | **MZ268430** | **MZ268451** | **MZ268493** |
| *D. hsinchuensis* | NTUPPMCC 18-153-2 | MZ268410 | MZ268473 | MZ268431 | MZ268452 | MZ268494 |
| ***D. isoberliniae*** | **CPC 22549 ^T^** | **KJ869133** | – | **KJ869245** | – | – |
| ***D. lenispora*** | **CGMCC3.20101 ^T^** | **MT385952** | **MT424687** | **MT424707** | **MW022472** | **MW022493** |
| *D. lenispora* | GZCC 19-0343 | MT797182 | MT793025 | MT793036 | MW022473 | MW022494 |
| *D. lenispora* | GZCC 22-0067 | OP056686 | OP150525 | OP150603 | OP150682 | OP150757 |
| *D. lenispora* | GZCC 22-0068 | OP056687 | OP150526 | OP150604 | OP150683 | OP150758 |
| ***D. maytenicola*** | **CPC 21896 ^T^** | **KF777157** | – | **KF777250** | – | – |
| *D. melastomatis* | SAUCC 194.80 | MT822608 | MT855920 | MT855805 | MT855687 | MT855576 |
| ***D. melatosmatis*** | **SAUCC194.55 ^T^** | **MT822583** | **MT855896** | **MT855780** | **MT855664** | **MT855551** |
| ***D. multiguttulata*** | **CFCC 53095 ^T^** | **MK432645** | **MK578121** | **MK578048** | **MK442967** | **KJ490575** |
| *D. multiguttulata* | CFCC 53099 | MK573958 | MK574633 | MK574653 | MK574593 | MK574613 |
| *D. oncostoma* | CBS 100454 | KC343160 | KC343886 | KC344128 | KC343402 | KC343644 |
| *D. oncostoma* | CBS 589.78 | KC343162 | KC343888 | KC344130 | KC343404 | KC343646 |
| *D. oncostoma* | GZCC 22-0022 | OP056724 | OP150563 | OP150640 | OP150717 | OP150793 |
| ***D. parapterocarpi*** | **CPC 22729 ^T^** | **KJ869138** | – | **KJ869248** | – | – |
| ***D. parvae*** | **PSCG034 ^T^** | **MK626919** | **MK654858** | **MK691248** | – | **MK726210** |
| *D. parvae* | PSCG035 | MK626920 | MK654859 | MK691249 | MK691169 | MK726211 |
| ***D. poincianellae*** | **URM 7932 ^T^** | **MH989509** | **MH989538** | **MH989537** | **MH989540** | **MH989539** |
| ***D. psoraleae*** | **CBS 136412 ^T^** | **KF777158** | **KF777245** | **KF777251** | – | – |
| ***D. pterocarpi*** | **MFLUCC 10-0571 ^T^** | **JQ619899** | **JX275416** | **JX275460** | **JX197451** | – |
| *D. pterocarpi* | MFLUCC 10-0575 | JQ619901 | JX275418 | JX275462 | JX197453 | – |
| *D. pterocarpi* (*D. inconspicua*) | CBS 133813 | KC343123 | KC343849 | KC344091 | KC343365 | KC343607 |
| *D. pterocarpi* (*D. inconspicua*) | LGMF922 | KC343124 | KC343850 | KC344092 | KC343366 | KC343608 |
| *D. pterocarpi* (*D. lutescens*) | SAUCC194.36 | MT822564 | MT855877 | MT855761 | MT855647 | MT855533 |
| *D. pterocarpi* (*D. pseudoinconspicua*) | URM 7873 | MH122535 | MH122530 | MH122521 | MH122525 | MH122518 |
| *D. pterocarpi* (*D. pseudoinconspicua*) | URM 7874 | MH122538 | MH122533 | MH122524 | MH122528 | MH122517 |
| ***D. pungensis*** | **SAUCC194.112 ^T^** | **MT822640** | **MT855952** | **MT855837** | **MT855719** | **MT855607** |
| *D. pungensis* | SAUCC 194.89 | MT822617 | MT855929 | MT855814 | MT855696 | MT855585 |
| ***D. saccharata*** | **CBS 116311 ^T^** | **KC343190** | **KC343916** | **KC344158** | **KC343432** | **KC343674** |
| *D. sophorae* | JAC12639 | MK432728 | – | – | – | – |
| *D. sophorae* | PDD95984 | JQ694115 | – | – | – | – |
| ***D. stictica*** | **CBS 370.54 ^T^** | **KC343212** | **KC343938** | **KC344180** | **KC343454** | **KC343696** |
| *D. tanakae* | MAFF 410127 | AB245075 | – | – | – | – |
| *D. tanakae* | MAFF 410600 | AB245077 | – | – | – | – |
| ***D. undulata*** | **CGMCC3.18293 ^T^** | **KX986798** | **KX999190** | **KX999230** | – | **KX999269** |
| *D. undulata* | LC8111 | KY491546 | KY491556 | KY491566 | – | – |
| ***D. vangueriae*** | **CPC 22703 ^T^** | **KJ869137** |  | **KJ869247** | – | – |
| *D. vangueriae* (*D. macintoshii*) | BRIP 55064a | KJ197289 | KJ197251 | KJ197269 | – | – |
| *D. vangueriae* (*D. macintoshii*) | CAF013 | KY420947 | – | – | – | – |
| *D. vangueriae* (*D. macintoshii*) | CAF027 | KU821458 | – | – | – | – |
| ***D. vawdreyi*** | **BRIP 57887a ^T^** | **KR936126** | **KR936129** | **KR936128** | – | – |
| ***D. xishuangbanica*** | **CGMCC3.18283 ^T^** | **KX986784** | **KX999176** | **KX999217** | – | – |
| *D. xishuangbanica* | CGMCC3.18282 | KX986783 | KX999175 | KX999216 | – | KX999255 |
| ***D. xunwuensis*** | **CFCC 53085 ^T^** | **MK432663** | **MK578137** | **MK578063** | **MK442983** | **MK443008** |
| *D. xunwuensis* | CFCC 53086 | MK432664 | MK578138 | MK578064 | MK442984 | MK443009 |
| ***D. zhaoqingensis*** | **ZHKUCC 22-0056 ^T^** | **ON322885** | – | **ON315074** | **ON315000** | **ON315015** |
| *D. zhaoqingensis* | ZHKUCC 22-0057 | ON322886 | ON315043 | ON315075 | – | ON315016 |
| ***D. eres*** | **AR5193 ^T^** | **KJ210529** | **KJ210550** | **KJ420799** | **KJ434999** | **KJ420850** |

**Table S5.** Estimated nucleotide-substitution model parameters and tree statistics generated in the phylogenetic analyses in this study.

| **Taxa** | **Estimated base frequencies** | **Substitution rates** | **Gamma distribution** | **Tree length** |
| --- | --- | --- | --- | --- |
| *Brunswickiella* | A = 0.245176,  C = 0.247395,  G = 0.292104,  T = 0.215325 | AC = 1.464833,  AG = 3.053193,  AT = 1.352029,  CG = 1.034239,  CT = 6.500001,  GT = 1.000000 | 0.160014 | 1.933296 |
| *Pestalotiopsis* | A = 0. 237403,  C = 0. 294890,  G = 0. 215575,  T = 0. 252132 | AC = 1.146458,  AG = 3.700510,  AT = 1.328822,  CG = 1.062185,  CT = 5.402064,  GT = 1.000000 | 0. 297230 | 2.603154 |
| *Colletotrichum* | A = 0.228941,  C = 0.297650,  G = 0.243859,  T = 0.229549 | AC = 1.020727,  AG = 2.992079,  AT = 1.319689,  CG = 0.822879,  CT = 4.809791,  GT = 1.000000 | 0.423076 | 1.269077 |
| *Diaporthe* | A = 0.213736,  C = 0.318953,  G = 0.239512,  T = 0.227799 | AC = 1.114899,  AG = 3.169680,  AT = 1.105539,  CG = 0.697192,  CT = 4.186189,  GT = 1.000000 | 0.407006 | 5.261426 |
