# Supplementary material for: Putative palm pathogens: Novel species and new host records of leaf disease-associated microfungi (Ascomycota) on Nypa fruticans in Thailand
Source: MycoKeys. 2026 Apr 6;130:315–53. doi: 10.3897/mycokeys.130.175665 (PMC13077315; doi:10.3897/mycokeys.130.175665)
Supplement: Supplementary material 2 — Worldwide checklist of microfungi reported on Nypa fruticans, their distribution and lifestyle/s [file mycokeys-130-315-s002.docx]

**Table S6**. Worldwide checklist of microfungi reported on *Nypa fruticans*, their distribution and lifestyle/s.

| **Taxa** | **Lifestyle** | **Plant part/s** | **Distribution** | **References** |
| --- | --- | --- | --- | --- |
| *Acrogenospora sphaerocephala* | saprobe | petiole | Thailand | Loilong et al. 2012; Devadatha et al. 2021 |
| *Acuminatispora palmarum* | saprobe | petiole | Thailand | Zhang et al. 2018 |
| *Akanthomyces muscarius* | endophyte | leaves | Thailand | Vinit et al. 2018 |
| *Aniptodera intermedia* | saprobe | base petiole | Malaysia | Hyde 1994a; Hyde et al. 1999 |
| *Aniptodera chesapeakensis* | saprobe | petiole, rachis, fronds | Brunei Darussalam, Indonesia, Thailand | Hyde & Alias 2000; Loilong et al. 2012; Devadatha et al. 2021 |
| *Aniptodera mangrovei* | saprobe | petiole | Brunei Darussalam, Malaysia, Philippines, Thailand | Devadatha et al. 2021 |
| *Aniptodera nypae* | saprobe | petiole, fronds | Malaysia, Thailand | Loilong et al. 2012; Devadatha et al. 2021 |
| *Anthostomella eructans* |  | petiole | Brunei Darussalam | Hyde & Sarma 2006; Devadatha et al. 2021 |
| *Anthostomella foliatella* | saprobe | decayed leaves | Thailand | Zhang et al. 2024 |
| *Anthostomella mangrovei* | saprobe | rachis | Thailand | Zhang et al. 2024 |
| *Anthostomella nypae* | saprobe | base petiole | Brunei Darussalam, Malaysia, Thailand | Hyde et al. 1999a; Devadatha et al. 2021 |
| *Anthostomella nypensis* | saprobe | petiole | Brunei Darussalam, Malaysia, Philippines | Hyde et al. 1999a; Besitulo et al. 2010; Devadatha et al. 2021 |
| *Anthostomella nypicola* | saprobe | base petiole | Malaysia | Hyde et al. 1999a |
| *Apioclypea nypicola* | saprobe | petiole, rachis, midrib | Malaysia | Hyde et al. 1998; Hyde & Alias 2000 |
| *Apophysomyces* sp. | endophyte | stem | Indonesia | Nasution et al. 2024 |
| *Arecophila nypae* | saprobe | petiole | Malaysia | Hyde et al. 1996 |
| *Aspergillus* sp. | endophyte | stem, frond, fruit mesocarp | Indonesia | Nasution et al. 2024 |
| *Astrocystis rachidis* | saprobe | petiole | Malaysia | Fröhlich & Hyde 2000 |
| *Astrocystis selangorensis* | saprobe | rachis | Malaysia | Smith & Hyde 2001 |
| *Astrosphaeriella aquatica* | saprobe | petiole | Brunei Darussalam | Hyde & Sarma 2006; Devadatha et al. 2021 |
| *Astrosphaeriella asiana* | saprobe | petiole | Thailand | Hyde 1995; Hyde & Alias 2000; Devadatha et al. 2021 |
| *Astrosphaeriella nipicola* | saprobe | petiole | Brunei Darussalam, Indonesia, Malaysia | Hyde et al. 1997; Hyde & Alias 2000; Poonyth et al. 2000 |
| *Astrosphaeriella nypae* | saprobe | fronds, petiole | Brunei Darussalam, Malaysia, Thailand | Hyde et al. 1992a; Devadatha et al. 2021 |
| *Astrosphaeriella striatispora* | saprobe | wood | Australia, Brunei Darussalam, Indonesia, Malaysia | Hyde et al. 1997; Loilong et al. 2012 |
| *Bacusphaeria nypae* | saprobe | base petiole | Malaysia | Abdel-Wahab et al. 2017 |
| *Brunswickiella nypae* | possible pathogen | leaves | Thailand | This study |
| *Cancellidium applanatum* | saprobe | petiole, fronds | Thailand | Loilong et al. 2012 |
| *Carinispora nypae* | saprobe | petiole, fronds | Brunei Darussalam, Malaysia | Hyde et al. 1993;Hyde & Alias 2000; Zhang et al. 2012 |
| *Carinispora velatispora* | saprobe | petiole, fronds | Thailand | Loilong et al. 2012 |
| *Chaetospermum* sp. | saprobe | petiole, fronds | Thailand | Loilong et al. 2012 |
| *Cladosporium* sp. | endophyte | fruit mesocarp | Indonesia | Nasution et al. 2024 |
| *Colletotrichum* sp. | endophyte | leaves, bark | Brunei Darussalam | Mohamad et al. 2023 |
| *Colletotrichum siamense* | possible pathogen | leaves | Thailand | This study |
| *Cosmospora* sp. | saprobe | petiole, fronds | Thailand | Loilong et al. 2012 |
| *Cucullosporella mangrovei* | saprobe | petiole, rachis | Brunei Darussalam | Hyde & Alias 2000; Devadatha et al. 2021 |
| *Cumulospora* sp. | saprobe | petiole, fronds | Thailand | Loilong et al. 2012 |
| *Cyanoporus fuligo (Grammothele fuligo)* | saprobe | petiole, fronds | Thailand | Loilong et al. 2012 |
| *Delitschia nypae* | saprobe | fruit pericarp | Thailand | Jayasiri et al. 2019 |
| *Diaporthe* sp. | endophyte | stem | Indonesia | Nasution et al. 2024 |
| *Diaporthe arecae* | possible pathogen | leaves | Thailand | This study |
| *Diaporthe hongkongensis* | saprobe |  | India (Andaman Islands) | Thavamani et al. 2018 |
| *Dictyocheirospora heptaspora* | saprobe | petiole | Thailand | Jones et al. 2006; Boonmee et al. 2016 |
| *Dictyosporium elegans* | saprobe | petiole | Thailand | Nakagiri 1993; Devadatha et al. 2021 |
| *Dictyosporium pelagicum* | saprobe | rachis | Brunei Darussalam | Hyde & Alias 2000 |
| *Didymella* sp. | saprobe |  | Philippines | Besitulo-Donoso 2016 |
| *Diplodia* sp. | saprobe | petiole, fronds | Thailand | Loilong et al. 2012 |
| *Ellisembia crassispora (Sporidesmium crassisporum)* | saprobe | petiole | Brunei Darussalam | Hyde & Sarma 2006; Devadatha et al. 2021 |
| *Exserohilum rostratum* | saprobe | petiole | Malaysia | Devadatha et al. 2021 |
| *Fasciatispora nypae* | saprobe | rotten frond | Australia, Brunei Darussalam, Thailand | Hyde et al. 1991a, Hyde et al. 1995 |
| *Fasciatispora petrakii* | saprobe | petiole | Malaysia | Hyde et al. 1992c, Hyde & Alias 2000; Alias et al. 2010 |
| *Frondicola tunitricuspis* | saprobe | decaying fronds | Brunei Darussalam, Malaysia | Hyde et al. 1992a; Alias et al. 2010; Hyde & Alias 2000; Hyde & Sarma 2006 |
| *Fusarium* sp. | endophyte | frond | Indonesia | Nasution et al. 2024 |
| *Halenospora varia* | saprobe | petiole | Thailand | Loilong et al. 2012; Devadatha et al. 2021 |
| *Halocyphina villosa* | saprobe | inflorescence, rachis | Brunei Darussalam, Indonesia, Malaysia | Hyde et al. 1992c, Hyde & Alias 2000 |
| *Halomassarina thalassiae* | saprobe | petiole | Thailand | Devadatha et al. 2021 |
| *Halorosellinia oceanica* | saprobe |  | Philippines | Besitulo-Donoso 2016 |
|  |  |  |  |  |
| *Halosphaeriopsis alopallonellum (Trichocladium alopallonellum)* | saprobe | petiole, rachis | Brunei Darussalam | Hyde & Alias 2000; Devadatha et al. 2021 |
| *Halosphaeriopsis mediosetigera (Trichocladium achrasporum)* | saprobe | petiole | Australia, Thailand | Loilong et al. 2012; Devadatha et al. 2021 |
| *Helicascus nypae* | saprobe | dead fronds | Malaysia, Brunei Darussalam, Thailand | Hyde et al. 1991b |
| *Helicoma hongkongense* | saprobe | petiole | Thailand | Loilong et al. 2012; Devadatha et al. 2021 |
| *Helicoma hyalonemum* | saprobe | petiole | Thailand | Loilong et al. 2012; Devadatha et al. 2021 |
| *Helicoma pannosum* | saprobe | petiole | Thailand | Loilong et al. 2012; Devadatha et al. 2021 |
| *Helicorhoidion nypicola* | saprobe | base petiole | Brunei Darussalam, Cuba, Malaysia, Thailand | Hyde et al. 1999a; Hyde & Sarma 2006; Samón-Legrá et al. 2014 |
| *Helicosporium pannosum* | saprobe | petiole, frond | Thailand | Loilong et al. 2012; Devadatha et al. 2021 |
| *Herpotrichia nypicola* | saprobe | base petiole | Malaysia | Hyde et al. 1999a; Alias et al. 2010; Hyde & Alias 2000 |
| *Humicola* sp. | endophyte | frond | Indonesia | Nasution et al. 2024 |
| *Hydea pygmea (=Cirrenalia pygmea)* | saprobe | rachis | Brunei Darussalam | Hyde & Alias 2000; Devadatha et al. 2021 |
| *Hypoxylon* sp. | endophyte | fronds | Indonesia | Nasution et al. 2024 |
| *Kallichroma tethys* | saprobe | frond, petiole | Thailand | Loilong et al. 2012 |
| *Leptosphaeria australiensis* | saprobe | rachis | Brunei Darussalam | Hyde & Alias 2000 |
| *Leptosphaeria nypicola* | saprobe | base petiole, decaying wood | Malaysia, Thailand | Hyde et al. 1999a, Alias et al. 2010 |
| *Lignincola laevis* | saprobe | petiole | Malaysia, Philippines, Thailand | Devadatha et al. 2021 |
| *Lignincola nypae* | saprobe | base petiole | Malaysia, Philippines, Thailand | Hyde et al. 1999a; Alias et al. 2010; Besitulo et al. 2010 |
| *Lineolata rhizophorae* | saprobe | decaying wood, roots | Thailand | Devadatha et al. 2021 |
| *Linocarpon angustatum* | saprobe | petiole | Brunei Darussalam, Malaysia, Philippines, Thailand | Hyde et al. 1999b; Hyde & Alias 1999; 2000; Alias et al. 2010; Besitulo et al. 2010; Hyde & Sarma 2006; Suetrong et al. 2017 |
| *Linocarpon appendiculatum* | saprobe | rotten fronds | Brunei Darussalam, Papua New Guinea, Indonesia (Sumatra), Malaysia, Philippines, Papua Guinea, Thailand | Hyde et al. 1992a; Alias et al. 2010; Besitulo et al. 2010 Hyde 1988b, 1989b, 1992a, 1992c; Hyde & Alias 2000; Hyde & Sarma 2006 |
| *Linocarpon bipolare* | saprobe | fronds | Brunei Darussalam, Thailand | Hyde et al. 1992a; Alias et al. 2010, Hyde 1992a, 1992c; Hyde & Alias 2000; Hyde & Sarma 2006; Suetrong et al. 2017 |
| *Linocarpon longisporum* | saprobe | fronds | Brunei Darussalam, Philippines, Malaysia | Hennings 1908; Hyde et al. 1992a; Alias et al. 2010; Hyde 1988b, 1992a, 1992c; Hyde & Alias 2000; Hyde & Sarma 2006 |
| *Linocarpon nypae (=Ophiobolus nipae)* | saprobe | dead petioles | Brunei Darussalam, Philippines, Thailand | Hyde et al. 1992a; Alias et al. 1995; 2010; Hyde 1988b, 1992a, 1992c; Hyde & Alias 2000; Hyde & Sarma 2006 |
| *Linocarpon pandani* | saprobe | petiole | Brunei Darussalam, Philippines, China (Taiwan) | Hyde 1988a, 1988b, 1989, 1992a |
| *Longicorpus striatisporus* | saprobe | wood | Brunei Darussalam, Thailand | Zhang et al. 2019; Devadatha et al. 2021 |
| *Lophodermium nypae* | saprobe | leaves | Thailand | Zhang et al. 2024 |
| *Lulworthia medusa* | saprobe | petiole, fronds, inflorescence, rachis | Brunei Darussalam, Indonesia, Malaysia | Hyde 1992a; Devadatha et al. 2021 |
| *Lyomyces sambuci (=Xylodon sambuci)* | saprobe | petiole | Thailand | Devadatha et al. 2021 |
| *Matsusporium tropicale (=Cirrenalia tropicalis)* | saprobe | petiole, rachis | Brunei Darussalam | Hyde & Alias 2000; Devadatha et al. 2021 |
| *Melomastia marinospora (=Dyfrolomyces marinosporus, Saccardoella marinospora)* | saprobe | frond | Brunei Darussalam | Hyde 1992d |
| *Microthyrium* sp. | saprobe | frond | Philippines, Thailand | Loilong et al. 2012; Besitulo-Donoso 2016 |
| *Monodictys* sp. | saprobe | frond | Thailand | Loilong et al. 2012 |
| *Mortierella* sp. | saprobe, endophyte | frond, petiole | Brunei Darussalam, Indonesia | Devadatha et al. 2021; Nasution et al. 2024 |
| *Natantispora retorquens* (=*Halosarpheia retorquen*s) | saprobe | rachis, wood | Brunei Darussalam, Malaysia | Hyde 1992a; Hyde & Alias 2000; Devadatha et al. 2021 |
| *Nemania palmarum* | saprobe | petiole | Thailand | Zhang et al. 2024 |
| *Neodeightonia nypae* | saprobe | decaying rachis | Thailand | Zhang et al. 2024 |
| *Neohalosarpheia marina (=Halosarpheia marina)* | saprobe | petiole | Brunei Darussalam, Malaysia | Hyde et al. 1992a; Devadatha et al. 2021 |
| *Neolinocarpon globosicarpum* | saprobe | fronds | Brunei Darussalam; Bahamas, Brazil,  Malaysia, Thailand | Hyde et al. 1992a; Alias et al. 2010; Hyde 1992c, 1993, Hyde & Alias 2000; Hyde & Sarma 2006 |
| *Neolinocarpon nypicola* | saprobe | petiole | Malaysia | Hyde et al. 1999b |
| *Neptunella longirostris (=Lignincola longirostris)* | saprobe | inflorescence | Brunei Darussalam, Malaysia | Hyde 1992a; Hyde & Alias 2000 |
| *Nereiospora cristata (Piricauda pelagica) (=Monodictys pelagica)* | saprobe | rachis | Brunei Darussalam | Hyde 1992a; Hyde & Alias 2000 |
| *Nipicola carbospora* | saprobe | fronds | Brunei Darussalam, Malaysia | Hyde et al. 1992b; Devadatha et al. 2021 |
| *Nipicola selangorensis* | saprobe | fronds | Malaysia | Hyde 1994a; Hyde & Taylor 1996 |
| *Nypaella frondicola* | saprobe | fronds | Cambodia, China, Indonesia, Laos, Malaysia, Philippines, Vietnam | Hyde & Sutton 1992 |
| *Okeanomyces cucullatus (Periconia prolifica)* | saprobe |  | Philippines | Besitulo-Donoso 2016 |
| *Oxydothis nypae* | saprobe | fronds | Brunei Darussalam, Malaysia, Thailand | Hyde et al. 1994b; Alias et al. 2010; Hyde 1992c, 1993, 1994b, Hyde & Alias 2000; Hyde & Nakagiri 1989; Jones et al. 2006b |
| *Oxydothis nypicola* | saprobe | rotten petiole | Brunei Darussalam, Malaysia | Hyde et al. 1994b; Alias et al. 2010; Hyde & Alias 2000; Poonyth et al. 2000 |
| *Paecilomyces* sp. | saprobe, endophyte | frond | Indonesia, Malaysia, Thailand | Devadatha et al. 2021; Nasution et al. 2024 |
| *Panorbis viscosus (=Halosarpheia viscosa)* | saprobe | rachis | Brunei Darussalam, Malaysia | Hyde 1992a; Hyde & Alias 2000 |
| *Papulaspora* sp. | endophyte | fruit mesocarp | Indonesia | Nasution et al. 2024 |
| *Paraaniptodera longispora* (=*Aniptodera longispora)* | saprobe | petiole, fronds | Thailand | Loilong et al. 2012; Devadatha et al. 2021 |
| *Paravamsapriya nypae* | saprobe | rachis | Thailand | Zhang et al. 2024 |
| *Payosphaeria* sp. | saprobe |  | Philippines | Besitulo-Donoso 2016 |
| *Penicillium* sp. | endophyte | stem | Indonesia | Nasution et al. 2024 |
| *Pestalotiopsis* sp. | saprobe, endophyte | leaves, stem, fruit mesocarp | Indonesia, Malaysia, Thailand | Giatgong 1980; Choo et al. 2015; Nasution et al. 2024 |
| *Pestalotiopsis nypae* | possible pathogen | leaves | Thailand | This study |
| *Phialophorophoma litoralis* | saprobe | dead wood | Brunei Darussalam | Hyde 1992a |
| *Phomatospora nypae* | saprobe | base petiole | Malaysia | Hyde et al. 1993; Hyde & Alias 2000; Alias et al. 2010 |
| *Phomatospora nypicola* | saprobe | base petiole | Malaysia | Hyde et al. 1999; Hyde & Alias 2000 |
| *Plectophomella nypae* | saprobe | fronds | Cambodia, China, Indonesia, Laos, Malaysia, Philippines, Vietnam | Hyde & Sutton 1992; Alias et al. 2010 |
| *Pleurophomopsis nypae* | saprobe | fronds | Cambodia, China, Indonesia, Laos, Malaysia, Philippines, Vietnam | Hyde & Sutton 1992 |
| *Pontogeneia* sp. | saprobe |  | Philippines | Besitulo-Donoso 2016 |
| *Pseudolignincola siamensis* | saprobe |  | Thailand | Jones et al. 2006 |
| *Pseudothailandiomyces nypae* | saprobe | submerged petioles | Thailand | Zhang et al. 2024 |
| *Remisporiopsis macrocephala (=Cirrenalia macrocephala)* | saprobe | petiole | Philippines | Besitulo et al. 2010; Devadatha et al. 2021 |
| *Rhipidocarpon javanicum* | saprobe | leaf, petiole | Indonesia, Papua New Guinea, Philippines | Shaw 1984; Teodoro 1937; Hyde & Alias 2000 |
| *Rimora mangrovei* | saprobe | petiole |  | Devadatha et al. 2021 |
| *Saagaromyces abonnis (=Halosarpheia abonnis)* | saprobe | rachis | Brunei Darussalam | Hyde 1992a; Devadatha et al. 2021 |
| *Salsuginea ramicola* | saprobe | petiole | Brunei Darussalam, Indonesia, Thailand | Devadatha et al. 2021 |
| *Sammeyersia grandispora* | saprobe | petiole | Brunei Darussalam, Malaysia, Philippines  Papua New Guinea, Thailand | Devadatha et al. 2021 |
| *Savoryella aquatica* | saprobe | petiole | Brunei Darussalam, Thailand | Hyde & Sarma 2006 |
| *Savoryella lignicola* | saprobe | petiole, inflorescence, rachis | Malaysia | Hyde & Alias 2000; Devadatha et al. 2021 |
| *Savoryella nypae* | saprobe | submerged rachis, petiole | Brunei Darussalam, Cuba, Malaysia, China (Taiwan), Thailand | Zhang et al. 2019; Alias et al. 2010; Hyde 1988; Hyde & Sarma 2006, Pang & Jheng 2012; Samón-Legrá et al. 2014; Suetrong et al. 2017 |
| *Savoryella paucispora* | saprobe | petiole | Thailand | Loilong et al. 2012; Devadatha et al. 2021 |
| *Schizophyllum commune* | saprobe | petiole | Thailand | Loilong et al. 2012; Supaphon et al. 2014; Devadatha et al. 2021 |
| *Sclerococcum haliotrephum (=Dactylospora haliotrepha)* | saprobe | petiole, fronds | Thailand | Loilong et al. 2012; Devadatha et al. 2021 |
| *Striatiguttula nypae* | saprobe | decayed rachis | Thailand | Zhang et al. 2019; Devadatha et al. 2021 |
| *Swampomyces* sp. | saprobe | fronds | Brunei Darussalam, Malaysia, Philippines, Thailand | Loilong et al. 2012 |
| *Tetraploa aristata* | saprobe |  | Malaysia | Devadatha et al. 2021 |
| *Thozetella nivea* | saprobe | petiole | Thailand | Loilong et al. 2012; Devadatha et al. 2021 |
| *Tirispora unicaudata* | saprobe | petiole | Egypt, Hong Kong  India, Malaysia, China (Taiwan) | Loilong et al. 2012; Devadatha et al. 2021 |
| *Tirisporella beccariana* | saprobe | petiole | Malaysia, Philippines, Thailand | Jones et al. 1996; Suetrong et al. 2017; Alias et al. 2010; Suetrong et al. 2017 |
| *Trematosphaeria mangrovis* | saprobe | petiole | Philippines, Thailand | Devadatha et al. 2021 |
| *Trematosphaeria lineolatispora* | saprobe | frond | Thailand | Loilong et al. 2012 |
| *Trichocladium nypae* | saprobe | base petiole | Brunei Darussalam, Malaysia | Hyde et al. 1999a |
| *Torulaspora nypae* | saprobe | inflorescence sap | Thailand | Kaewwichian et al. 2020 |
| *Tubeufia* sp. | saprobe | fronds, petiole | Thailand | Loilong et al. 2012 |
| *Vaginatispora nypae* | saprobe | fruit pericarp, rachis | Thailand | Jayasiri et al. 2019 |
| *Vaginatispora palmae* | saprobe | petiole | Thailand | Hyde et al. 2020 |
| *Vanakripa* sp. | saprobe | fronds, petiole | Thailand | Loilong et al. 2012 |
| *Verruculina enalia* | saprobe | rachis | Brunei Darussalam | Hyde & Alias 2000; Devadatha et al. 2021 |
| *Vibrissea nypicola* | saprobe | base petiole | Malaysia, Philippines, Thailand | Hyde et al. 1999a; Hyde & Alias 2000; Alias et al. 2010 |
