# Supplementary material for: Putative palm pathogens: Novel species and new host records of leaf disease-associated microfungi (Ascomycota) on Nypa fruticans in Thailand
Source: MycoKeys. 2026 Apr 6;130:315–53. doi: 10.3897/mycokeys.130.175665 (PMC13077315; doi:10.3897/mycokeys.130.175665)
Supplement: Supplementary material 3 — Single-gene phylogenetic trees of the novel species [file mycokeys-130-315-s003.pdf]

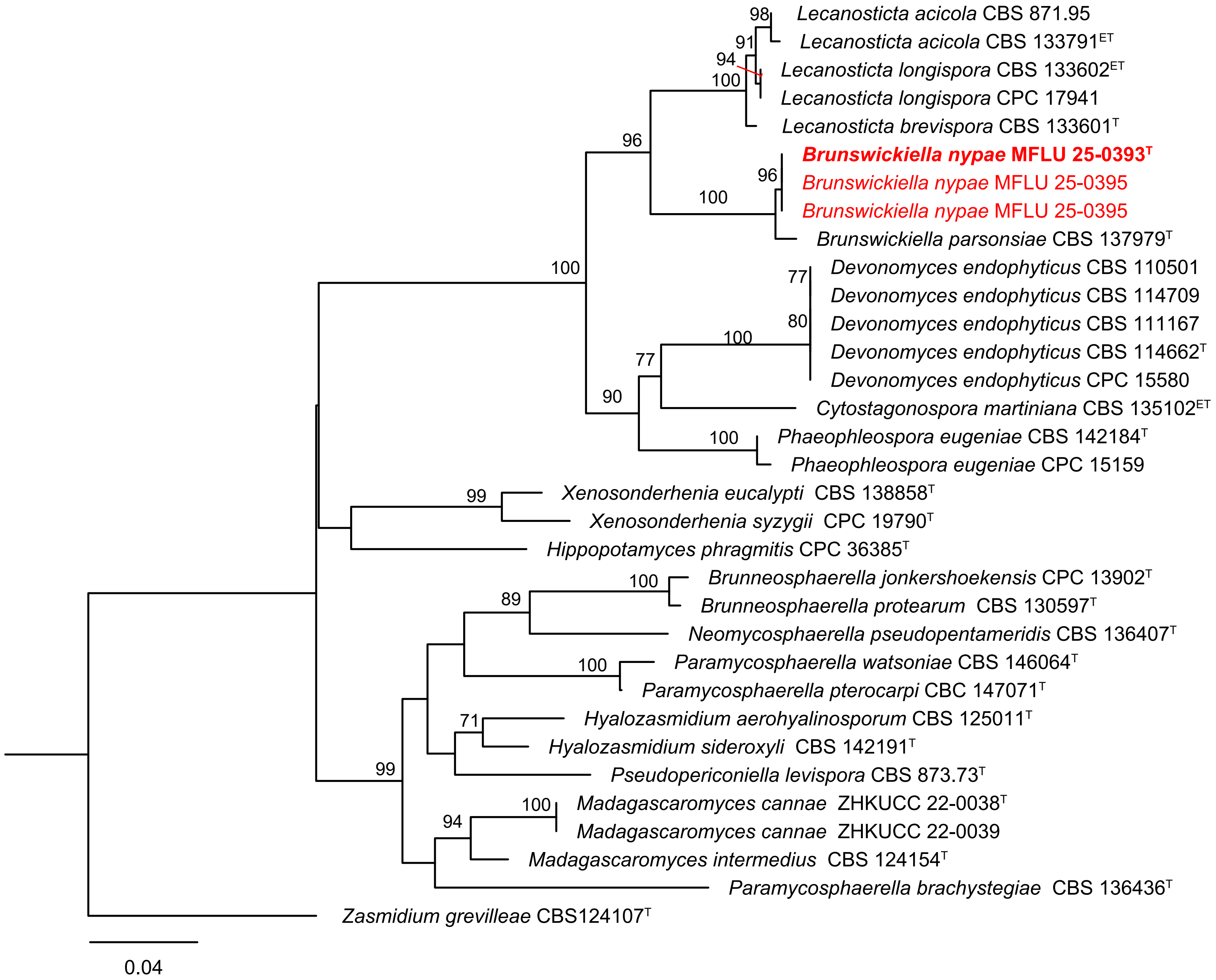

FIG. S1 *Brunswickiella:* ITS

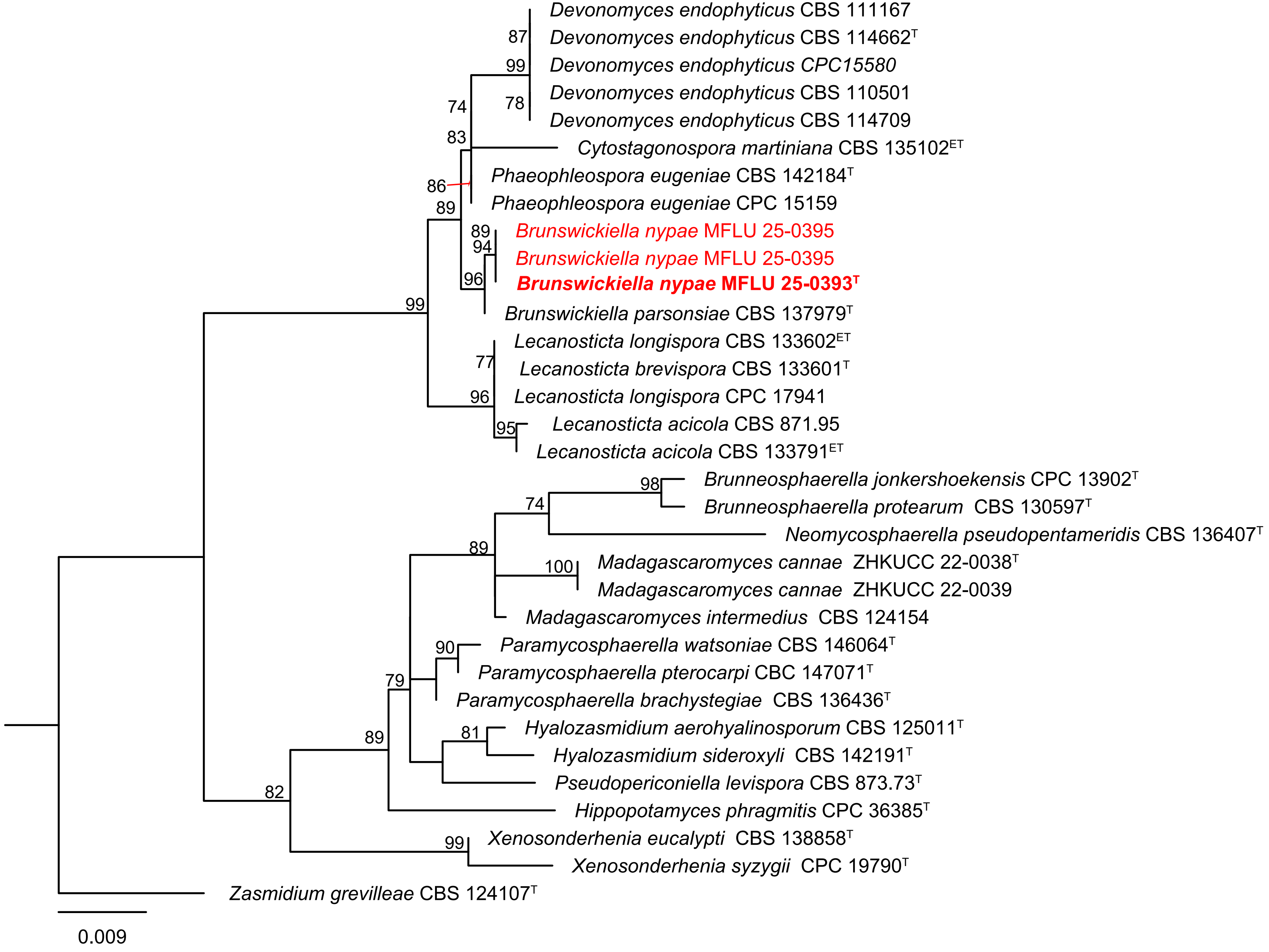

FIG. S2 *Brunswickiella:* LSU

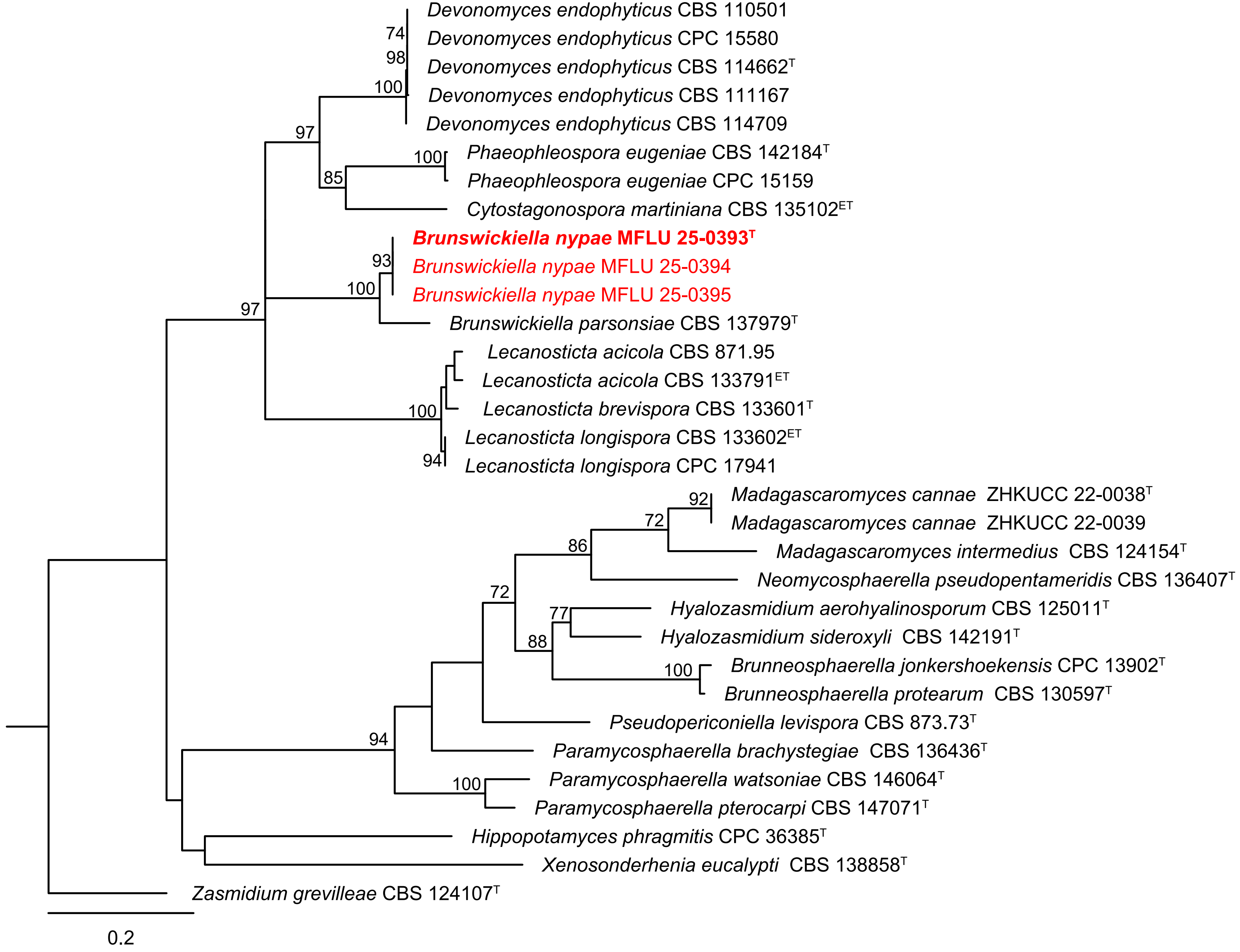

FIG. S3 *Brunswickiella:* RPB2

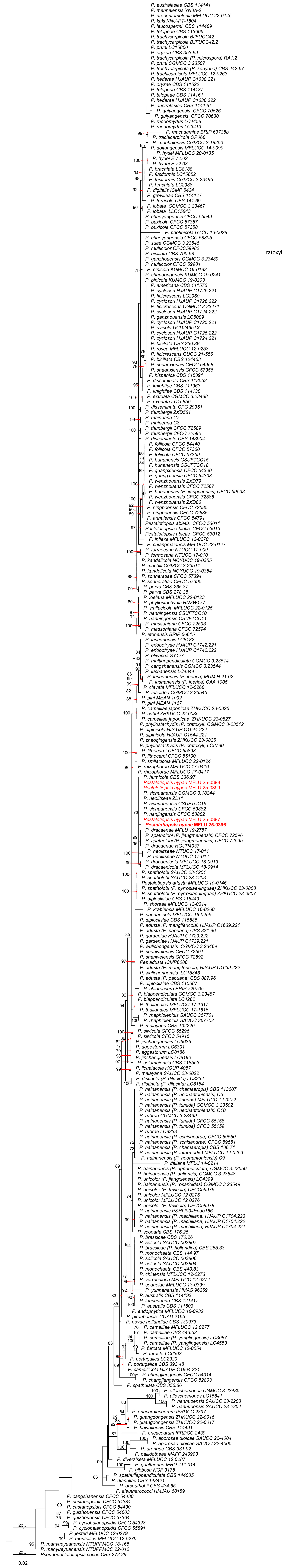

FIG. S4 *Pestalotiopsis*: ITS

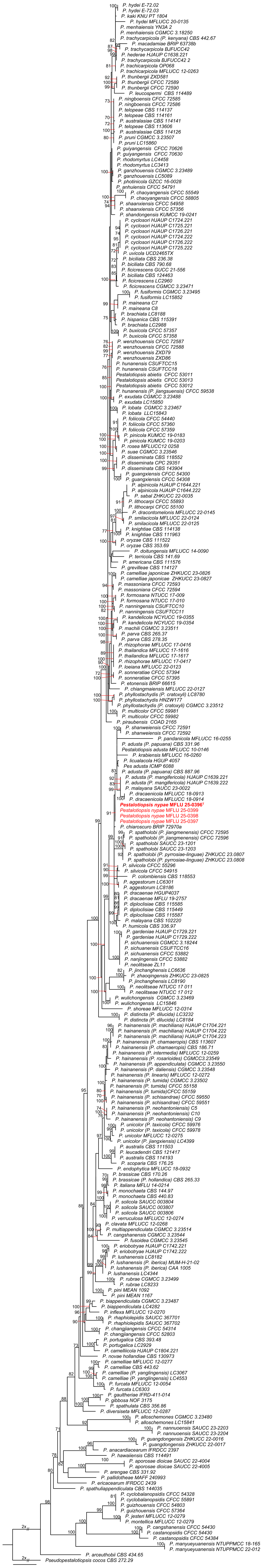

FIG. S5 *Pestalotiopsis*: TEF1

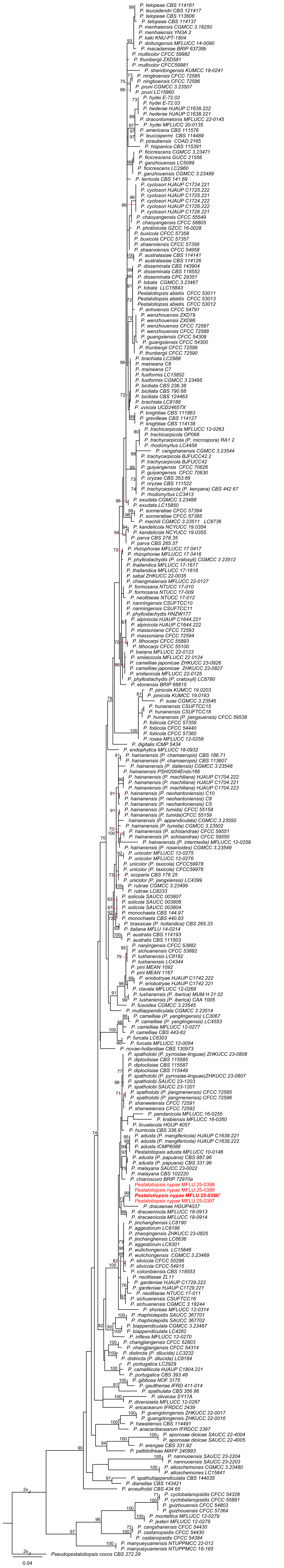

FIG. S6 *Pestalotiopsis*: TUB2
